# 1,2,3-Triazole Hybrids Containing Isatins and Phenolic Moieties: Regioselective Synthesis and Molecular Docking Studies

**DOI:** 10.3390/molecules29071556

**Published:** 2024-03-30

**Authors:** Loredana Maiuolo, Matteo Antonio Tallarida, Angelo Meduri, Giulia Fiorani, Antonio Jiritano, Antonio De Nino, Vincenzo Algieri, Paola Costanzo

**Affiliations:** 1 Department of Chemistry and Chemical Technologies, University of Calabria, 87036 Rende, Italy; maiuolo@unical.it (L.M.); antonio.jiritano@unical.it (A.J.); denino@unical.it (A.D.N.); 2 RINA Consulting—Centro Sviluppo Materiali SpA, Via di Castel Romano 100, 00128 Rome, Italy; matteo.tallarida@rina.org; 3 RINA Consulting—Centro Sviluppo Materiali SpA, Zona Industriale San Pietro Lametino, Comparto 1, 88046 Lamezia Terme, CZ, Italy; angelo.meduri@rina.org; 4 Department Molecular Sciences and Nanosystems, University Ca’ Foscari Venezia, 30172 Mestre, VE, Italy; giulia.fiorani@unive.it; 5 IRCCS NEUROMED—Istituto Neurologico Mediterraneo, Via Atinense 18, 86077 Pozzilli, IS, Italy

**Keywords:** triazole hybrids, isatin hybrids, CuAAC

## Abstract

The synthesis of hybrid molecules is one of the current strategies of drug discovery for the development of new lead compounds. The 1,2,3-triazole moiety represents an important building block in Medicinal Chemistry, extensively present in recent years. In this paper, we presented the design and the synthesis of new 1,2,3-triazole hybrids, containing both an isatine and a phenolic core. Firstly, the non-commercial azide and the alkyne synthons were prepared by different isatines and phenolic acids, respectively. Then, the highly regioselective synthesis of 1,4-disubstituted triazoles was obtained in excellent yields by a click chemistry approach, catalyzed by Cu(I). Finally, a molecular docking study was performed on the hybrid library, finding four different therapeutic targets. Among them, the most promising results were obtained on 5-lipoxygenase, an enzyme involved in the inflammatory processes.

## 1. Introduction

The hybrid molecules, obtained by the structural combination of different pharmacophoric sub-unities of two or more known bioactive derivatives, represent an excellent stratagem to design new pharmaceuticals with more potent properties while maintaining pre-selected characteristics of the original templates. The interest of researchers towards this class of molecules has undergone a large escalation in recent years with the idea that the hybrids can interact with more than one biological target, improving their activity, gaining higher selectivity, and possibly reducing drug resistance [1,2,3].

Among the myriad of compounds with biological profiles, 1,2,3-triazoles are some of the most extensively used scaffolds in pharmaceuticals for their special properties such as hydrogen bonding capability, rigidity, stability as well as a wide range of biological activities [4,5,6,7,8,9,10]. Moreover, their synthesis is very simple by the click chemistry approach [11,12]. In more detail, the concept of click chemistry (Azide-Alkyne Cycloaddition) was introduced by Huisgen in 1960 [13], who reported the 1,3-dipolar cycloaddition between a terminal alkyne and an azide to produce a mixture of 1,4- and 1,5-substituted triazole. Then, the introduction of a catalyst such as cuprum for the Cu(I)-catalyzed azide-alkyne cycloaddition (CuAAC) and ruthenium for the Ru(II)-catalyzed reaction (RuAAC) produced 1,4- and 1,5-disubstituted regioisomers, respectively [14,15]. In the last years, a lot of other metal catalysts have been developed for the synthesis of substituted 1,2,3-triazoles in a regioselective manner [16]. 

In addition to triazoles, other nitrogen-containing molecular architectures have been widely utilized as versatile materials in pharmaceutical discovery. Oxindole derivatives and in particular isatin have received wide interest due to the presence of different functionalizable groups in their structure [17,18]. The isatin nucleus was explored in medicinal chemistry for the construction of compounds with antibacterial [19], anticancer [20], antiviral [21], and antioxidant properties [22]. 

Recently, the combination of two structurally different heterocycles in a single molecule to form hybrids, so-called molecules with “two heads”, have become very popular [23]. Generally, this typology of molecules is classified into three types: merged, fused, and conjugated molecular hybrids [24]. In particular, the latter are usually separated by a distinct linker group that is not found in either of the selective sub-units, and the 1,2,3-triazoles could be an ideal building block to this aim, due to its stability.

On the other hand, the combination of these heterocyclic moieties with an additional antioxidant portion on the backbone of the molecule could make the resulting hybrid compound even more potent. In fact, antioxidant activity underlies many biological properties, e.g., antiviral, anti-inflammatory, antibacterial, antiatherosclerotic, anticancer and neuroprotective [25,26], also considering that, for example, oxidative stress and imbalance of antioxidant properties are often the cause of cancer and cardiovascular diseases [27]. 

In general, the presence of more than one hydroxyl group bonded to a benzene ring, especially in the -*ortho* and -*para* positions, confers stronger antioxidant activity to the molecule. In particular, phenolic acids in which the aromatic ring is substituted with two or more hydroxyl groups, i.e., caffeic or gallic acid, have demonstrated more significant antioxidant properties than monophenols [28]. 

In this work, 1,2,3-triazoles conjugated with isatins and antioxidant molecules were synthesized by 1,3-dipolar cycloaddition in a regioselective manner by Cu(I) catalysis to promote the obtaining only of 1,4-disubstituted triazole derivatives. In this way, we obtained new conjugated molecular hybrids, containing the 1,2,3 triazole moiety as a linker between the two heads. This choice derived from the innumerable biological applications of 1,4-derivatives probably due to their spatial arrangement that permits them to form various secondary interactions with the active site of enzyme target (dipole-dipole interactions, hydrogen bonds, π–stacking, etc.) [29].

In addition, a series of molecular docking studies was performed on the full set of the synthesized products. Eight relevant targets were selected and studied to evaluate the potential biological activity of our hybrids. 

## 2. Results and Discussion

### 2.1. Synthesis

**Figure 1:**
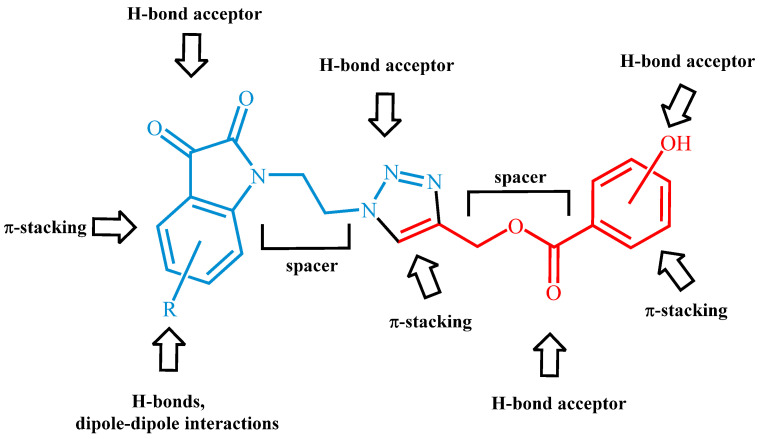
Generic structure of synthesized 1,2,3 triazole hybrids.

With the aim of realizing hybrids of 1,2,3-triazoles as the core between isatins and antioxidant molecules to enhance the sites interactivity with enzymatic tasks of biological targets (see Figure 1) and considering our great experience on the heterocycle synthesis by 1,3-dipolar cycloadditions [30,31,32,33,34,35], we selected the Cu(I)-catalyzed azide-alkyne cycloaddition (CuAAC) to perform 1,4-disubstituted triazole derivatives. Moreover, our molecules were projected with short spacers between the various active sub-units to make these compounds more flexible and adaptable in the enzymatic cavities (Figure 1).

**Scheme 1:**
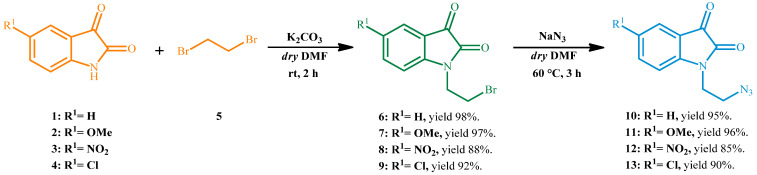
Generic synthesis of variously substituted 1-(2-azidoethyl)isatins **10**–**13**.

In order to develop a regioselective copper(I)-catalyzed 1,3-dipolar cycloaddition reaction, a preventive preparation of opportune dipoles and dipolarophiles was necessary. For one, 1-(2-azidoethyl)isatins were used as 1,3-dipoles and were synthesized according to the reactions reported in Scheme 1. 

The procedure for the synthesis of N-bromoalkyl derivatives was based on a modified method found in the literature [36]. In detail, opportune isatins **1**–**4** were reacted with 1,2-dibromoethane **5** and potassium carbonate in dry DMF at room temperature to give the corresponding 1-(2-bromoethyl)isatins **6**–**9** with excellent isolated yields. As observed in Scheme 1, the presence of different substituent groups in the 5-position does not seem to deeply affect the reactivity of the isatins **1**–**4** towards alkylation because comparable reaction yields were observed for all cases. The series of isolated bromoalkyl products **6**–**9** were subsequently reacted with NaN_3_ to form corresponding 1-(2-azidoethyl)isatin **10**–**13** with high reaction yield [37]. 

Successively, three phenolic acid propargyl esters **20**–**22** were chosen as dipolarophiles and their synthesis and results are illustrated in Table 1.

The synthetic procedure started from the chlorination of the phenolic acids **14**–**16** by thionyl chloride at reflux for six hours, obtaining the corresponding acyl chlorides **17**–**19**, which were used, without isolation, with propargyl alcohol to give the desired propargyl esters **20**–**22** in quantitative yields. 

With the precursors in our hands, we selected the cycloaddition between 1-(2-azidoethyl)isatin **10** and propargyl 3,4,5-trihydroxybenzoate **20** as the model system to optimize the reaction conditions. We chose the Cu(II)sulfate pentahydrate/sodium ascorbate (CuSO*_4_* 5H*_2_*O/Na-Ascorbate) as the catalytic system due to its high compatibility with non-anhydrous organic solvents, as reported in the literature [38].

The changes in solvents, reagent molar ratios, catalyst molar ratios, temperature and time are summarized in Table 2. 

Initially, we decided to apply the reaction conditions used on triazole hybrids found in the literature [39], isolating 1,4-disubstituted 1,2,3-triazole **23**. In this case, we observed only a 22% yield of the product after 48 h at room temperature with degradation of alkyne **20** (Table 2, entry 1). Then, we increased the temperature to 60 °C, resulting in a 67% yield of **23** after only 5 h (Table 2, entry 3). It is noteworthy that no significant variation was observed when the reaction was extended up to 24 h. Moreover, higher temperatures showed only the degradation of reagent **20**. At this point, we decided to replace the DMF with EtOH, i-PrOH, and t-BuOH (Table 2, entries 4–6), classic conventional solvents commonly used in CuAAC. However, the reaction yields were lower (38–45%) for all three cases, probably due to the minor solubility of CuSO_4_ 5H_2_O and Na-Ascorbate in the reaction medium. Instead, we performed a subsequent experiment (Table 2, entry 7) in which the reaction was carried out in DMF without added water provided product **23** with a yield comparable to that in the presence of water (Table 2, entry 3). Therefore, we decided to continue the investigation only in DMF as a solvent and by doubling the molar quantity of catalyst and co-catalyst, observing an increase in the reaction yield until 90% (Table 2, entry 8). Then, the amount of azide **10** was reduced to the stoichiometric ratio 1:1 (Table 2, entry 9), obtaining a result perfectly comparable to the previous one. To complete the control reaction we performed the reaction in the absence of a catalytic system (Table 2, entry 10) and we observed only traces of the final product.

Finally, to verify the goodness of the developed synthetic procedure, we reproduced the reaction on a gram-scale, obtaining similar reaction yields (Table 2, entry 11). Ultimately, the 1,3-dipolar cycloaddition was carried out in ionic liquid [mPy]OTf [40,41] (Table 2, entry 12), with the aim of recycling the solvent and catalytic system after the reaction. Unfortunately, in these conditions, only traces of product **23** were observed (Table 2, entry 12). 

Once the reaction conditions were optimized (Table 2, entry 9), we extended the investigation by using the 1-(2-azidoethyl)isatins **10**–**13** and the propargyl esters **20**–**22** of phenolic acids to synthesize a new series of 1,4-disubstituted 1,2,3-triazoles hybridized molecules **23**–**34** (Table 3). 

As shown in Table 3, phenolic acid propargyl esters **20**–**22** exhibited high reactivity towards copper(I)-catalyzed 1,3-dipolar cycloaddition (CuAAC), resulting in high reaction yields in all cases. As you can see, the reactivity of the azides appears to be slightly dependent on the nature of the substituent in the 5-position. More specifically, when electron-withdrawing groups as -NO_2_ and -Cl were present on the backbone of the isatins, moderately lower yields were observed for reaction with all three dipolarophiles (Table 3, entries 3–4, 7–8, 11–12), if compared to the presence of the electron-donor group -OMe (Table 3, entries 2, 6, 10). It is worth noting that only a single 1,4-disubstituted 1,2,3-triazole regioisomer was obtained for all synthesized compounds, demonstrating the high regioselectivity of the well-known copper(I)-catalyzed azide-alkyne cycloaddition (see Supplementary Materials for the ^1^H NMR of reaction crude of **23**) [42,43]. 

**Scheme 2:**
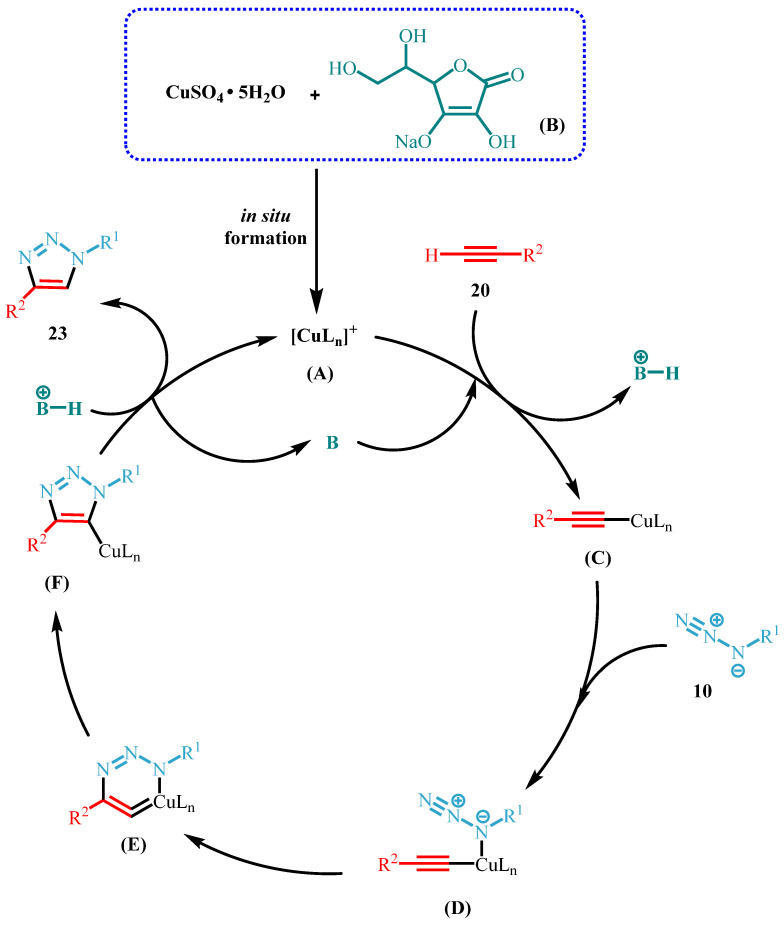
Proposed catalytic cycle for copper(I)-catalyzed azide-alkyne cycloaddition.

At this point, we propose a possible reaction mechanism for the reaction between **10** and **20** as illustrated in Scheme 2.

Initially, Cu(II) reacts with Na-ascorbate via a redox reaction to in situ form the Cu(I)-complex catalytic species (**A**), generically indicated as [CuL_n_]^+^. The catalytic cycle begins with the coordination of the Cu(I) (**A**) species to the triple bond of the alkyne **20,** forming a π-complex. The base (**B**), consisting of the excess molar quantity of Na-ascorbate with respect to [CuL_n_]^+^, allows the alkyne deprotonation and the Cu(I) species binds to the sp hybridized carbon (**C**). Subsequently, azide **10** coordinates with the Cu-π-coordinated system with very specific regiochemistry (**D**), and the formation of a six-membered metallacycle occurs (**E**). The metallacycle undergoes a rearrangement whereby an aromatic five-membered cycle (**F**) is formed. Finally, protonation of the copper-linked 1,2,3-triazole causes the C-H bond formation, resulting in the desired 1,4-disubstituted 1,2,3-triazole product **23,** and the catalyst is released [44,45].

### 2.2. Molecular Docking

With the aim of proposing new biological evaluation perspectives to the conjugates obtained, a series of molecular docking studies were carried out following a protocol from our recent study [46]. AutoDock Vina [47] was used, by keeping the docking parameters at default values. The geometries of the triazole conjugates were previously optimized quantum mechanically. The in silico studies were performed over eight targets retrieved from the Protein Data Bank (http://www.rcsb.org/accessed on 16 December 2023) for the full set of the compounds synthesized in this work. A total number of 104 simulations (96 for the newly synthesized compounds plus eight for reference inhibitors) were performed. The biological targets for the computational study were chosen on the basis of their relevance in medicinal chemistry, being involved in the etiopathogenesis of several diseases. Three specific aspects were taken into account: (1) evidence from the literature on the biological activity displayed by triazole-phenolic acids conjugates [48,49], (2) evidence on the biological activity displayed by triazole-isatin conjugates [42,50,51], (3) evidence on the activity of other relevant triazole hybrids [52,53].

The target 5-lipoxygenase (5-LOX) is a critical enzyme in arachidonic acid (ACD) breakdown and drives the formation of leukotrienes (LTs) [54]. These inflammatory mediators have far-reaching effects, contributing to conditions like asthma, allergic rhinitis, cardiovascular diseases, and even some cancers [55]. Cyclooxygenase-2 (COX-2) is another critical target, and its understanding offered alternatives for treating arthritis with reduced gastrointestinal risk, such as the discovery of celecoxib [56]. Angiotensin-converting enzyme (ACE) became a drug target in the 1970s and still represents a hot topic for specific ACE inhibitors for better blood pressure control and reduced side effects [57]. Type IIA topoisomerases (Top2), enzymes crucial for DNA organization, are targeted in cancer therapy. Humans have two isoforms of Type IIA topoisomerase: Topo IIα and Topo IIβ. The Topo IIα isoform is particularly sensitive to drugs that form complexes with the enzyme and DNA, disrupting cancer cell function [58]. Tubulin, crucial for many cellular functions, is a strategic target for antitumor drugs. By binding to tubulin, these drugs block essential processes, ultimately causing cell death [59]. The enzyme acetylcholinesterase (AChE) breaks down acetylcholine, a crucial brain chemical for memory and learning; its depletion in Alzheimer’s Disease leads to cognitive decline [60]. Lastly, the selected D-alanine-d-alanine ligase (Ddl) target is an enzyme that builds a crucial part of the bacterial cell wall and, for this reason, is a promising target for antibiotic development [61]. For all the computational simulations, a reference ligand from the literature was docked to the biological targets to give a reference binding score (Table 4).

All the docked substrates showed strong potential affinity with the biological targets, which in most cases were superior or comparable to the reference ligands (as highlighted in bold text in Table 4). Moreover, for all the docked compounds, no particular difference in terms of affinity was revealed by varying the functional groups present on the structure. This might be due to the dimensions and the great conformational flexibility of the tested compounds, which mediate the detrimental or positive effect of the substituents.

In this regard, it can be instructive to calculate the average affinity values for all the conjugates **23**–**34** and compare them to the affinity values of the reference compounds (Table 5).

**Figure 2:**
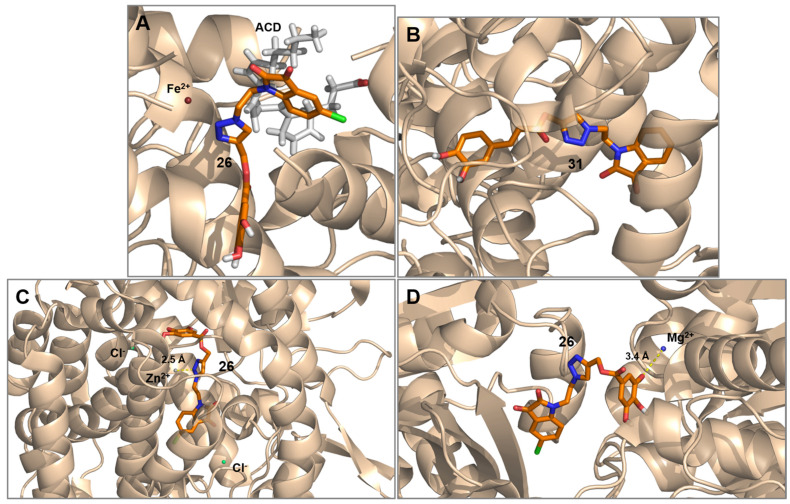
Best docking poses of most interacting triazole conjugates with main four interacting receptors: (**A**) Compound **26** bound to 5-LOX in the presence of arachidonic acid (ACD) (PDB 3V99). (**B**) Compound **31** bound to COX-2 (PDB 4FM5). (**C**) Compound **26** bound to ACE (PDB 1O86). (**D**) Compound **26** bound to tubulin (PDB 1SA0). Ligands and binding residues are shown as sticks.

On the basis of the differences in percentage between our substrates and the literature ligands, we extracted the best docking poses towards the four best resulting targets, namely 5-LOX/ACD, COX-2, ACE, and tubulin. For these cases, we represented the best docking substrates in accordance with the result reported in Table 4. For the 5-LOX/ACD system, substrate **26** interaction was represented, which showed an affinity of −9.0 kcal/mol (Figure 2A). In this case, the choice of substrate 26 was arbitrary since also substrates **29** and **30** gave the same affinity values. As for COX-2 inhibition, substrate **31** was the best-interacting substrate with an affinity of −10.3 kcal/mol (Figure 2B). Lastly, both for ACE and tubulin targets, compound **26** resulted in having the best affinity values of −9.8 (Figure 2C) and −9.7 kcal/mol (Figure 2D), respectively.

The overall displayed affinity results highlight two main aspects. (1) As said, our triazole conjugates show notable conformational flexibility, which results in a better adaptation to the target’s pocket. The presence of versatile spacers like triazoles allows for an easier clickable synthesis of large conjugates containing useful functional groups such as carboxyl (as in the isatin portion) and hydroxyl (as in the phenolic acid portion) groups, capable of taking part in hydrogen bond interactions with target’s backbones. It must be noted that the presence of the carboxyl groups and nitrogen, in particular, can trigger the formation of H-bonds [62]. Larger ligand sizes generally ensure stronger interactions with the target [63,64], but might pose significant problems related to the ligand’s rigidity precluding access to the target’s interacting pocket [65]. Hence, the presence of 1,4-disubstituted spacing triazoles as in our case might solve such rigidity-related issues allowing for more hydrogen bond interactions in the three-dimensional space. This evidence also is in line with the use of triazoles as spacers in the design of new bioactive compounds [66].

(2) The presence of the triazole group not only allows for an extent of conformational flexibility but also prompts the coordination when a metal is present in the active site. In our docking analysis, for three cases over the four high-affinity cases considered, it is worth noting that our substrates showed higher affinity towards active sites wherein a metal was present (5-LOX, ACE, and tubulin). This is related to the capacity of the interacting ligand to offer a strong coordinating site represented by the triazole core, as in the case of substrate **26** with ACE (Figure 2C), or the phenolic acid portion as in the case of substrate **26** with tubulin (Figure 2D). For the ACE interaction, zinc coordination takes place with an interaction distance of 2.5 Å, in line with the recent evidence on the Zn-coordination-related inhibition of β-lactames by triazole ligands [67]. Such theoretical evidence highlights the importance of the coordinating capabilities of the triazole core in medicinal chemistry, which were also discussed in our previous work on the synthesis and docking studies over a new series of pyrimidine-containing 1,5-disubstituted triazoles [31].

As for the coordination of the phenolic acid portion of substrate **26** with Mg^2+^ ion in the tubulin active site, a coordination distance of 3.4 Å suggests a weaker kind of interaction, but results in line with the literature evidence on the phenolic acid’s capabilities to exert a biological activity on the basis of their coordinating capabilities [68].

## 3. Materials and Methods

Commercial starting materials were purchased from Merck (Milano, Italy) or Alfa Aesar (Karlsruhe, Germany) and were used without further purification. Reactions were monitored by TLC using silica plates 60-F264, commercially available from Merck (Milano, Italy). Additionally, ^1^H and ^13^C-NMR spectra and two-dimensional NMR spectra were recorded at 300 and 500 MHz and 125.7 MHz, respectively, in DMSO-d_6_ using tetramethylsilane (TMS) as internal standard (Bruker ACP 300 MHz and Bruker Avance 500 MHz with a 5 mm TBO probe, Rheinstetten, Germany). Chemical shifts are given in parts per million and coupling constants in Hertz. The purity and the regiochemistry were established by NMR spectra (^1^H NMR experiments). High-resolution mass spectra (HRMS) were recorded with a Bruker Compact QTOF instrument (Bruker, Billerica, MA, USA). HRMS spectra were acquired in positive ion mode, with a mass resolution of 30,000. Mass calibration was performed with a solution of sodium formate clusters and processed in HPC mode. Spectra acquisition was performed in flow injection, with a full scan mode in the range of 30 to 1000 *m*/*z*. N_2_ was the source of dry gas (V = 4 L/min, T = 180 °C). The ion formula of each compound was calculated with the Smart Formula tool of the Bruker software platform (Compass data analysis 4.4), analyzing the isotopic pattern ratio with 4 mDa mass confidence. Additionally, for HRMS analysis, all samples were dissolved in MeOH. 

Synthesis and characterization of variously substituted 1-(2-bromoethyl)isatins **6**–**9** and variously substituted 1-(2-azidoethyl)isatins **10**–**13** are realized by modified literature procedures [36,37], respectively, and reported in the Supplementary Materials. Phenolic acid propargyl esters **20**–**22** were prepared with the procedures reported in the Supplementary Materials.

### 3.1. General Procedure for Synthesis of 1,4-Disubstituted 1,2,3-Triazoles ***23***–***34***

In a 50 mL two-necked round-bottomed flask, equipped with a bubble condenser and a magnetic stir bar, the appropriate phenolic acid propargyl ester **20**–**22** (1.20 mmol) and the opportune 1-(2-azidoethyl)isatin **10**–**13** (1.20 mmol) in DMF (20 mL) were dissolved. Subsequently, copper sulfate pentahydrate (0.24 mmol) and sodium ascorbate (0.96 mmol) were added. The reaction mixture was heated at 60 °C for 5 h under stirring and monitored by TLC. The mixture was evaporated under reduced pressure by azeotrope with toluene and then with ethanol, and the obtained oily crude was purified by flash chromatography using CHCl_3_:MeOH (8:2 *v*/*v*) to furnish the solid products **23**–**34** in good yields.

**(1-(2-(isatin-1-yl)ethyl)-1,2,3-triazol-4-yl)methyl 3,4,5-trihydroxybenzoate (23).** Orange solid, yield = 91%. ^1^H NMR (DMSO-d_6_, 300 MHz): δ (ppm) 4.13 (t, J = 5.60 Hz, 2H, CH_2_), 4.66 (t, J = 5.60 Hz, 2H, CH_2_), 5.20 (s, 2H, CH_2_), 6.81–6.88 (m, 1H, Ar), 6.89–6.96 (m, 2H, Ar), 6.99–7.08 (m, 1H, Ar), 7.45–7.55 (m, 2H, Ar), 8.29 (s, 1H, CH), 9.00 (bs, 1H, OH), 9.30 (bs, 2H, OH). ^13^C NMR (DMSO-d_6_, 125 MHz): δ (ppm) 40.98, 47.92, 58.04, 109.62, 111.12, 118.29, 119.94, 124.23, 125.47, 126.61, 139.03, 143.22, 146.54, 151.15, 159.11, 166.42, 175.96, 183.89. ESI(+)-MS: *m*/*z* [M + H]^+^ calcd for [C_20_H_17_N_4_O_7_]^+^, 425.1092, found 425.1098. 

**(1-(2-(5-methoxy-isatin-1-yl)ethyl)-1,2,3-triazol-4-yl)methyl 3,4,5-trihydroxybenzoate (24).** Violet solid, yield = 92%. ^1^H NMR (DMSO-d_6_, 300 MHz): δ (ppm) 3.71 (s, 1H, CH_3_), 4.10 (t, J = 5.40 Hz, 2H, CH_2_), 4.64 (t, J = 5.40 Hz, 2H, CH_2_), 5.21 (s, 2H, CH_2_), 6.80–6.88 (m, 1H, Ar), 6.90–6.96 (m, 2H, Ar), 7.06–7.15 (m, 2H, Ar), 8.29 (s, 1H, CH), 9.00 (bs, 1H, OH), 9.30 (bs, 2H, OH). ^13^C NMR (DMSO-d_6_, 125 MHz): δ (ppm) 41.00, 47.87, 56.79, 58.12, 109.58, 112.32, 118.81, 119.93, 124.80, 126.50, 139.60, 143.26, 144.96, 146.56, 156.70, 159.15, 166.46, 175.97, 184.14. ESI(+)-MS: *m*/*z* [M + H]^+^ calcd for [C_21_H_19_N_4_O_8_]^+^, 455.1197, found 455.1216.

**(1-(2-(5-nitro-isatin-1-yl)ethyl)-1,2,3-triazol-4-yl)methyl 3,4,5-trihydroxybenzoate (25).** Yellow solid, yield = 89%. ^1^H NMR (DMSO-d_6_, 300 MHz): δ (ppm) 4.13–4.35 (m, 2H, CH_2_), 4.67 (s, 2H, CH_2_), 5.19–5.25 (m, 2H, CH_2_), 6.85–7.17 (m, 3H, Ar), 8.14–8.45 (m, 3H, Ar and CH), 9.22 (bs, 3H, OH). ^13^C NMR (DMSO-d_6_, 125 MHz): δ (ppm) 47.86, 58.16, 67.04, 109.52, 121.21, 126.75, 129.30, 133.80, 139.66, 143.36, 144.24, 146.58, 149.81, 155.38, 159.68, 166.46, 173.81, 181.77. ESI(+)-MS: *m*/*z* [M + H]^+^ calcd for [C_20_H_16_N_5_O_9_]^+^, 470.0943, found: 470.0936.

**(1-(2-(5-chloro-isatin-1-yl)ethyl)-1,2,3-triazol-4-yl)methyl 3,4,5-trihydroxybenzoate (26).** Red solid, yield = 88%. ^1^H NMR (DMSO-d_6_, 300 MHz): δ (ppm) 4.13 (t, J = 5.88 Hz, 2H, CH_2_), 4.64 (t, J = 5.88 Hz, 2H, CH_2_), 5.21 (s, 2H, CH_2_), 6.87–6.97 (m, 3H, Ar), 7.48–7.65 (m, 2H, Ar), 8.29 (s, 1H, CH), 9.00 (bs, 1H, OH), 9.30 (bs, 2H, OH). ^13^C NMR (DMSO-d_6_, 125 MHz): δ (ppm) 41.07, 47.83, 58.09, 109.60, 112.94, 119.69, 119.92, 125.01, 126.60, 128.56, 137.90, 139.62, 143.29, 146.57, 149.68, 158.89, 166.46, 182.81. ESI(+)-MS: *m*/*z* [M + H]^+^ calcd for [C_20_H_16_ClN_4_O_7_]^+^, 459.0702, found 459.0704.

**(1-(2-(isatin-1-yl)ethyl)-1,2,3-triazol-4-yl)methyl 4-hydroxybenzoate (27).** Orange solid, yield = 92%. ^1^H NMR (DMSO-d_6_, 300 MHz): δ (ppm) 4.13 (t, J = 6.03 Hz, 2H, CH_2_), 4.66 (t, J = 6.03 Hz, 2H, CH_2_), 5.24 (s, 2H, CH_2_), 6.78–6.89 (m, 3H, Ar), 7.03 (t, J= 7.45 Hz, 1H, Ar), 7.45–7.55 (m, 2H, Ar), 7.76 (d, J = 8.82 Hz, 2H, Ar), 8.31 (s, 1H, CH), 10.36 (bs, 1H, OH). ^13^C NMR (DMSO-d_6_, 125 MHz): δ (ppm) 41.00, 47.94, 58.19, 111.09, 116.28, 118.27, 120.86, 124.16, 125.41, 126.60, 132.51, 138.98, 143.21, 151.14, 159.07, 163.08, 166.11, 183.85. ESI(+)-MS: *m*/*z* [M + H]^+^ calcd for [C_20_H_17_N_4_O_5_]^+^, 393.1193, found 393.1196. 

**(1-(2-(5-methoxy-isatin-1-yl)ethyl)-1,2,3-triazol-4-yl)methyl 4-hydroxybenzoate (28).** Violet solid, yield = 94%. ^1^H NMR (DMSO-d_6_, 300 MHz): δ (ppm) 3.71 (s, 3H, CH_3_), 4.11 (t, J = 5.77 Hz, 2H, CH_2_), 4.65 (t, J = 5.77 Hz, 2H, CH_2_), 5.25 (s, 2H, CH_2_), 6.76–6.88 (m, 3H, Ar), 7.05–7.14 (m, 2H, Ar), 7.76 (d, J = 8.81 Hz, 2H, Ar), 8.30 (s, 1H, CH), 10.36 (bs, 1H, OH). ^13^C NMR (DMSO-d_6_, 125 MHz): δ (ppm) 41.02, 47.94, 56.75, 58.24, 110.01, 112.23, 116.28, 118.73, 120.85, 124.84, 126.54, 132.47, 143.20, 144.98, 156.62, 159.11, 163.07, 166.12, 184.09. ESI(+)-MS: *m*/*z* [M + H]^+^ calcd for [C_21_H_19_N_4_O_6_]^+^, 423.1299, found 423.1292. 

**(1-(2-(5-nitro-isatin-1-yl)ethyl)-1,2,3-triazol-4-yl)methyl 4-hydroxybenzoate (29).** Orange solid, yield = 87%. ^1^H NMR (DMSO-d_6_, 500 MHz): δ (ppm) 4.23 (t, J = 5.64 Hz, 2H, CH_2_), 4.68 (t, J = 5.64 Hz, 2H, CH_2_), 5.24 (s, 2H, CH_2_), 6.79–6.85 (m, 2H, Ar), 6.96 (d, J = 8.84 Hz, 1H, Ar), 7.69–7.75 (m, 2H, Ar), 8.19 (d, J = 2.50 Hz, 1H, Ar), 8.32 (s, 1H, CH), 8.36 (dd, J = 8.78 Hz, 2.50 Hz, 1H, Ar), 10.35 (bs, 1H, OH). ^13^C NMR (DMSO-d_6_, 125 MHz): δ (ppm) 41.48, 48.02, 58.24, 111.45, 116.26, 118.63, 120.14, 120.76, 126.72, 132.36, 133.65, 143.37, 143.89, 155.39, 159.61, 163.08, 166.07, 181.71. ESI(+)-MS: *m*/*z* [M + H]^+^ calcd for [C_20_H_16_N_5_O_7_]^+^, 438.1044, found 438.1046. 

**(1-(2-(5-chloro-isatin-1-yl)ethyl)-1,2,3-triazol-4-yl)methyl 4-hydroxybenzoate (30).** Red solid, yield = 89%. ^1^H NMR (DMSO-d_6_, 300 MHz): δ (ppm) 4.14 (t, J = 5.92 Hz, 2H, CH_2_), 4.65 (t, J = 5.92 Hz, 2H, CH_2_), 5.25 (s, 2H, CH_2_), 6.80–6.90 (m, 3H, Ar), 7.50–7.59 (m, 2H, Ar), 7.76 (d, J = 8.70 Hz, 2H, Ar), 8.31 (s, 1H, CH), 10.36 (bs, 1H, OH). ^13^C NMR (DMSO-d_6_, 125 MHz): δ (ppm) 41.13, 47.92, 58.21, 112.83, 116.32, 119.64, 120.85, 124.93, 126.63, 128.44, 132.47, 137.81, 143.25, 149.68, 158.86, 163.08, 166.13, 182.77. ESI(+)-MS: *m*/*z* [M + H]^+^ calcd for [C_20_H_16_ClN_4_O_5_]^+^, 427.0804, found 427.0806. 

**(1-(2-(isatin-1-yl)ethyl)-1,2,3-triazol-4-yl)methyl (E)-3-(3,4-dihydroxyphenyl)acrylate (31).** Orange solid, yield = 90%. ^1^H NMR (DMSO-d_6_, 500 MHz): δ (ppm) 4.13 (t, J = 5.61 Hz, 2H, CH_2_), 4.66 (t, J = 5.61 Hz, 2H, CH_2_), 5.15 (s, 2H, CH_2_), 6.23 (d, J = 15.92 Hz, 1H, CH), 6.76 (d, J = 8.15 Hz, 1H, Ar), 6.87 (d, J = 8.18 Hz, 1H, Ar), 6.95–7.17 (m, 3H, Ar), 7.40–7.64 (m, 3H, Ar and CH), 8.27 (s, 1H, CH), 9.18 (bs, 1H, OH), 9.64 (bs, 1H, OH). ^13^C NMR (DMSO-d_6_, 125 MHz): δ (ppm) 40.99, 47.89, 57.74, 111.16, 114.38, 115.74, 116.70, 118.29, 122.49, 124.20, 125.45, 126.37, 126.61, 139.08, 143.19, 146.54, 146.59, 149.50, 151.14, 159.07, 167.09, 183.86. ESI(+)-MS: *m*/*z* [M + H]^+^ calcd for [C_22_H_19_N_4_O_6_]^+^, 435.1299, found 435.1292.

**(1-(2-(5-methoxyisatin-1-yl)ethyl)-1,2,3-triazol-4-yl)methyl (E)-3-(3,4-dihydroxyphenyl)-acrylate (32).** Red solid, yield = 93%. ^1^H NMR (DMSO-d_6_, 500 MHz): δ (ppm) 3.71 (s, 3H, CH_3_), 4.10 (t, J = 5.95 Hz, 2H, CH_2_), 4.65 (t, J = 5.95 Hz, 2H, CH_2_), 5.15 (s, 2H, CH_2_), 6.23 (d, J = 15.89 Hz, 1H, CH), 6.77 (d, J = 8.32 Hz, 1H, Ar), 6.81 (d, J = 8.61 Hz, 1H, Ar), 7.00 (dd, J = 8.34 Hz, 2.07 Hz, 1H, Ar), 7.05 (d, J = 2.07 Hz, 1H, Ar), 7.10 (d, J = 2.68 Hz, 1H, Ar), 7.14 (dd, J = 8.61 Hz, 2.68 Hz, 1H, Ar), 7.47 (d, J = 15.89 Hz, 1H, CH), 8.25 (s, 1H, CH), 9.16 (bs, 1H, OH), 9.63 (bs, 1H, OH). ^13^C NMR (DMSO-d_6_, 125 MHz): δ (ppm) 41.06, 47.97, 56.83, 57.84, 110.08, 112.35, 114.38, 115.84, 116.72, 118.78, 122.55, 125.03, 126.44, 126.63, 143.24, 145.04, 146.58, 146.68, 149.55, 156.71, 159.16, 167.17, 184.16. ESI(+)-MS: *m*/*z* [M + H]^+^ calcd for [C_23_H_21_N_4_O_7_]^+^, 465.1405, found 465.1413. 

**(1-(2-(5-nitro-isatin-1-yl)ethyl)-1,2,3-triazol-4-yl)methyl (E)-3-(3,4-dihydroxyphenyl)- acrylate (33).** Orange solid, yield = 86%. ^1^H NMR (DMSO-d_6_, 300 MHz): δ (ppm) 4.23 (t, J = 5.87 Hz, 2H, CH_2_), 4.67 (t, J = 5.88 Hz, 2H, CH_2_), 5.15 (s, 2H, CH_2_), 6.20 (d, J = 15.86 Hz, 1H, CH), 6.76 (d, J = 8.16 Hz, 1H, Ar), 6.99 (dd, J = 8.40 Hz, 2.15 Hz, 1H, Ar), 7.01 (d, J = 8.85 Hz, 1H, Ar), 7.04 (d, J = 2.15 Hz, 1H, Ar), 7.45 (d, J = 15.87 Hz, 1H, CH), 8.22 (d, J = 2.40 Hz, 1H, Ar), 8.28 (s, 1H, CH), 8.41 (dd, J = 8.81 Hz, 2.45 Hz, 1H, Ar), 9.36 (bs, 2H, OH). ^13^C NMR (DMSO-d_6_, 125 MHz): δ (ppm) 41.38, 47.93, 57.80, 111.54, 114.18, 115.83, 116.64, 118.64, 120.18, 122.40, 126.32, 126.70, 133.75, 143.31, 143.95, 146.50, 146.64, 149.49, 155.36, 159.58, 167.05, 181.71. ESI(+)-MS: *m*/*z* [M + H]^+^ calcd for [C_22_H_18_N_5_O_8_]^+^, 480.1150, found 480.1145. 

**(1-(2-(5-chloro-isatin-1-yl)ethyl)-1,2,3-triazol-4-yl)methyl (E)-3-(3,4-dihydroxyphenyl) acrylate (34).** Red solid, yield = 88%. ^1^H NMR (DMSO-d_6_, 300 MHz): δ (ppm) 4.13 (t, J = 5.66 Hz, 2H, CH_2_), 4.64 (t, J = 5.65 Hz, 2H, CH_2_), 5.15 (s, 2H, CH_2_), 6.23 (d, J = 15.95 Hz, 1H, CH), 6.76 (d, J = 8.09 Hz, 1H, Ar), 6.85 (d, J = 8.27 Hz, 1H, Ar), 6.95–7.10 (m, 2H, Ar), 7.48 (d, J = 15.94 Hz, 1H, CH), 7.54–7.64 (m, 2H, Ar), 8.26 (s, 1H, CH), 9.38 (bs, 2H, OH). ^13^C NMR (DMSO-d_6_, 125 MHz): δ (ppm) 41.14, 47.94, 57.82, 112.92, 114.35, 115.84, 116.71, 119.66, 122.56, 124.98, 126.42, 126.71, 128.52, 137.94, 143.27, 146.56, 146.69, 149.54, 149.71, 158.89, 167.16, 182.82. ESI(+)-MS: *m*/*z* [M + H]^+^ calcd for [C_22_H_18_ClN_4_O_6_]^+^, 469.0909, found 469.0900. 

### 3.2. Molecular Docking Calculations

Geometry optimizations and frequency calculations for stationary point characterization were carried out with Gaussian16 using the M06–2X hybrid functional [69], the 6–31 G (d, p) basis set, and ultrafine integration grids. Bulk solvent effects in water were considered implicitly through the IEF-PCM polarizable continuum model [70]. The protein structure was processed with AutodockTools-1.5.6rc3 [71] to toggle problems of incomplete structures due to missing atoms or water molecules. Ligands and protein structure files were converted to PDBQT (Protein Data Bank Partial Charge and Atom Type) and the docking was executed using the Lamarckian algorithm. The grid box consisted of 30 grid points in all three dimensions (X, Y, and Z) separated by a distance of 1 Å between each one. The crystallographic ligands were docked after adding hydrogen atoms with AutodockTools-1.5.6rc3 and without optimizing their geometry. For all the simulations, the protein was kept rigid allowing for flexibilization of the ligands around their rotatable bonds.

## 4. Conclusions

In conclusion, in this work, we designed a new library of hybrids containing a 1,2,3-triazole moiety as a linker between an isatin and a phenolic acid. Firstly, four different 1-(2-azidoethyl)isatines and three phenolic acid propargyl esters were prepared in good to excellent yields. Then, after the optimization of the reaction condition, a CuAAC was performed to obtain twelve different 1,4-disubstituted-1,2,3-triazoles, in a highly regioselective manner and in excellent yields (86–92%). The influence of the substituents on the aromatic rings was evaluated and all the hybrids were purified and characterized. Finally, a molecular docking study was carried out to hypothesize potential therapeutic targets for these new compounds. Eight different potential enzymatic substrates were found considering the previous affinities reported in the literature for the three building blocks (isatine, 1,2,3-triazole, and phenolic acid) as a research parameter. All the hybrids showed strong potential affinity with them, if compared to the reference ligands reported in the literature. The most interesting results, in term of affinity, were found for all the hybrids on the enzyme 5-lipoxygenase (5-LOX), in the presence of its natural substrate, the arachidonic acid (ACD), thus confirming the possibility to potentially develop a new class of anti-inflammatory agents starting from these compounds.

## Figures and Tables

**Table 1:** Synthesis of propargyl esters **20**–**22** starting from phenolic acids **14**–**16**.

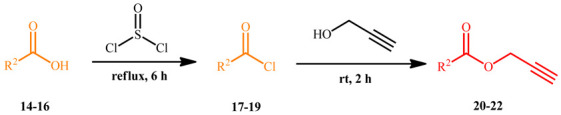					
Entry	R^2^	Phenolic Acid	Acyl Chloride	Propargyl Ester	Yield (%)
1	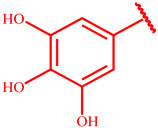	**14**	**17**	**20**	98
2	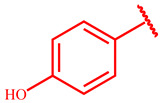	**15**	**18**	**21**	94
3	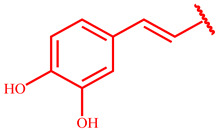	**16**	**19**	**22**	92

**Table 2:** Optimization of reaction conditions.

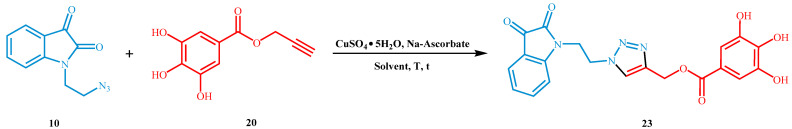							
Entry ^a^	Solvent (*v*/*v*)	Reagent 10 (eq)	Catalytic System (eq)		T (°C)	t (h)	Yield ^b^ (%)
			CuSO_4_ 5H_2_O	Na-Ascorbate			
1	DMF/H_2_O (1:1)	1.3	0.1	0.4	rt	48	22
2	DMF/H_2_O (1:1)	1.3	0.1	0.4	50	5	61
3	DMF/H_2_O (1:1)	1.3	0.1	0.4	60	5	67
4	EtOH/H_2_O (1:1)	1.3	0.1	0.4	60	5	45
5	*^i-^*PrOH/H_2_O (1:1)	1.3	0.1	0.4	60	5	38
6	*^t-^*BuOH/H_2_O (1:1)	1.3	0.1	0.4	60	5	43
7	DMF	1.3	0.1	0.4	60	5	72
8 ^c^	DMF	1.3	0.2	0.8	60	5	90
9 ^d^	DMF	1.0	0.2	0.8	60	5	91 ^e^
10	DMF	1.0	-	-	60	5	traces
11 ^f^	DMF	1.0	0.2	0.8	60	5	92 ^e^
12	[mPy]OTf	1.0	0.2	0.8	60	5	traces

^a^ Reaction conditions: azide **10** (1.56 mmol), alkyne **20** (1 eq, 1.20 mmol), CuSO_4_ 5H_2_O (0.12 mmol), Na-Ascorbate (0.48 mmol) in solvent (20 mL). ^b^ Isolated Yield. ^c^ Reaction conditions: azide **10** (1.56 mmol), alkyne **20** (1 eq, 1.20 mmol), CuSO_4_ 5H_2_O (0.24 mmol), Na-Ascorbate (0.96 mmol) in DMF (20 mL). ^d^ Reaction conditions: azide **10** (1.20 mmol), alkyne **20** (1 eq, 1.20 mmol), CuSO_4_ 5H_2_O (0.24 mmol), Na-Ascorbate (0.96 mmol) in DMF (20 mL). ^e^ See Supplementary Materials for ^1^H NMR of reaction crude. ^f^ Gram-scale reaction conditions: azide **10** (4.80 mmol), alkyne **20** (1 eq, 4.80 mmol), CuSO_4_ 5H_2_O (0.96 mmol), Na-Ascorbate (3.84 mmol) in DMF (80 mL).

**Table 3:** Synthesis of new hybrid molecules based on 1,4-disubstituted 1,2,3-triazoles by CuAAC.

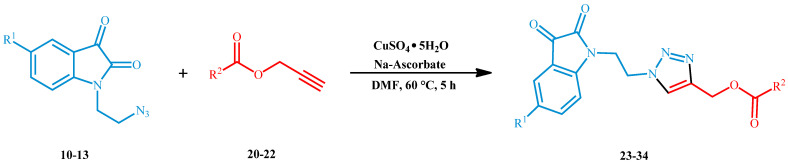				
Entry ^a^	R ^1^	R ^2^	Product	Yield ^b^ (%)
1	H	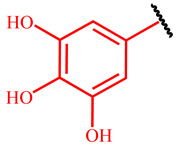	**23**	91
2	MeO		**24**	92
3	NO_2_		**25**	89
4	Cl		**26**	88
5	H	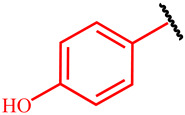	**27**	92
6	MeO		**28**	94
7	NO_2_		**29**	87
8	Cl		**30**	89
9	H	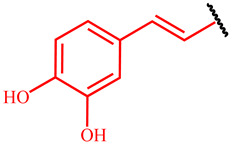	**31**	90
10	MeO		**32**	93
11	NO_2_		**33**	86
12	Cl		**34**	88

^a^ Reaction conditions: azide **10** (1.20 mmol), alkyne **20** (1.20 mmol), CuSO_4_ · 5H_2_O (0.24 mmol), Na-Ascorbate (0.96 mmol) in DMF (5 mL). ^b^ Isolated Yield.

**Table 4:** Best docking affinity values of compounds **23**–**34** vs. co-crystalized ligands on selected receptors.

Receptor	Best Scores Affinity (kcal/mol)												
	Reference Ligand	23	24	25	26	27	28	29	30	31	32	33	34
5-Lipoxygenase **5-LOX** (PDB: 308Y)	**DHC** −6.1	**−7.1**	−5.0	−5.1	−6.1	**−7.1**	−6.4	−5.8	−6.1	−6.0	−4.0	−4.0	−4.8
5-Lipoxygenase with ACD **5-LOX** (PDB: 3V99)	**DHC** −6.5	−8.7	−8.7	−8.7	**−9.0**	−8.8	−8.7	**−9.0**	**−9.0**	−8.2	−8.1	−7.6	−8.7
Cyclooxygenase-2 **COX-2** (PDB: 4FM5)	**DF0** −8.8	−9.6	−9.6	−9.8	−9.8	−9.6	−9.6	−9.8	−9.9	**−10.3**	−10.1	−10.2	−10.1
Angiotensin-converting enzyme **ACE** (PDB: 1O86)	**LPR** −7	−9.6	−9.4	−9.0	**−9.8**	−9.3	−9.0	−9.0	−9.4	−9.6	−8.9	−8.6	−9.5
Human topoisomerase II ATPase **TOP2** (PDB: 1ZXM)	**ANP** −10.9	−10.4	−10.5	−10.0	−10.3	−10.0	−10.0	**−10.7**	−9.7	−10.5	−10.4	−10.8	−10.0
Tubulin (PDB: 1SA0)	**CN2** −8.5	−9.4	−9.5	−9.6	**−9.7**	−9.0	−8.9	−9.3	−8.9	−9.1	−8.8	−8.9	−8.9
Acetyilcholinesterase **AChE** (PDB: 1EVE)	**E20** −11.0	−10.7	−10.8	−11.0	−11.0	−10.7	−10.3	−11.3	−10.8	−11.5	−11.6	**−12.0**	−11.6
D-Alanyl-D-alanine ligase **Ddl** (PDB: 2ZDQ)	**ATP** −9.7	−10.0	**−10.8**	−10.1	−10.1	−9.7	−9.7	−10.3	−10.0	−10.2	−10.0	−10.4	−10.0

**Table 5:** Percentage difference between the average affinity values of the triazole conjugates vs. the reference ligand.

Target/Ligand	Literature Ligand	Reference Affinity Value (kcal/mol)	Average Affinity Value of All Triazole Conjugates (kcal/mol)	Difference
5-LOX	DHC	−6.1	−5.9	−3.7%
5-LOX with ACD	DHC	−6.5	−8.6	**+32.3%**
COX-2	DF0	−8.8	−9.9	**+12.2%**
ACE	LPR	−7.0	−9.3	**+32.1**
TOP2	ANP	−10.9	−10.3	−5.7%
Tubulin	CN2	−8.5	−9.2	**+7.9%**
AChE	E20	−11.0	−11.1	+1.0%
DdI	ATP	−9.7	−10.1	+4.2%

## Data Availability

The data presented in this study are available in article and Supplementary Materials.

## Supplementary Materials

The following supporting information can be downloaded at: https://www.mdpi.com/article/10.3390/molecules29071556/s1. Characterization spectra of the 1-(2-bromoethyl) isatins **6**–**9** (^1^H NMR; ^13^C NMR; COSY NMR; HRMS); Characterization spectra of the 1-(2-azidoethyl) isatins **10**–**13** (^1^H NMR; ^13^C NMR; COSY NMR; HRMS); Characterization spectra of the propargyl esters **20**–**22** (^1^H NMR; ^13^C NMR; COSY NMR; HRMS); ^1^H NMR (300 MHz) spectra of crude of the model reaction for synthesis of **23**; Characterization spectra of the hybrid molecules **23**–**34** (^1^H NMR; ^13^C NMR; COSY NMR; HRMS).
