# Supplementary material for: 1,2,3-Triazole Hybrids Containing Isatins and Phenolic Moieties: Regioselective Synthesis and Molecular Docking Studies
Source: Molecules. 2024 Mar 30;29(7):1556. doi: 10.3390/molecules29071556 (PMC11013233; doi:10.3390/molecules29071556)
Supplement: Supplementary file 1 [file molecules-29-01556-s001.zip › molecules-2938910-supplementary.pdf]

SUPPLEMENTARY MATERIALS

1,2,3-Triazole hybrids containing isatins and phenolic moieties: regioselective synthesis and molecular docking studies

Loredana Maiuolo[1], Matteo Antonio Tallarida[2], Angelo Meduri,[3] Giulia Fiorani[4], Antonio Jiritano[1], Antonio De Nino[1], Vincenzo Algieri [5,]* Paola Costanzo[1],*

[1] Department of Chemistry and Chemical Technologies, University of Calabria, 87036 Rende, Italy; maiuolo@unical.it; antonio.jiritano@unical.it; denino@unical.it
[2] RINA Consulting - Centro Sviluppo Materiali SpA, Via di Castel Romano 100, Rome, 00128, Italy; matteo.tallarida@rina.org
[3] RINA Consulting – Centro Sviluppo Materiali SpA, Zona Industriale San Pietro Lametino, comparto 1, 88046 Lamezia Terme, CZ, Italy; angelo.meduri@rina.org
[4] Department Molecular Sciences and Nanosystems, University Ca' Foscari Venezia, 30172 Mestre, VE, Italy; giulia.fiorani@unive.it
[5] IRCCS NEUROMED – Istituto Neurologico Mediterraneo, Via Atinense 18, 86077 – Pozzilli, IS, IT.
* Correspondence: vincenzo.algieri@unical.it (VA); paola.costanzo@unical.it (PC

General procedure for synthesis of different 1-(2-bromoethyl)isatins 6-9……………………..…S1

General procedure for synthesis of variously 1-(2-azidoethyl)isatins 10-13…………………...S3

General procedure for synthesis of propargyl esters 20-22…………………………………......S5

Characterization spectra of 1-(2-bromoethyl) isatins 6-9……………………….....................S6

Characterization spectra of 1-(2-azidoethyl) isatins 10-13……………………………………S21

Characterization spectra of propargyl esters 20-22…………………………………………...S37

^{1}H NMR (300 MHz) spectra of crude of the model reaction for synthesis of 23…………….S49

Characterization spectra of hybrid molecules 23-34…………………………………………S50

General procedure for synthesis of 1-(2-bromoethyl)isatins 6-9:

In 250 ml three-necked round-bottomed flask, equipped with bubble condenser and magnetic stir bar, opportune isatin **1-4** (3.40×10^{-2} mol) was dissolved in *dry* DMF (30 ml) and anhydrous K_2CO_3 (7.05 g, 5.1×10^{-2} mol) was added. The resulting suspension was stirred at room temperature for 20 min before the addition of freshly distilled 1,2-dibromoethane **5** (29.5 ml, 63.85 g, 3.40×10^{-1} mol). The reaction mixture was heated to 40 °C with constant stirring for about 5 h. After the completion of reaction, water (20 ml) was added and crude was extracted with ethyl acetate (3 x 30 ml). The combined organic layers were washed with brine solution, dried over anhydrous Na_2SO_4, filtered and concentrated in vacuo. Purification of reaction crude by flash chromatography by using hexane:ethyl acetate (8:2 v/v) furnished the desired 1-(2-bromoethyl)isatin **6-9** in good yields.

1-(2-Bromoethyl)isatin (6)

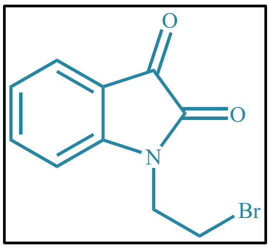

^{1}H NMR (DMSO-d_6, 300 MHz): δ (ppm) 3.72 (t, J=6.73 Hz, 2H, CH_2), 4.12 (t, J=6.73 Hz, 2H, CH_2), 7.15 (t, J=7.55 Hz, 1H, Ar), 7.30 (d, J=7.98 Hz, 1H, Ar), 7.57 (d, J=7.45 Hz, 1H, Ar), 7.68 (t, J=7.51 Hz, 1H, Ar). ^{13}C NMR (DMSO-d_6, 125 MHz): δ (ppm) 30.08, 42.12, 112.02, 118.32, 124.36, 125.53, 139.24, 151.13, 159.06, 183.91. ESI(+)-MS: *m/z* $[M+H]^+$ calcd for $[C_{10}H_9BrNO_2]^+$, 253.9811, found 253.9798.

1-(2-Bromoethyl)-5-methylisatin (7)

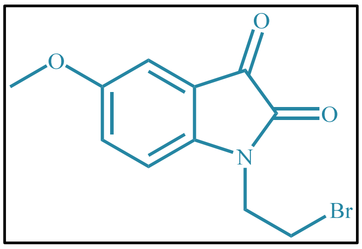

^{1}H NMR (DMSO-d_6, 300 MHz): δ (ppm) 3.70 (t, J=6.48 Hz, 2H, CH_2), 3.77 (s, 3H, CH_3), 4.09 (t, J=6.48 Hz, 2H, CH_2), 7.12-7.17 (m, 1H, Ar), 7.22-7.27 (m, 2H, Ar).^{13}C NMR (DMSO-d_6, 125 MHz): δ (ppm) 30.24, 42.19, 56.92, 110.24, 113.20, 118.88, 125.12, 145.02, 156.83, 159.19, 184.24.ESI(+)-MS: *m/z* $[M+H]^+$ calcd for $[C_{11}H_{11}BrNO_3]^+$, 283.9917, found 284.0002.

1-(2-Bromoethyl)-5-nitroisatin (8)

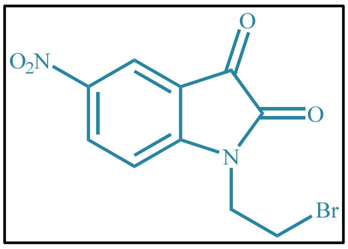

^{1}H NMR (DMSO-d_6, 300 MHz): δ (ppm) 3.76 (t, J=6.12 Hz, 2H, CH_2), 4.16 (t, J=6.12 Hz, 2H, CH_2), 7.46 (d, J= 8.80 Hz, 1H, Ar), 8.24 (d, J= 2.35 Hz, 1H, Ar), 8.37 (dd, J=8.78 Hz, 2.47 Hz, 1H, Ar). ^{13}C NMR (DMSO-d_6, 125 MHz): δ (ppm) 30.29, 42.26, 101.26, 111.62, 121.23, 126.14, 129.40, 144.33, 149.90, 173.99. ESI(+)-MS: *m/z* $[M+H]^+$ calcd for $[C_{10}H_8BrN_2O_4]^+$, 298.9662, found 298.9657.

1-(2-Bromoethyl)-5-chloroisatin (9)

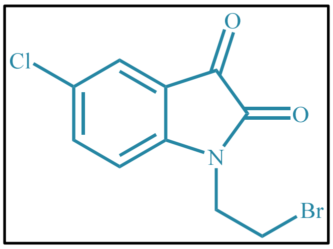

^{1}H NMR (DMSO-d_6, 300 MHz): δ (ppm) 3.69 (t, J=6.58 Hz, 2H, CH_2), 4.11 (t, J=6.58 Hz, 2H, CH_2), 7.36 (d, J= 8.51 Hz, 1H, Ar), 7.64 (d, J= 2.21 Hz, 1H, Ar), 7.73 (dd, J=8.54 Hz, 2.25 Hz, 1H, Ar). ^{13}C NMR (DMSO-d_6, 125 MHz): δ (ppm) 29.96, 42.30, 113.86, 119.78, 125.05, 128.63, 138.09, 149.74, 158.89, 182.87.

General procedure for synthesis of 1-(2-azidoethyl)isatins 10-13:

In 250 ml three-necked round-bottomed flask, equipped with bubble condenser and magnetic stir bar, opportune 1-(2-bromoethyl)isatin **6-9** (2.58 x 10^{-2} mol) in *dry* DMF (30 ml) was dissolved and NaN_3 (3.87 x 10^{-2} mol) was added. The reaction mixture was heated at 60 °C for 3 h under stirring and was monitored by TLC. Upon completion, water was added (50 ml) and the reaction mixture was extracted with ethyl acetate (3 x 30 ml). The combined organic layers were washed with brine, dried over anhydrous Na_2SO_4, filtered and concentrated under reduced pressure. The crude was purified by flash chromatography using hexane:ethyl acetate (8:2 v/v) to furnish the desired 1-(2-azidoethyl)isatin **10-13** in good yields.

1-(2-azidoethyl)isatin (10)

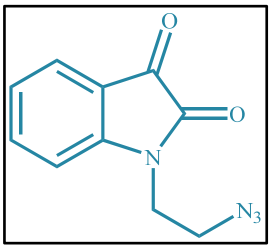

^{1}H-NMR (DMSO-*d*$_6$, 300 MHz): δ (ppm) 3.63 (t, J=5.68 Hz, 2H, CH_2), 3.89 (t, J=5.68 Hz, 2H, CH_2), 7.15 (t, J=7.54 Hz, 1H, Ar), 7.28 (d, J=7.95 Hz, 1H, Ar), 7.56 (d, J=7.54 Hz, 1H, Ar), 7.68 (t, J=7.74 Hz, 1H, Ar). ^{13}C-NMR (DMSO-*d*$_6$, 125 MHz): δ (ppm) 39.93, 48.93, 111.91, 118.36, 124.30, 125.48, 139.22, 151.37, 159.25, 183.98. ESI(+)-MS: *m/z* $[M+H]^+$ calcd for $[C_{10}H_9N_4O_2]^+$, 217.0720, found 217.0720.

1-(2-azidoethyl)-5-methylisatin (11)

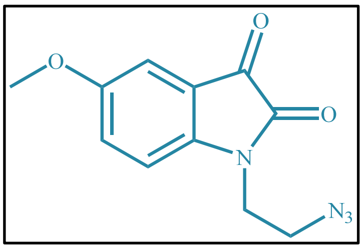

^{1}H NMR (DMSO-*d*$_6$, 300 MHz): δ (ppm) 3.61 (t, J=5.83 Hz, 2H, CH_2), 3.77 (s, 3H, CH_3), 3.86 (t, J=5.83 Hz, 2H, CH_2), 7.12-7.16 (m, 1H, Ar), 7.18-7.31 (m, 2H, Ar). ^{13}C NMR (DMSO-*d*$_6$, 125 MHz): δ (ppm) 40.00, 49.00, 56.89, 110.18, 113.09, 118.89, 125.11, 145.22, 156.80, 159.35, 184.29. ESI(+)-MS: *m/z* $[M+H]^+$ calcd for $[C_{11}H_{11}N_4O_3]^+$, 247.0826, found 247.0824.

1-(2-azidoethyl)-5-nitroisatin (12)

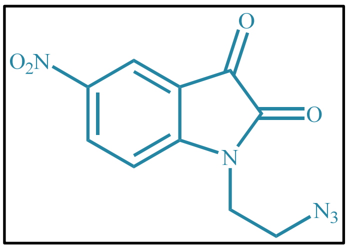

^{1}H NMR (DMSO-*d*$_6$, 300 MHz): δ (ppm) 3.63 (t, J=5.55 Hz, 2H, CH_2), 3.93 (t, J=5.55 Hz, 2H, CH_2), 7.45 (d, J= 8.81 Hz, 1H, Ar), 8.24 (d, J= 2.42 Hz, 1H, Ar), 8.39 (dd, J=8.76 Hz, 2.36 Hz, 1H, Ar). ^{13}C NMR (DMSO-*d*$_6$, 125 MHz): δ (ppm) 48.92, 67.15, 101.26, 111.46, 121.21, 126.17, 129.48, 144.31, 150.05, 174.08. ESI(+)-MS: *m/z* $[M+H]^+$ calcd for $[C_{10}H_8N_5O_4]^+$, 262.0571, found 262.0620.

1-(2-Azidoethyl)-5-chloroisatin (13)

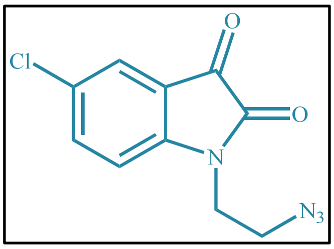

^{1}H NMR (DMSO-d_6, 300 MHz): δ (ppm) 3.61 (t, J=5.74 Hz, 2H, CH_2), 3.88 (t, J=5.74 Hz, 2H, CH_2), 7.33 (d, J= 8.48 Hz, 1H, Ar), 7.62 (d, J= 2.24 Hz, 1H, Ar), 7.73 (dd, J=8.50 Hz, 2.26 Hz, 1H, Ar). ^{13}C NMR (DMSO-d_6, 125 MHz): δ (ppm) 40.10, 49.07, 113.77, 119.81, 125.00, 128.57, 138.10, 150.02, 159.07, 182.97. ESI(+)-MS: *m/z* $[M+H]^+$ calcd for $[C_{10}H_8ClN_4O_2]^+$, 251.0330, found 251.0378.

General procedure for synthesis of propargyl esters 20-22:

In 250 ml three-necked round-bottomed flask, equipped with bubble condenser and magnetic stir bar, opportune carboxylic acid **14-16** (5.88 x 10^{-2} mol) was placed and freshly distilled thionyl chloride was added (30 ml, 4.11 x 10^{-1} mol). The reaction was conducted at reflux for 6h and subsequently the thionyl chloride in excess was removed under vacuum. The oily residue corresponding to acyl chloride **17-19**, was cooled by using an ice bath and then, propargyl alcohol (41 ml, 7.06 x 10^{-1} mol) was added dropwise for 30 min. The mixture was stirred at room temperature for 2h and then was concentrated in vacuo. Purification of crude product by flash chromatography using $CHCl_3$:MeOH (9:1 v/v) furnished the desired propargyl ester derivatives **20-22** in good yields.

Propargyl 3,4,5-trihydroxybenzoate (20)

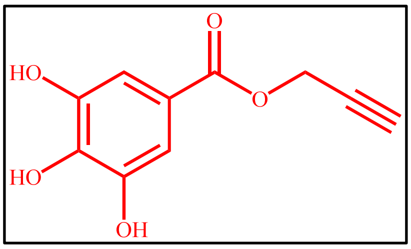

^{1}H NMR (DMSO-d_6, 300 MHz): δ (ppm) 3.57 (t, J=2.43 Hz, 1H, CH), 4.84 (d, J=2.43 Hz, 2H, CH_2), 6.95 (s, 2H, Ar), 9.27 (bs, 3H, OH). ^{13}C NMR (DMSO-d_6, 125 MHz): δ (ppm) 52.88, 78.58, 79.82, 109.66, 119.56, 139.87, 146.69, 166.07. ESI(+)-MS: *m/z* $[M+H]^+$ calcd for $[C_{10}H_9O_5]^+$, 209.0444, found 209.0453.

Propargyl 4-hydroxybenzoate (21)

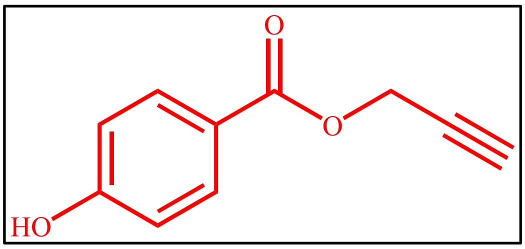

^{1}H NMR (DMSO-d_6, 300 MHz): δ (ppm) 3.58 (t, J=2.42 Hz, 1H, CH), 4.88 (d, J=2.42 Hz, 2H, CH_2), 6.81-6.90 (m, 2H, Ar), 7.78-7.87 (m, 2H, Ar), 10.46 (bs, 1H, OH). ^{13}C NMR (DMSO-d_6, 125 MHz): δ (ppm) 52.86, 78.58, 79.71, 116.47, 120.52, 132.62, 163.31, 165.77. ESI(+)-MS: *m/z* $[M+H]^+$ calcd for $[C_{10}H_9O_3]^+$, 177.0546, found 177.0546.

Propargyl (*E*)-3-(3,4-dihydroxyphenyl)acrylate (22)

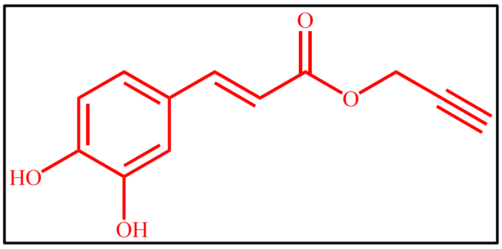

^{1}H NMR (DMSO-d_6, 300 MHz): δ (ppm) 3.55 (t, J=2.41 Hz, 1H, C≡C-H), 4.79 (d, J=2.42 Hz, 2H, C≡C-CH_2), 6.31 (d, J=15.91 Hz, 1H, O=C-C-H), 6.77 (d, J=8.10 Hz, 1H, Ar), 6.99-7.11 (m, 2H, Ar), 7.53 (d, J=15.91 Hz, 1H, OC-C=C-H), 9.46 (bs, 2H, OH). ^{13}C NMR (DMSO-d_6, 125 MHz): δ (ppm) 52.65, 78.57, 79.94, 114.14, 115.92, 116.96, 123.00, 126.54, 146.76, 147.35, 149.85, 167.03. ESI(+)-MS: *m/z* $[M+H]^+$ calcd for $[C_{12}H_{11}O_4]^+$, 219.0652, found 219.0650.

Characterization spectra of 1-(2-bromoethyl) isatins 6-9.

1-(2-Bromoethyl)isatin (6)

^{1}H NMR (300 MHz)

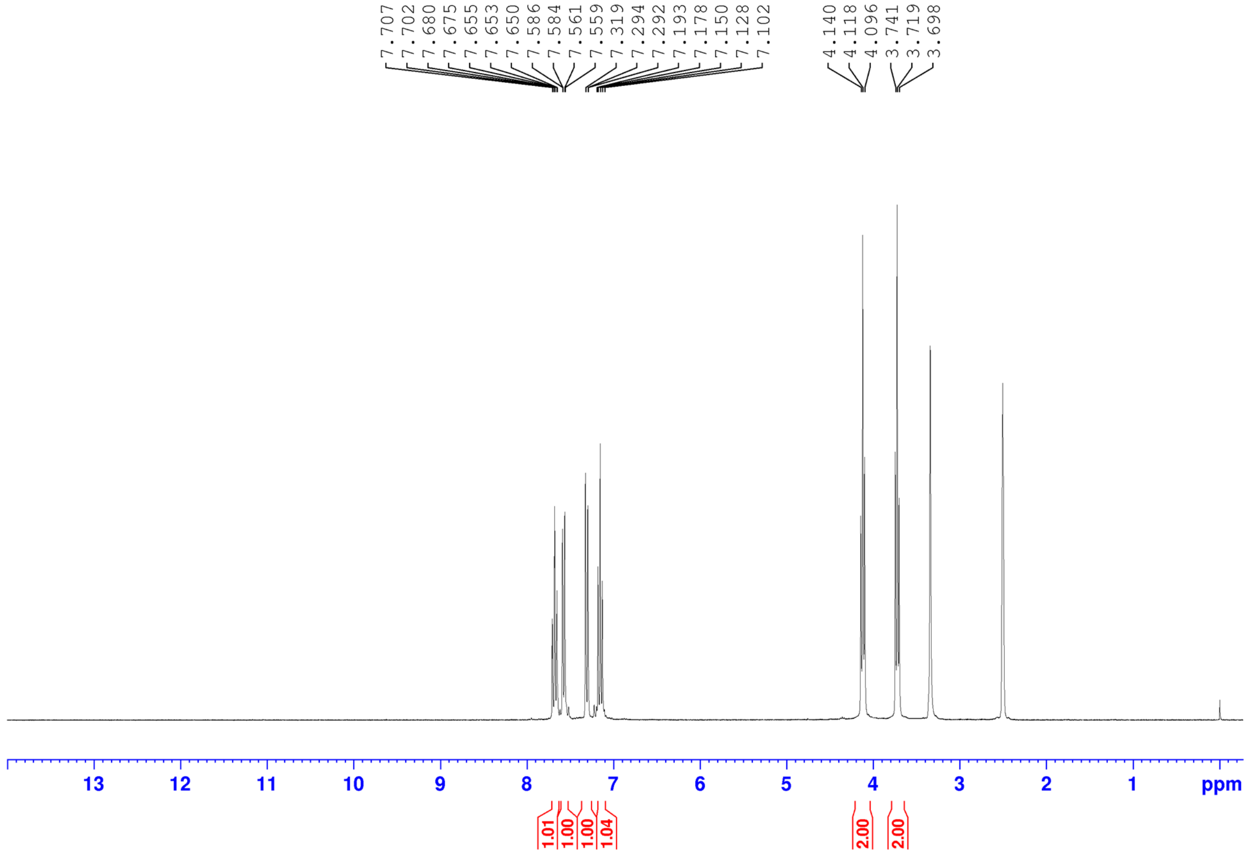

^{13}C NMR (500 MHz)

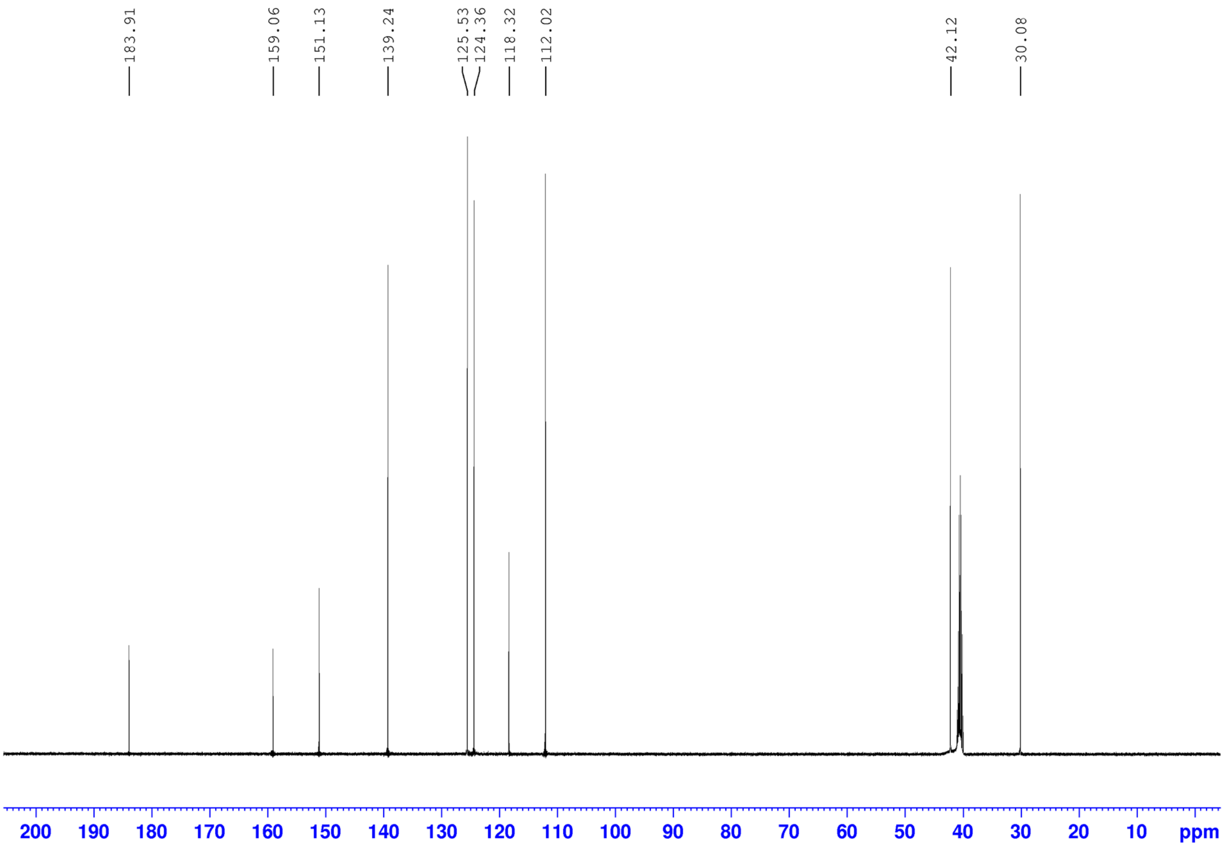

COSY NMR (300 MHz)

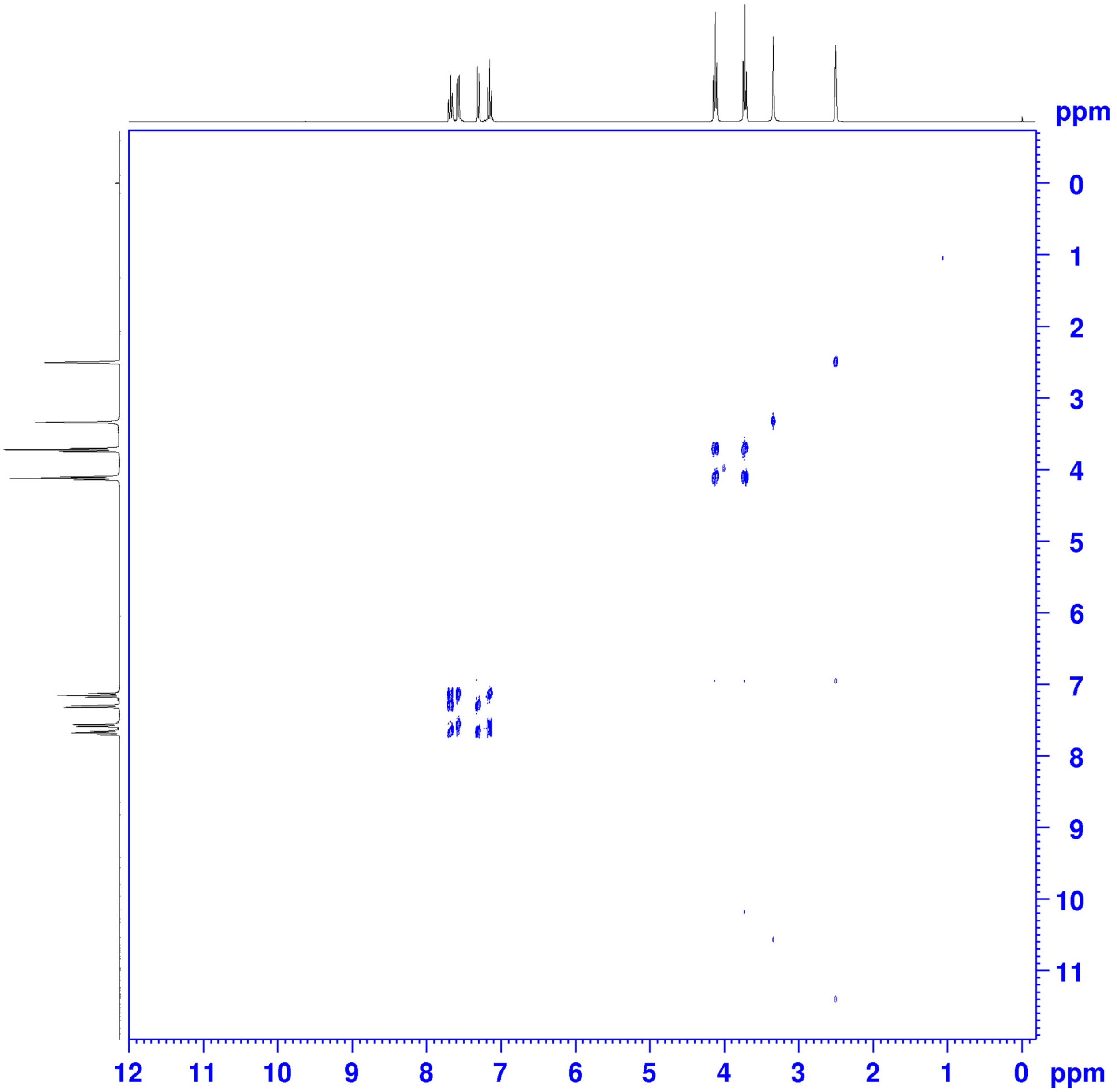

HRMS

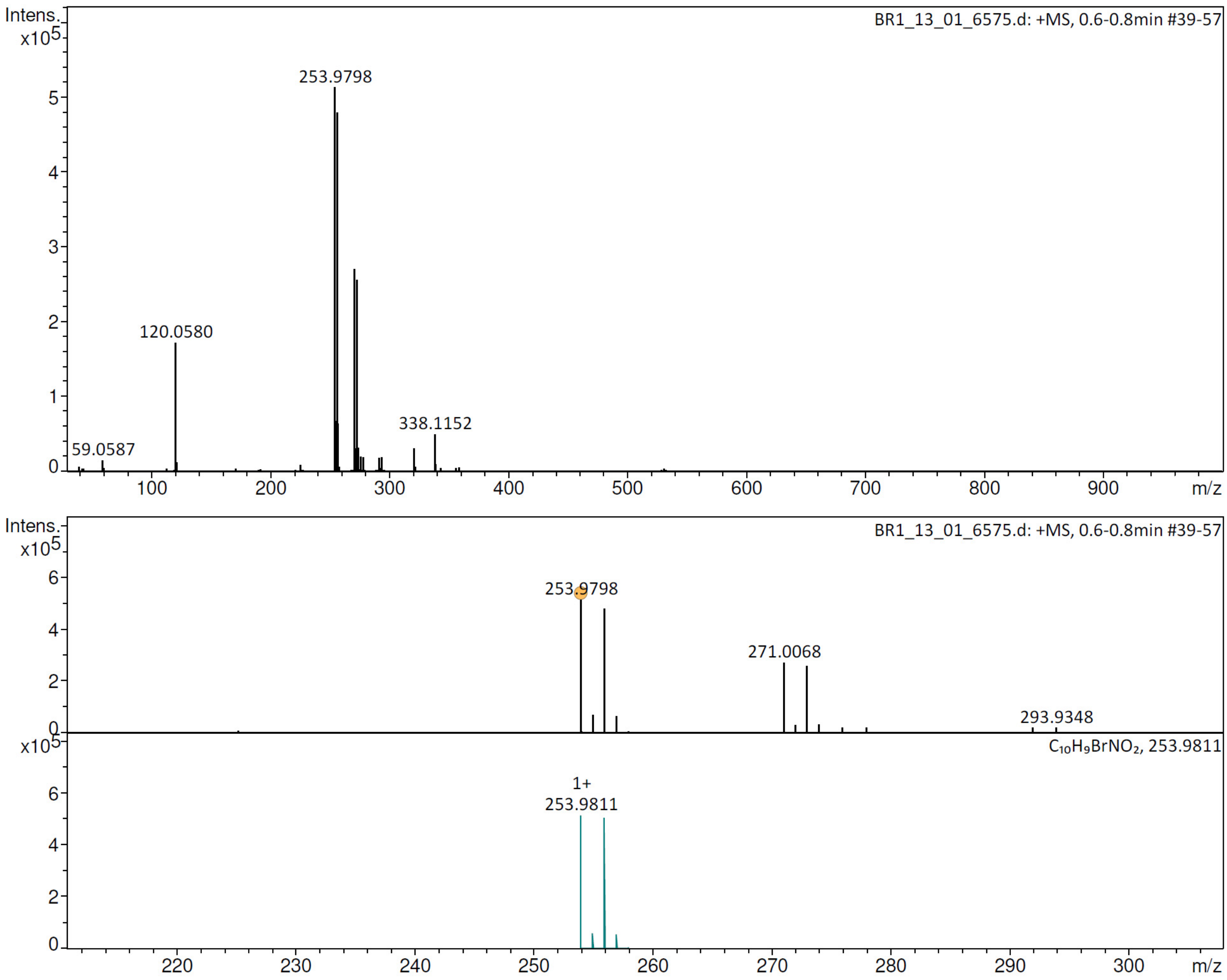
Intens.
x10^5
BR1_13_01_6575.d: +MS, 0.6-0.8min #39-57
253.9798
120.0580
338.1152
59.0587
m/z
Intens.
x10^5
BR1_13_01_6575.d: +MS, 0.6-0.8min #39-57
253.9798
271.0068
293.9348
x10^5
C10H9BrNO2, 253.9811
1+
253.9811
m/z

1-(2-Bromoethyl)-5-methylisatin (7)

^{1}H NMR (300 MHz)

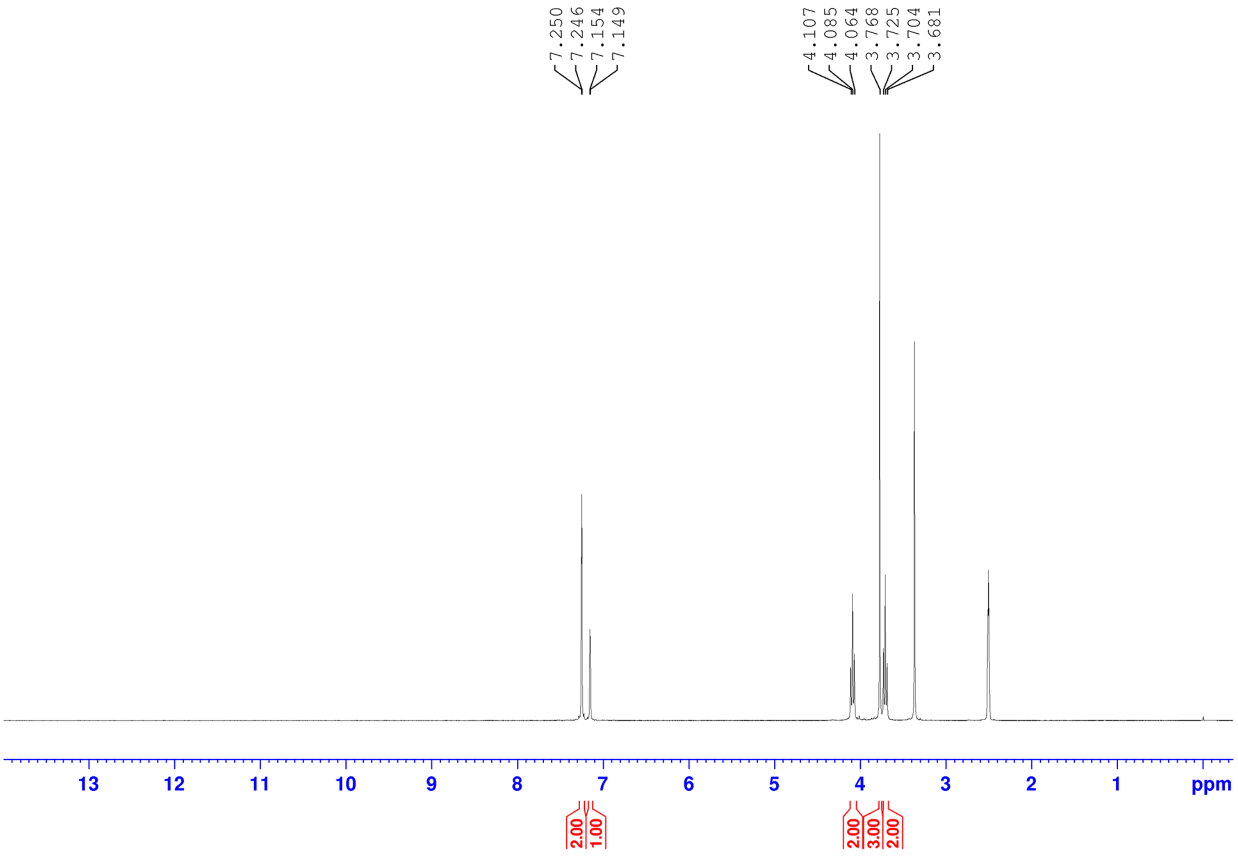

^{13}C NMR (125 MHz)

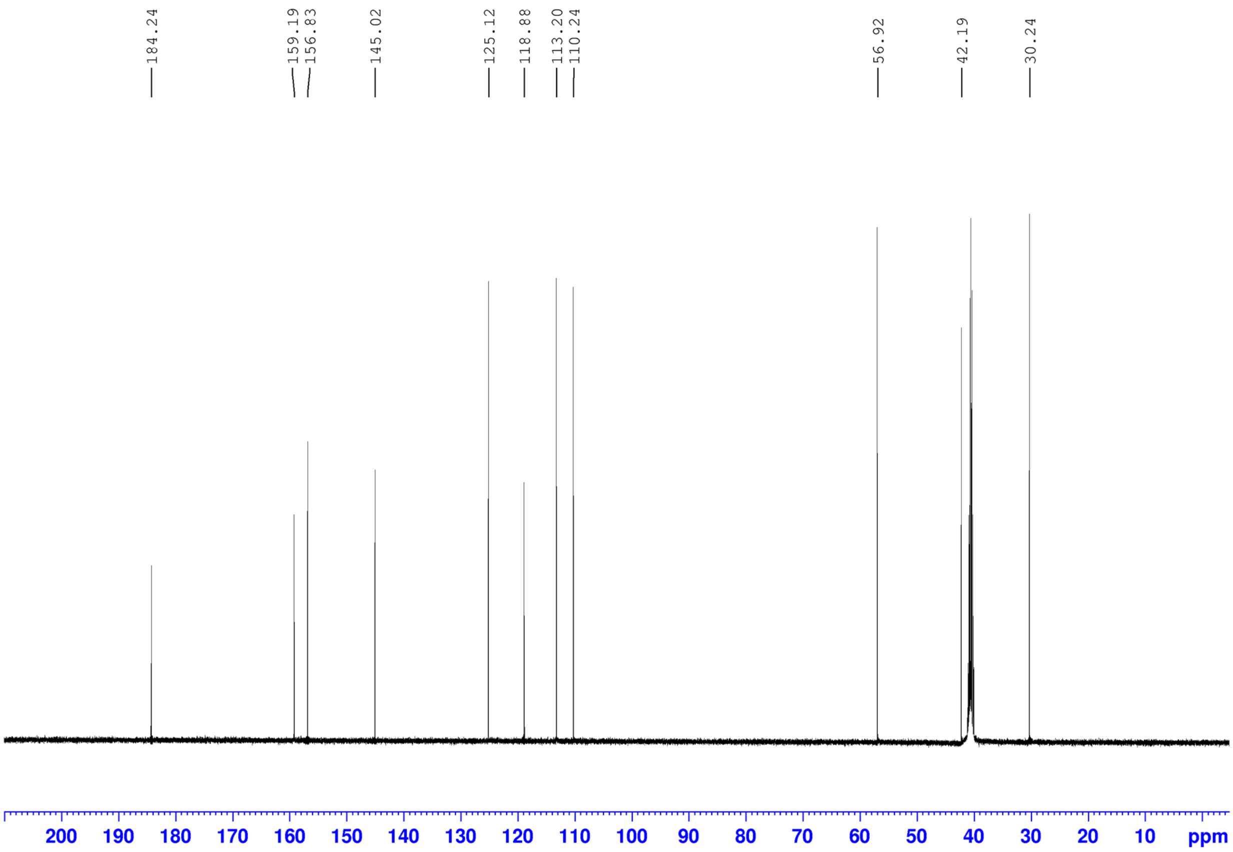

COSY NMR (300 MHz)

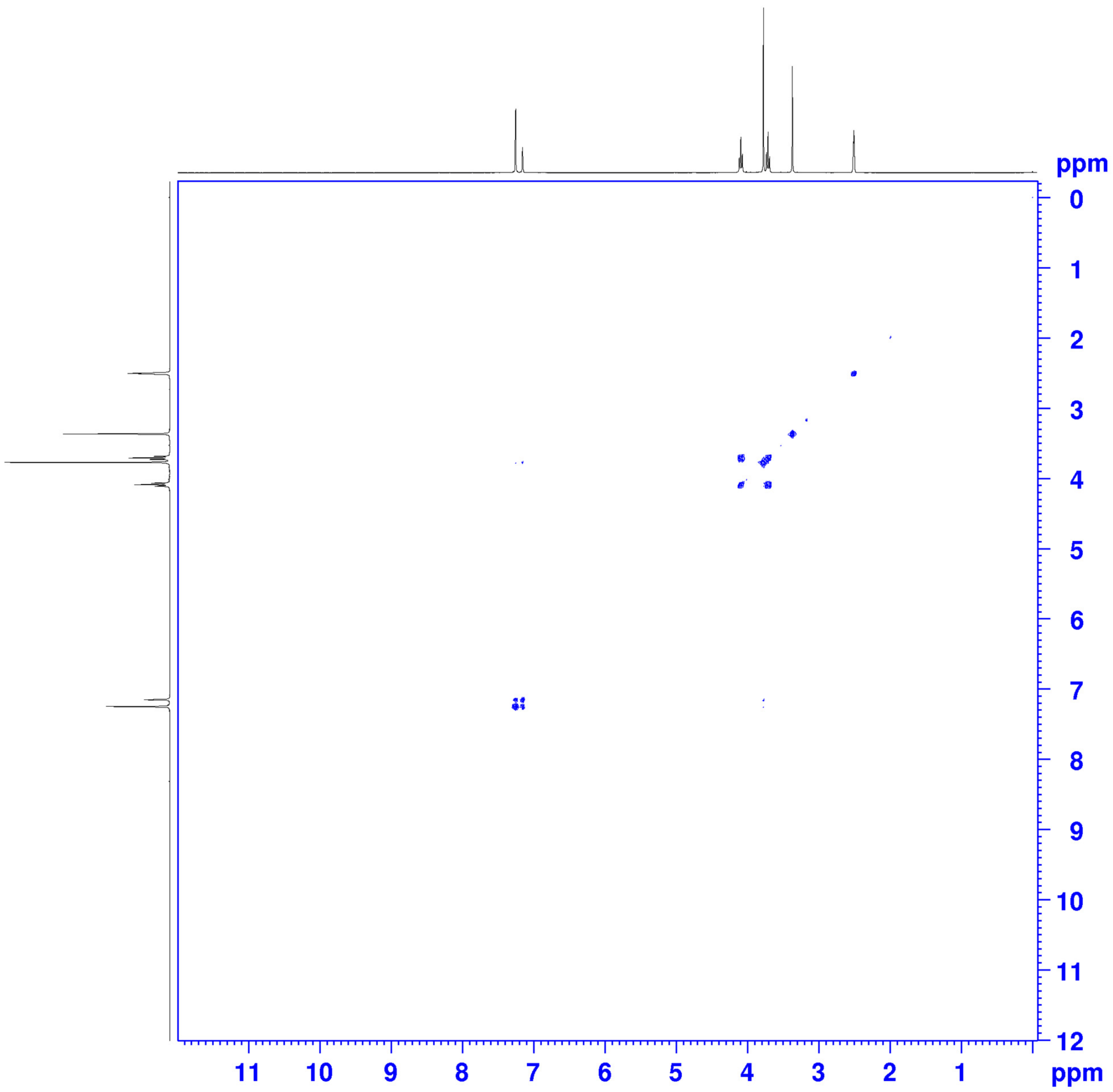

HRMS

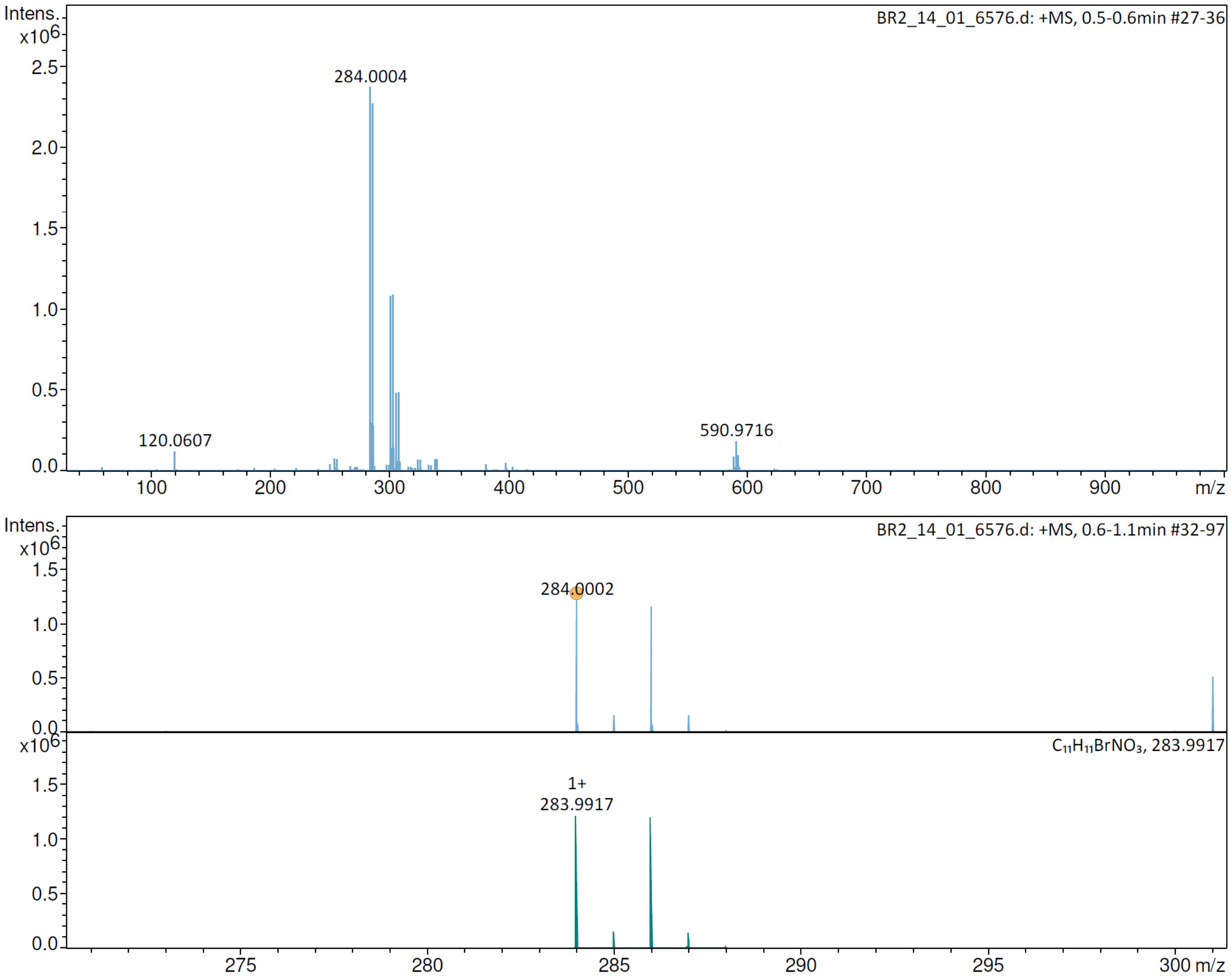

Intens.
x10^6
BR2_14_01_6576.d: +MS, 0.5-0.6min #27-36
284.0004
120.0607
590.9716
m/z
Intens.
x10^6
BR2_14_01_6576.d: +MS, 0.6-1.1min #32-97
284.0002
C11H11BrNO3, 283.9917
1+
283.9917
300 m/z

1-(2-Bromoethyl)-5-nitroisatin (8)

^{1}H NMR (300 MHz)

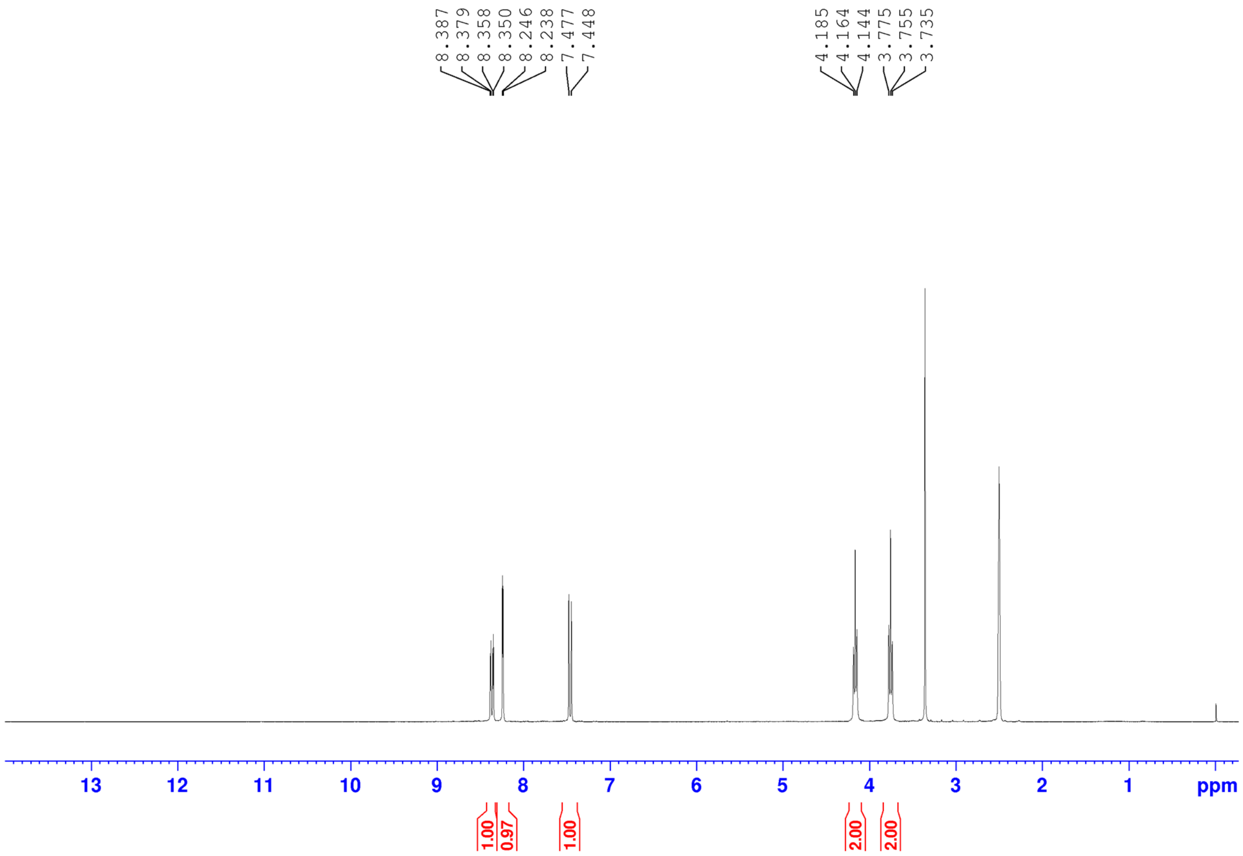

^{13}C NMR (125 MHz)

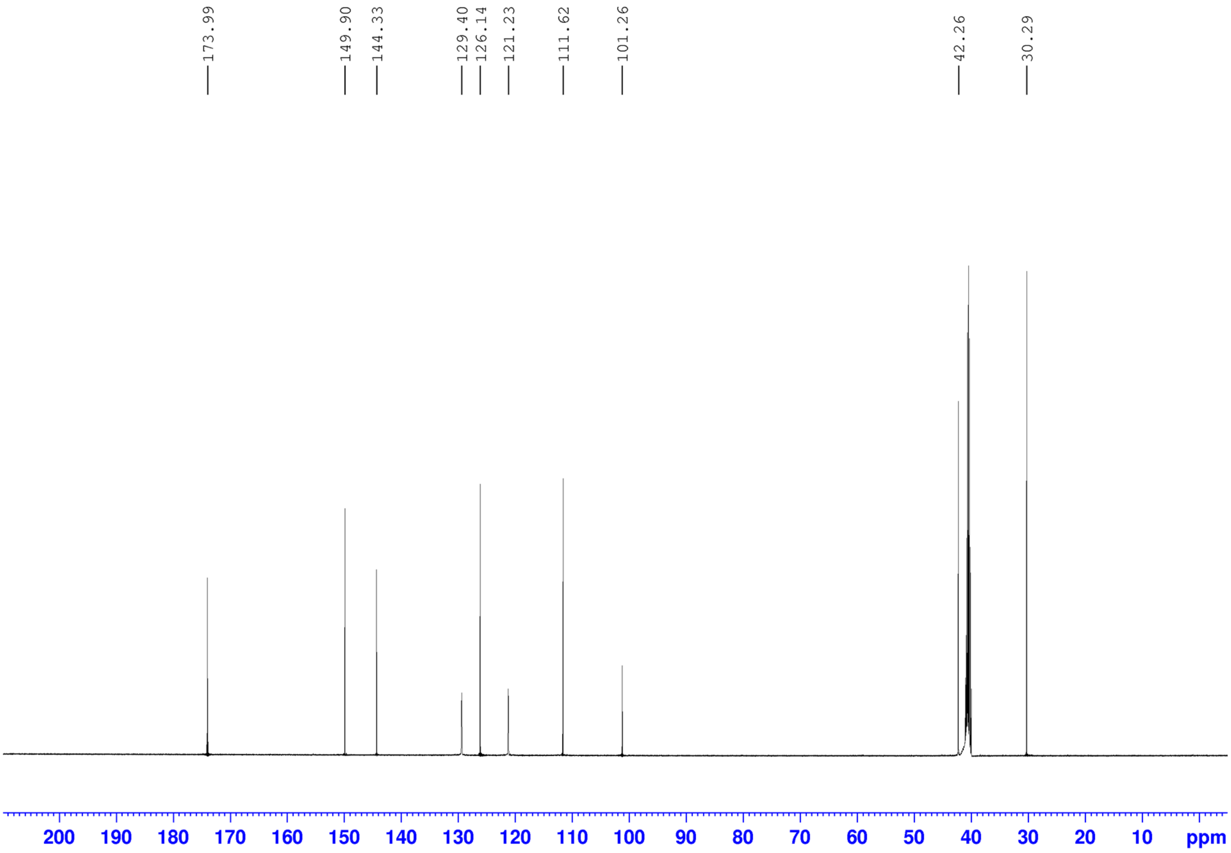

COSY NMR (300 MHz)

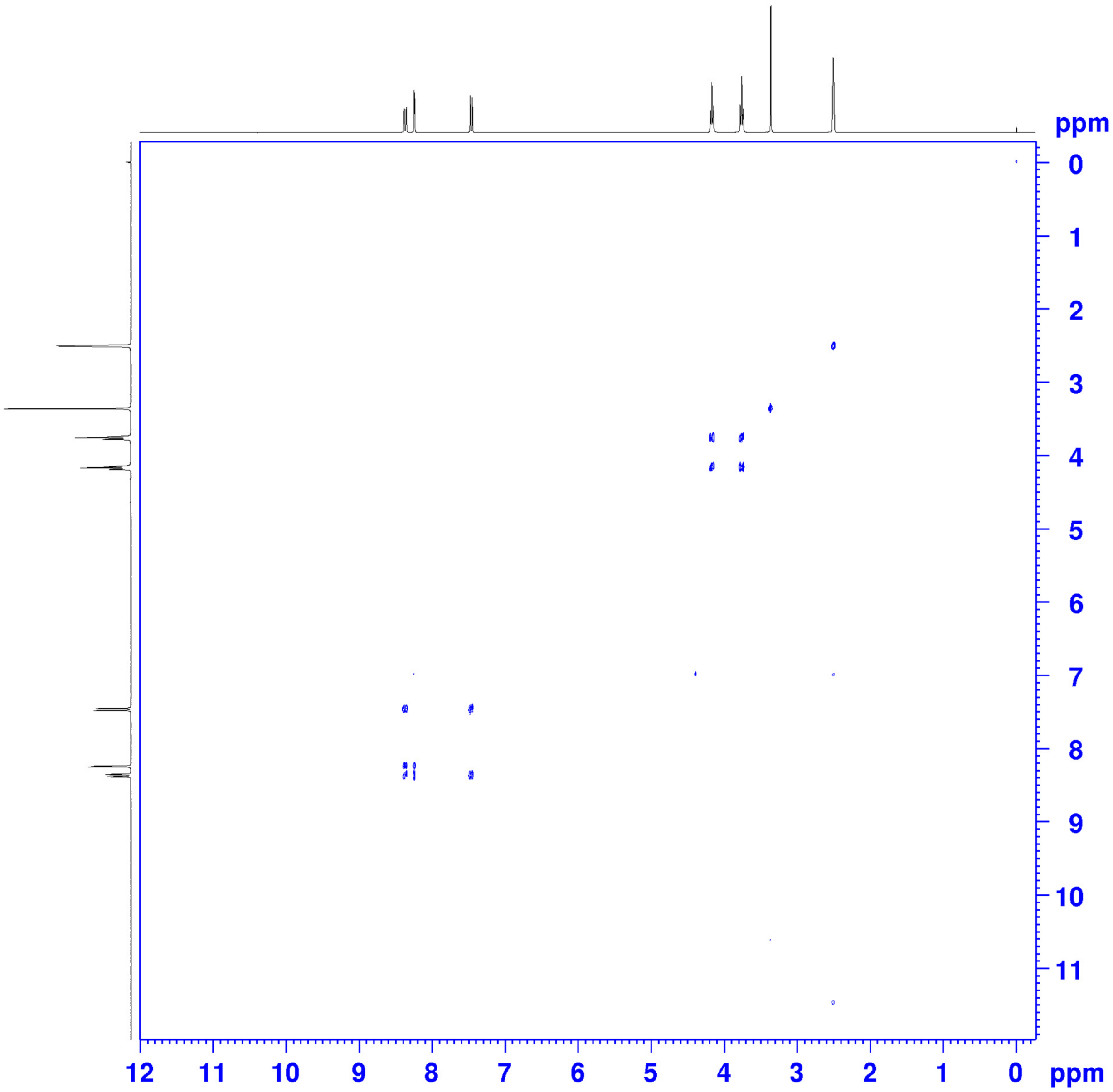

HRMS

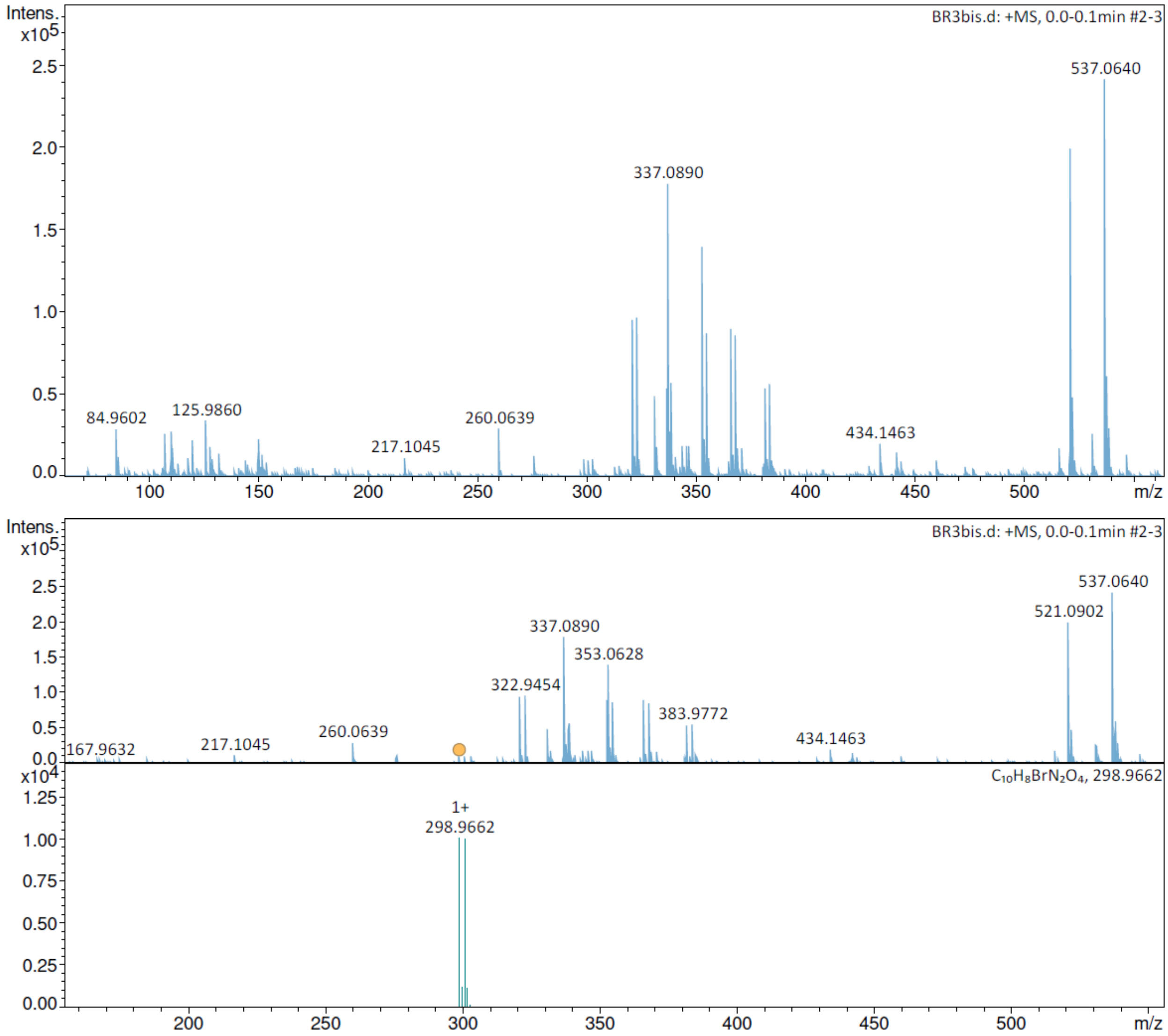

Intens.
x10^5
BR3bis.d: +MS, 0.0-0.1min #2-3
537.0640
337.0890
434.1463
260.0639
217.1045
125.9860
84.9602
m/z
521.0902
353.0628
322.9454
383.9772
167.9632
x10^4
C10H8BrN2O4, 298.9662
1+
298.9662

1-(2-Bromoethyl)-5-chloroisatin (9)

^{1}H NMR (300 MHz)

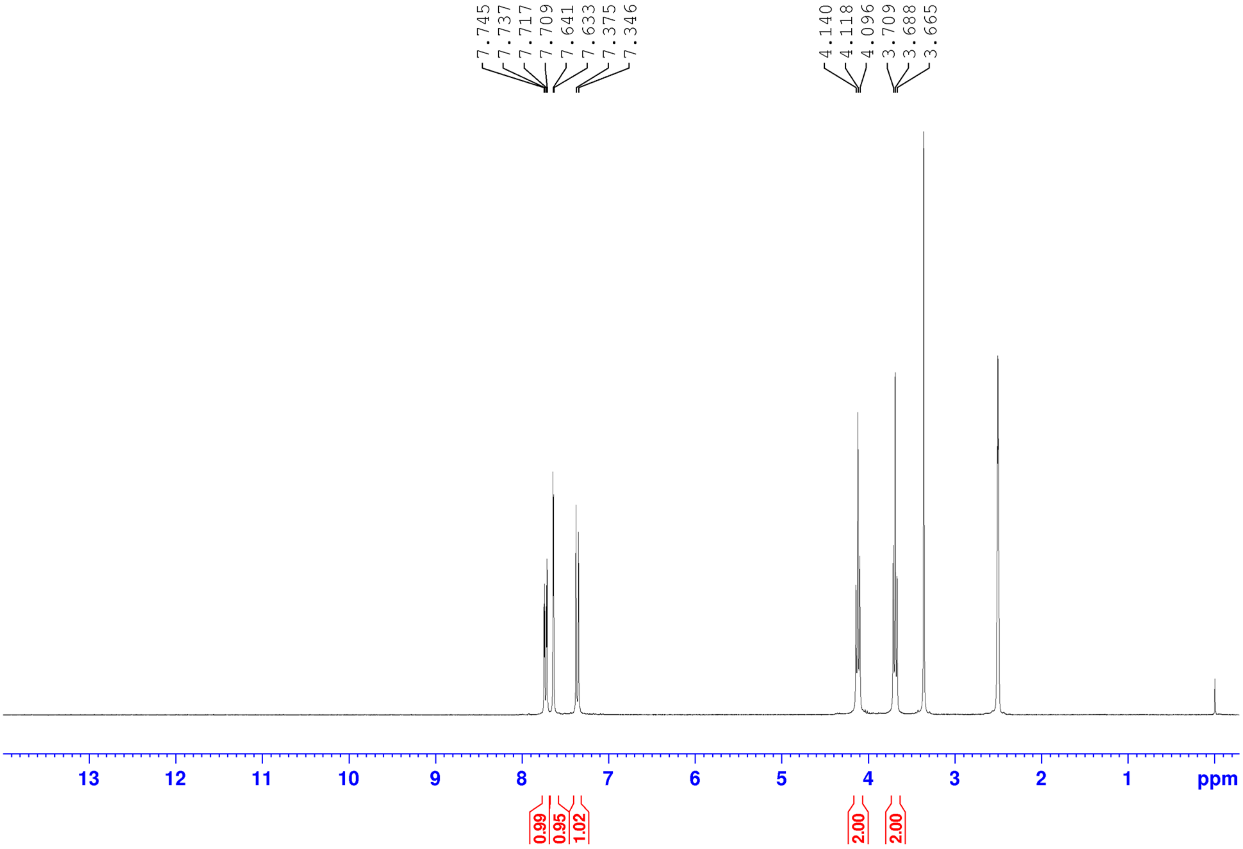

^{13}C NMR (500 MHz)

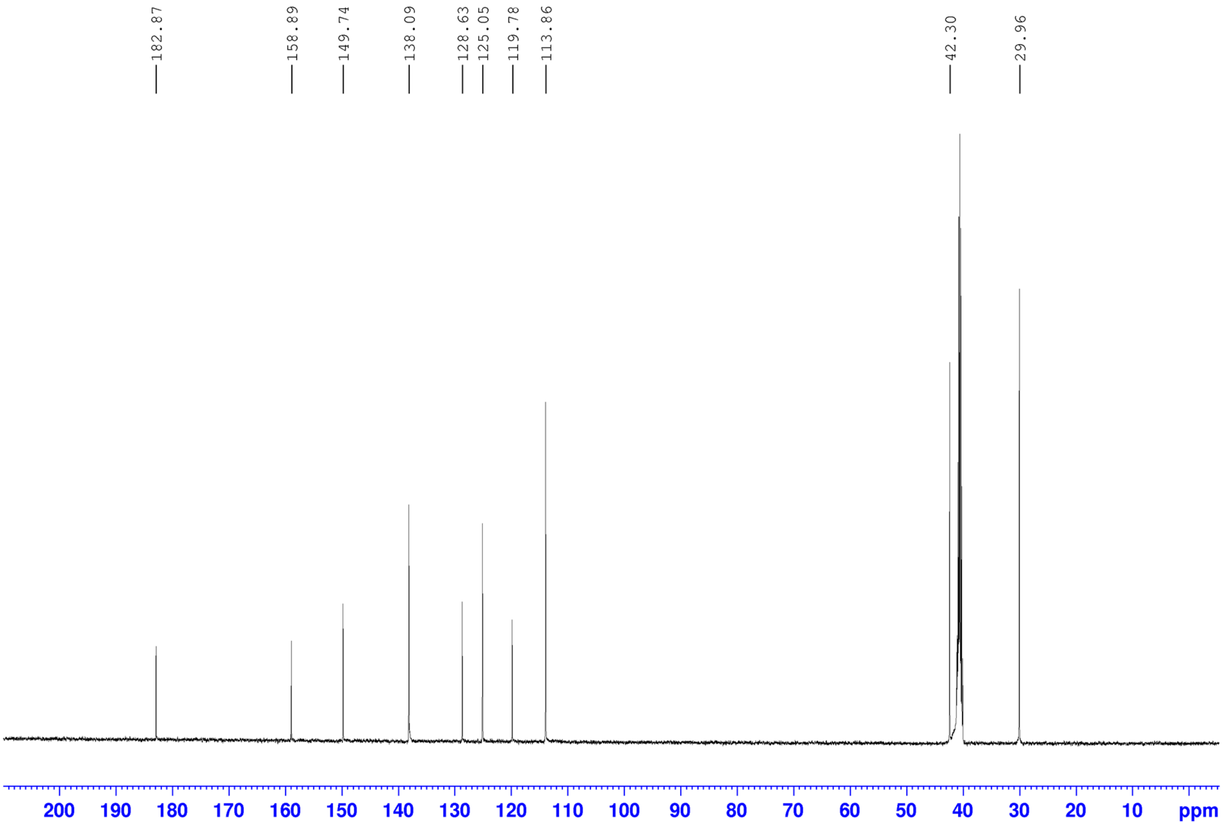

COSY NMR (300 MHz)

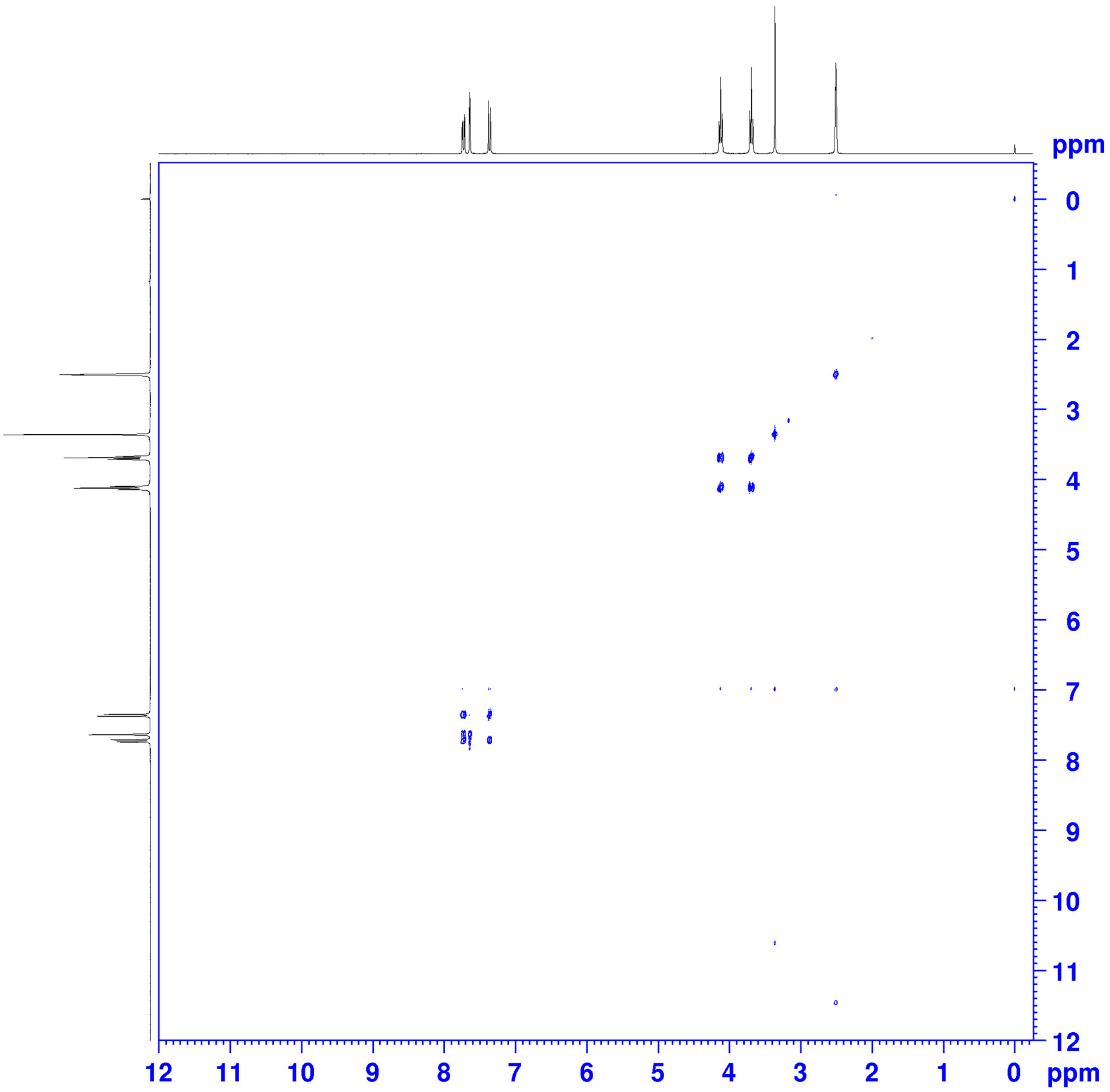

Characterization spectra of 1-(2-azidoethyl) isatins 10-13.

1-(2-azidoethyl)isatin (10)

^{1}H NMR (300 MHz)

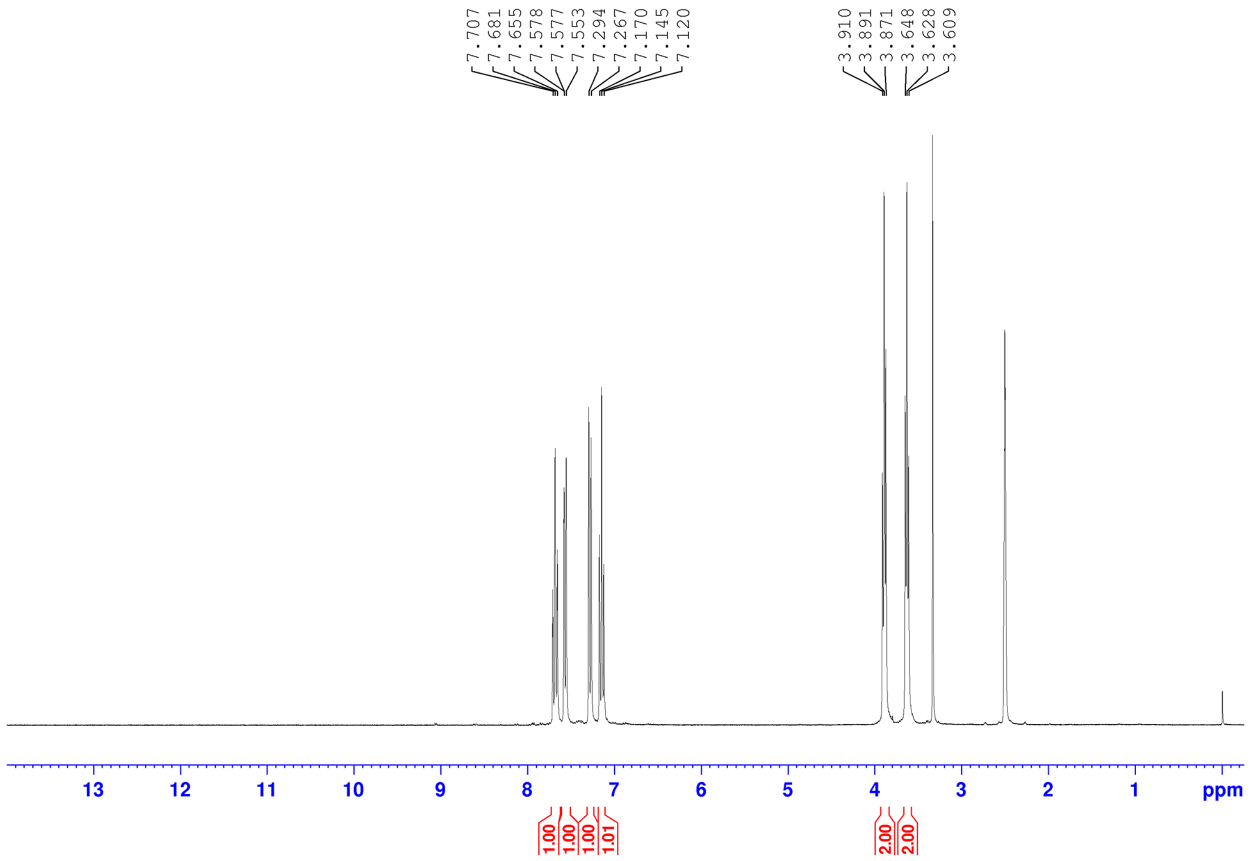

^{13}C NMR (125 MHz)

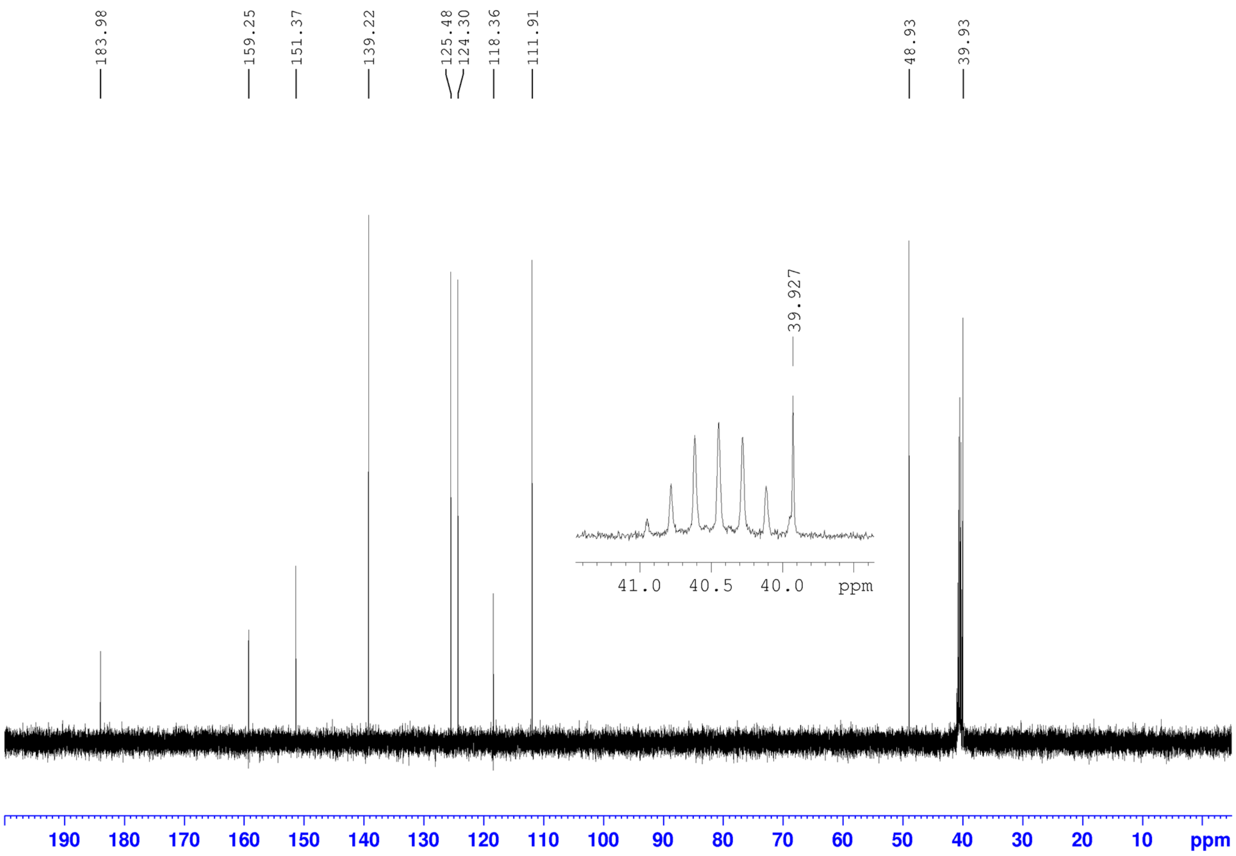

COSY NMR (300 MHz)

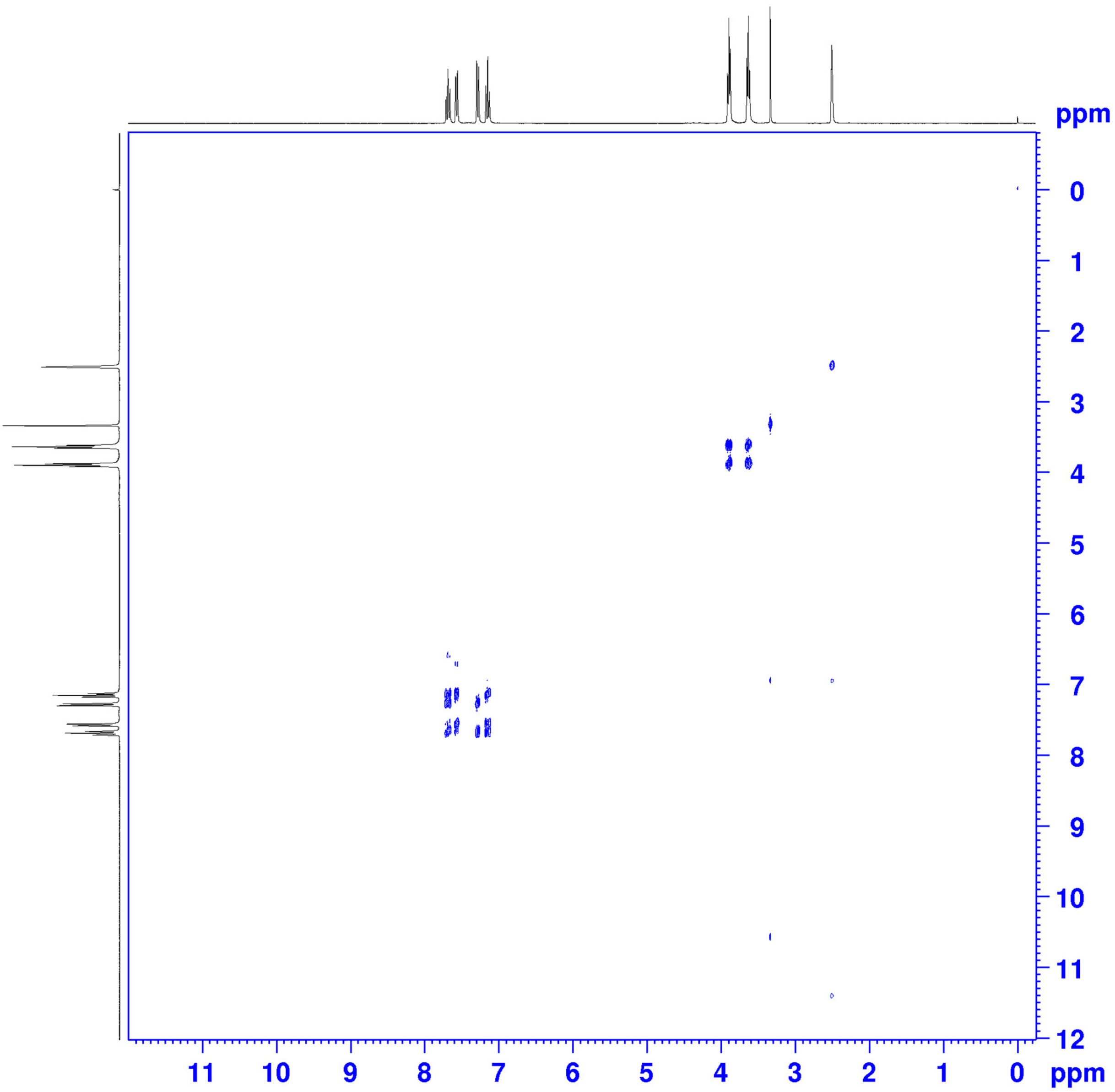

HRMS

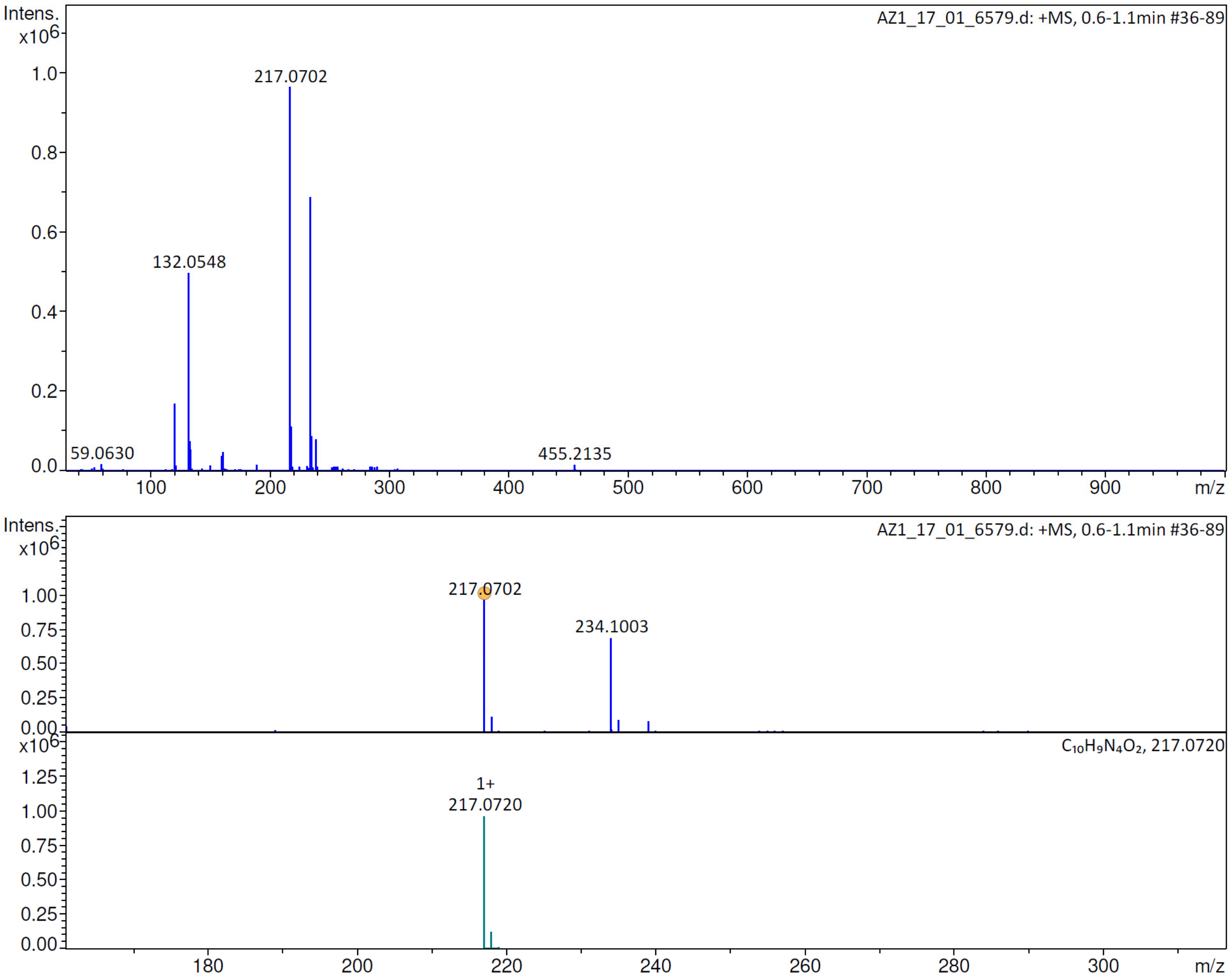

Intens.
x10^6
AZ1_17_01_6579.d: +MS, 0.6-1.1min #36-89
217.0702
132.0548
59.0630
455.2135
m/z
AZ1_17_01_6579.d: +MS, 0.6-1.1min #36-89
217.0702
234.1003
C10H9N4O2, 217.0720
1+
217.0720
m/z

1-(2-azidoethyl)-5-methylisatin (11)

^{1}H NMR (300 MHz)

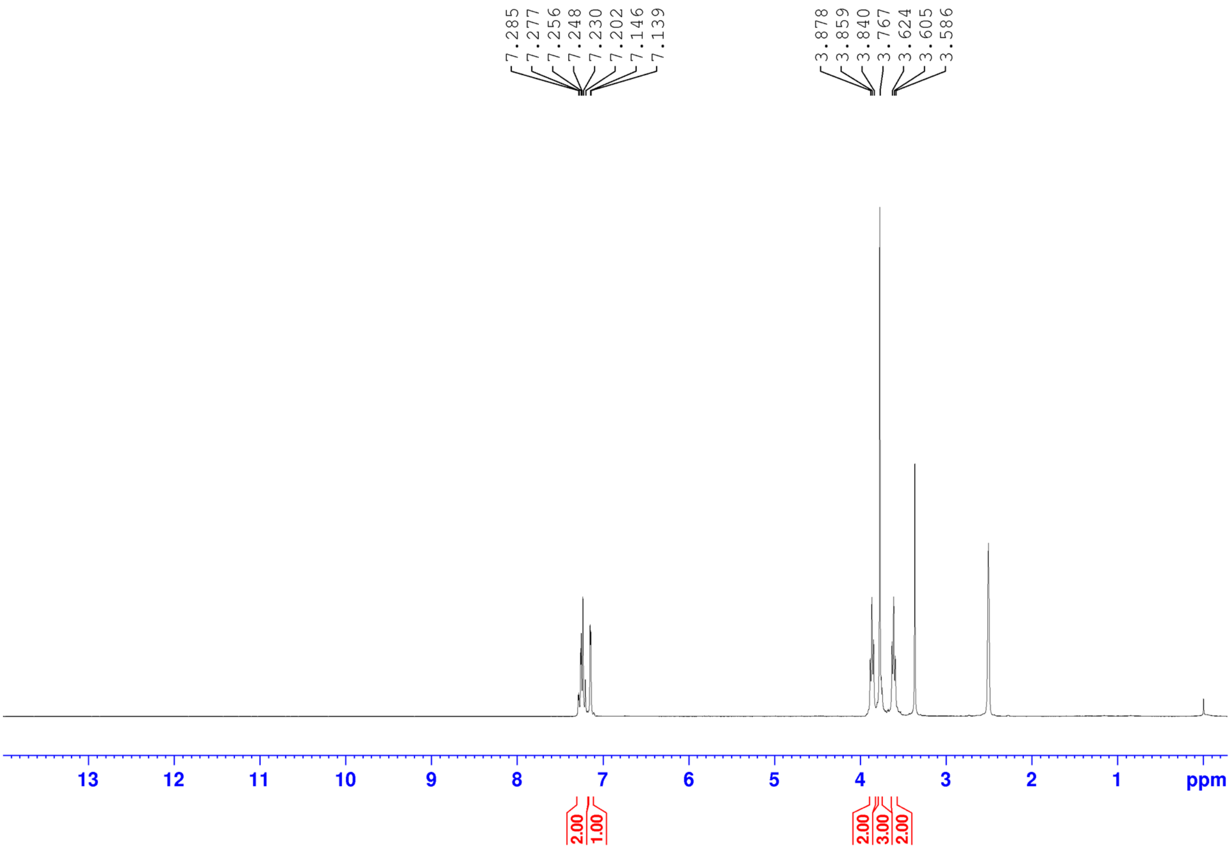

^{13}C NMR (125 MHz)

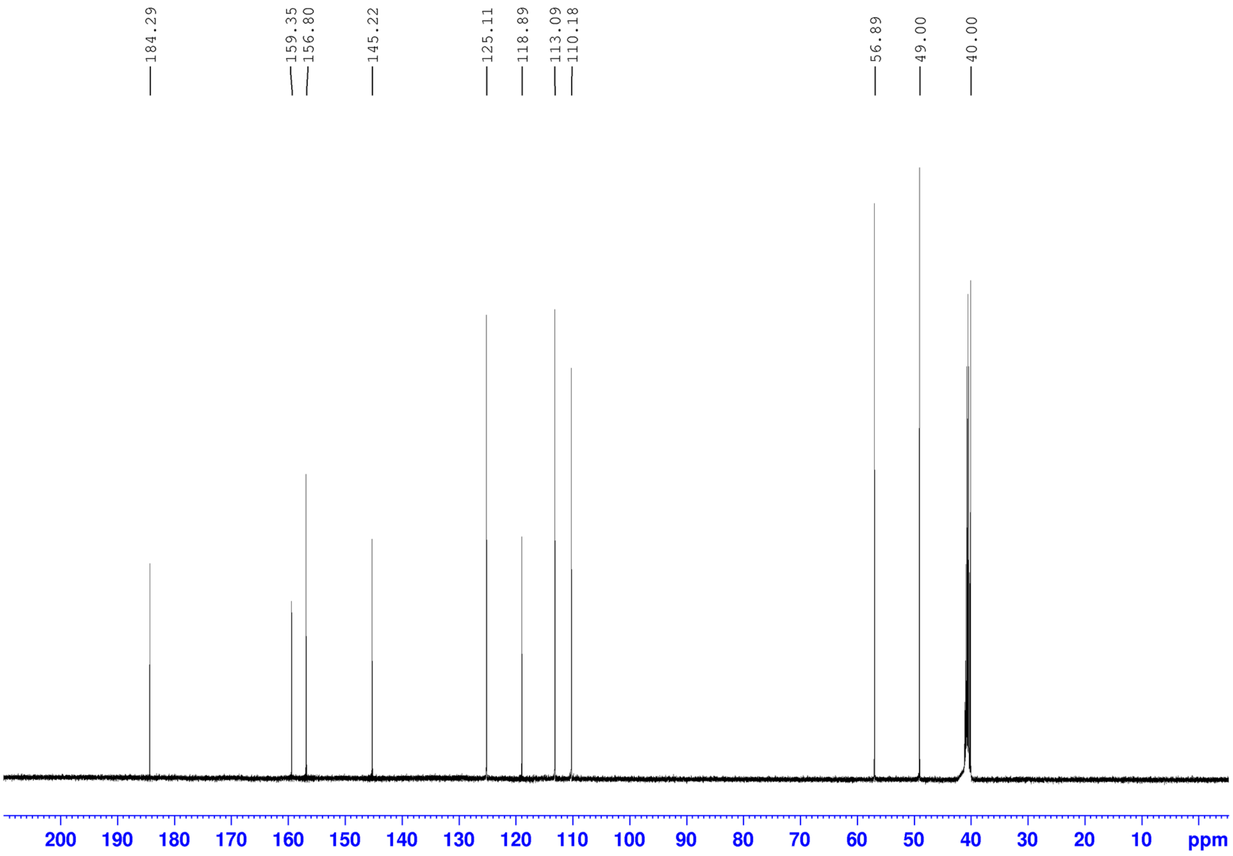

COSY NMR (300 MHz)

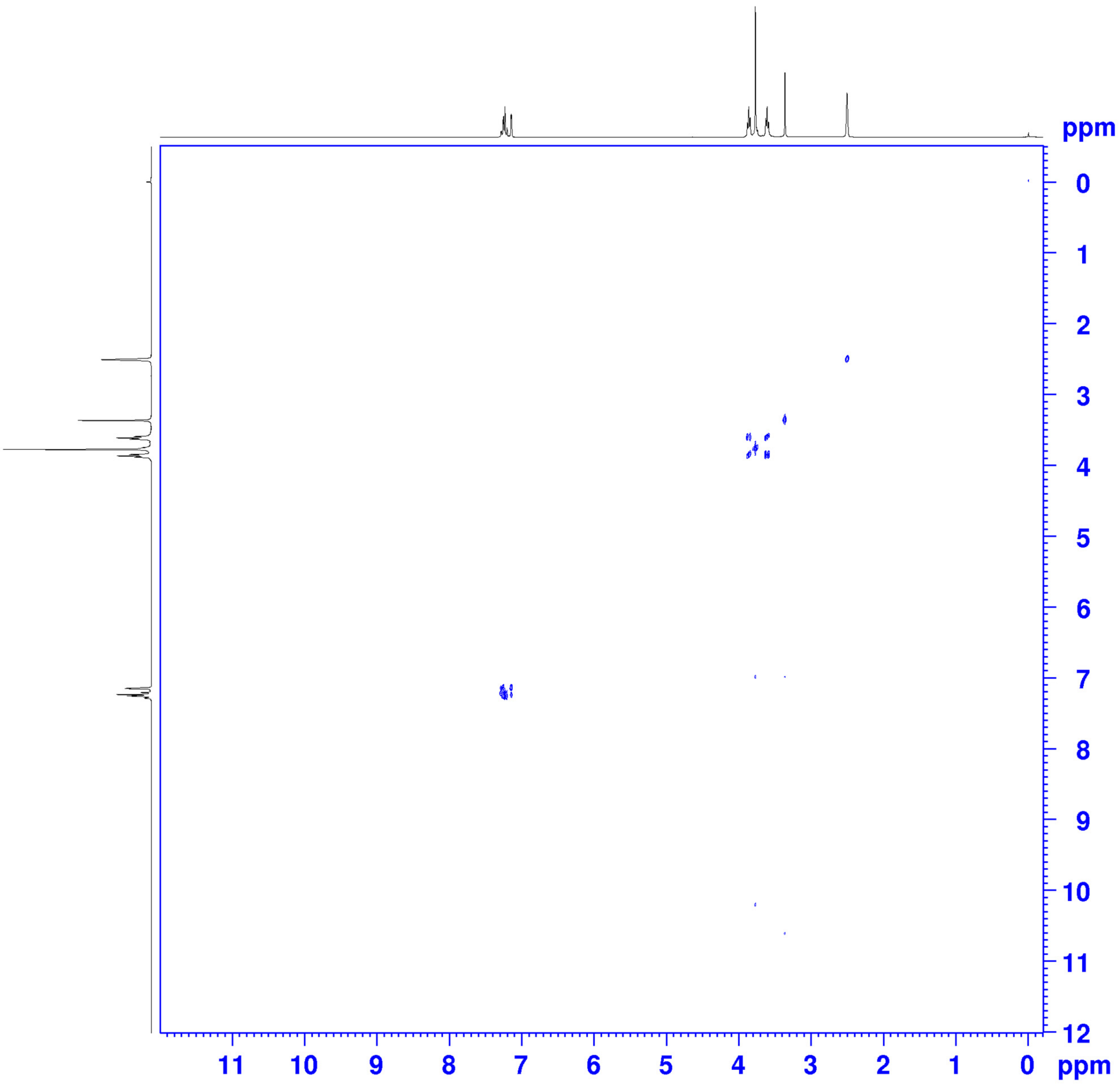

HRMS

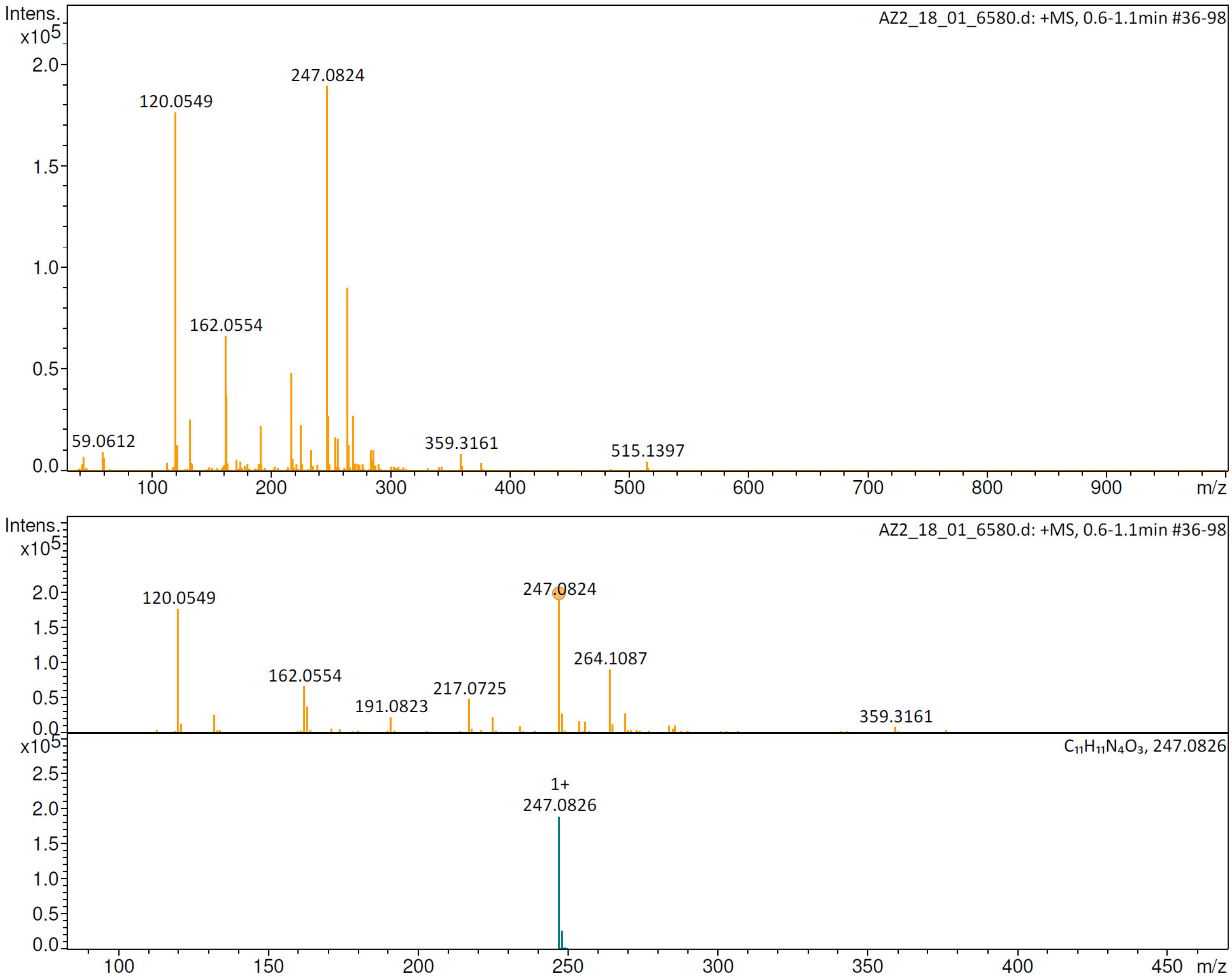
Intens.
x10^5
2.0
1.5
1.0
0.5
0.0
AZ2_18_01_6580.d: +MS, 0.6-1.1min #36-98
247.0824
120.0549
162.0554
59.0612
359.3161
515.1397
100
200
300
400
500
600
700
800
900
m/z
Intens.
x10^5
AZ2_18_01_6580.d: +MS, 0.6-1.1min #36-98
247.0824
120.0549
264.1087
162.0554
217.0725
191.0823
359.3161
C11H11N4O3, 247.0826
1+
247.0826
2.5
100
150
200
250
300
350
400
450
m/z

1-(2-azidoethyl)-5-nitroisatin (12)

^{1}H NMR (300 MHz)

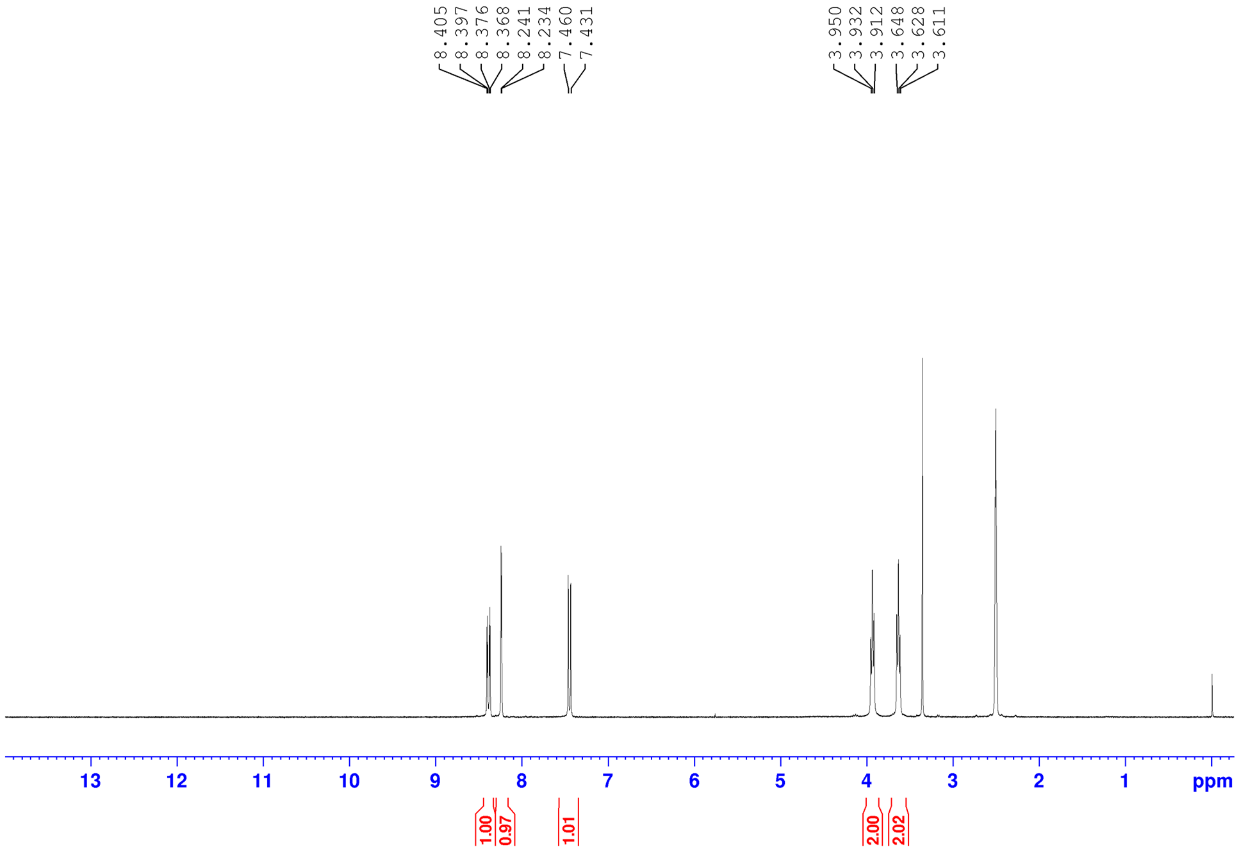

^{13}C NMR (125 MHz)

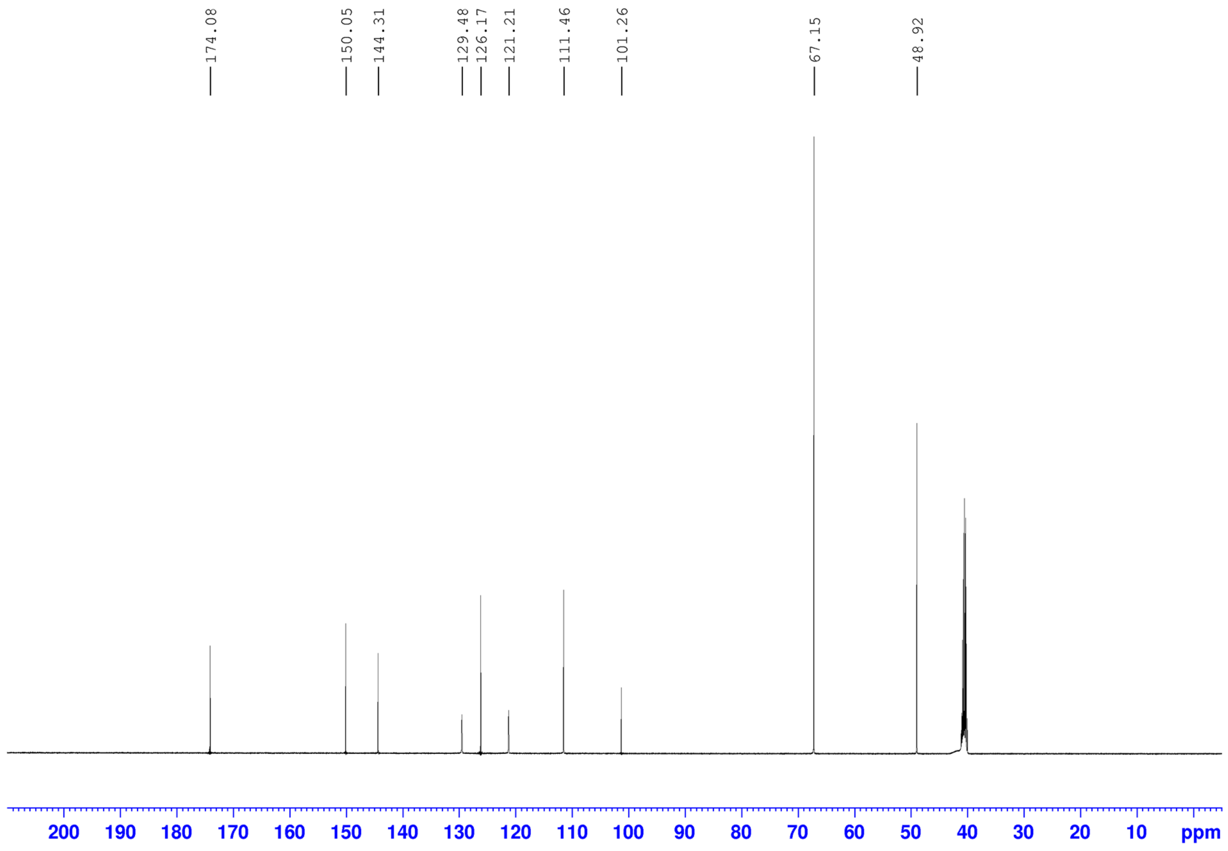

COSY NMR (300 MHz)

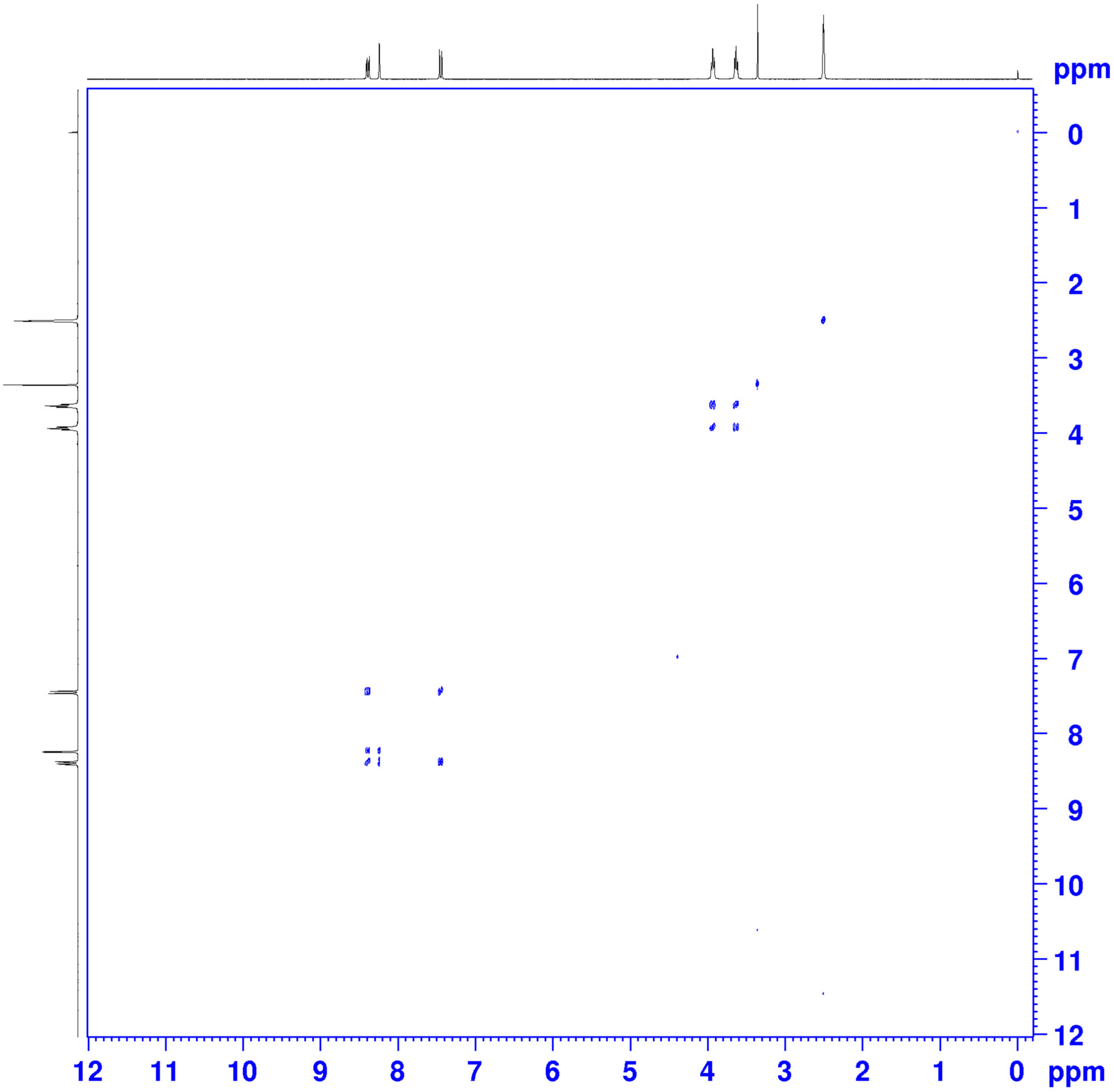

HRMS

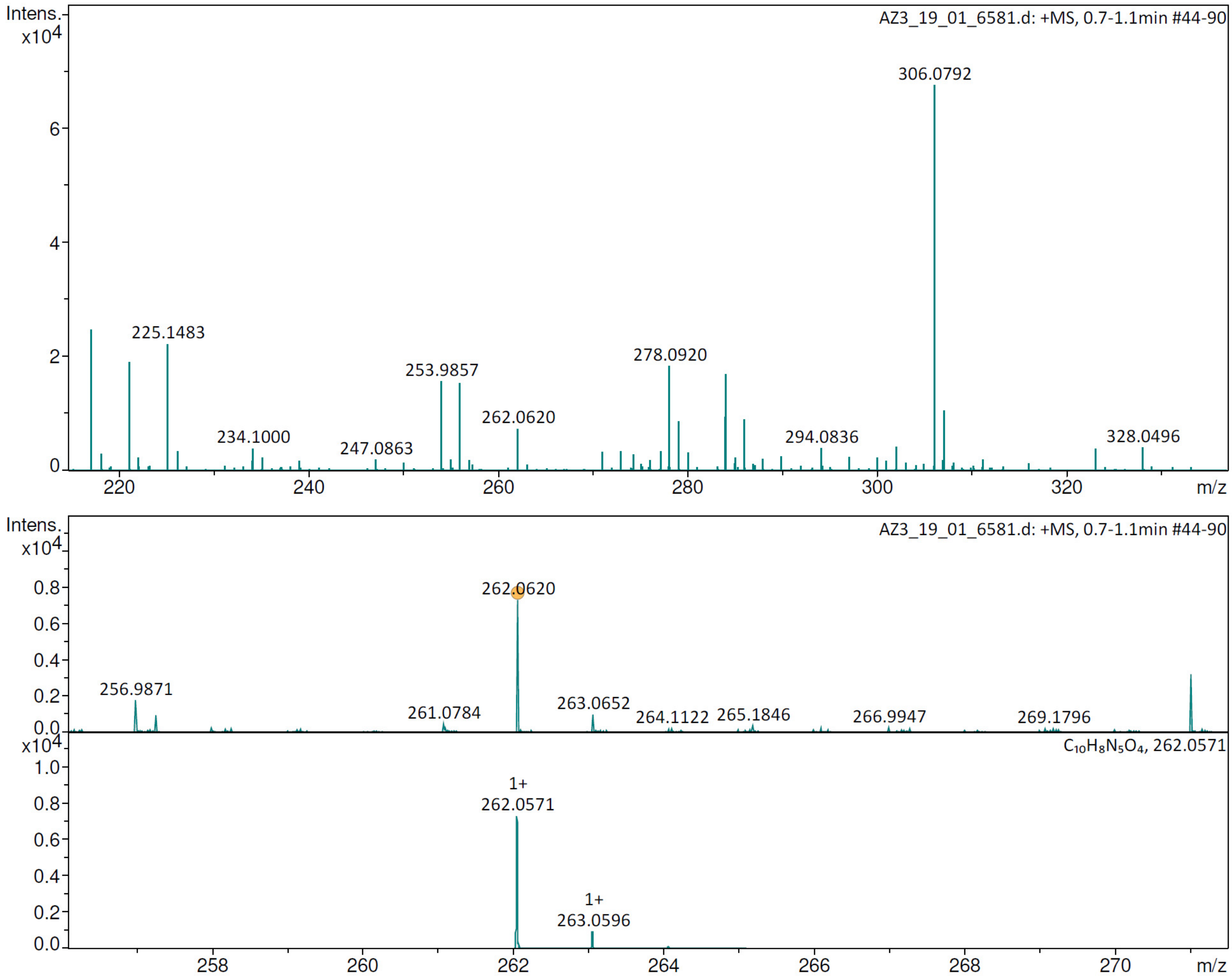

Intens.
x10^4
AZ3_19_01_6581.d: +MS, 0.7-1.1min #44-90
306.0792
225.1483
253.9857
278.0920
262.0620
234.1000
247.0863
294.0836
328.0496
m/z
256.9871
261.0784
263.0652
264.1122
265.1846
266.9947
269.1796
C10H8N5O4, 262.0571
1+
262.0571
263.0596

1-(2-Azidoethyl)-5-chloroisatin (13)

^{1}H NMR (300 MHz)

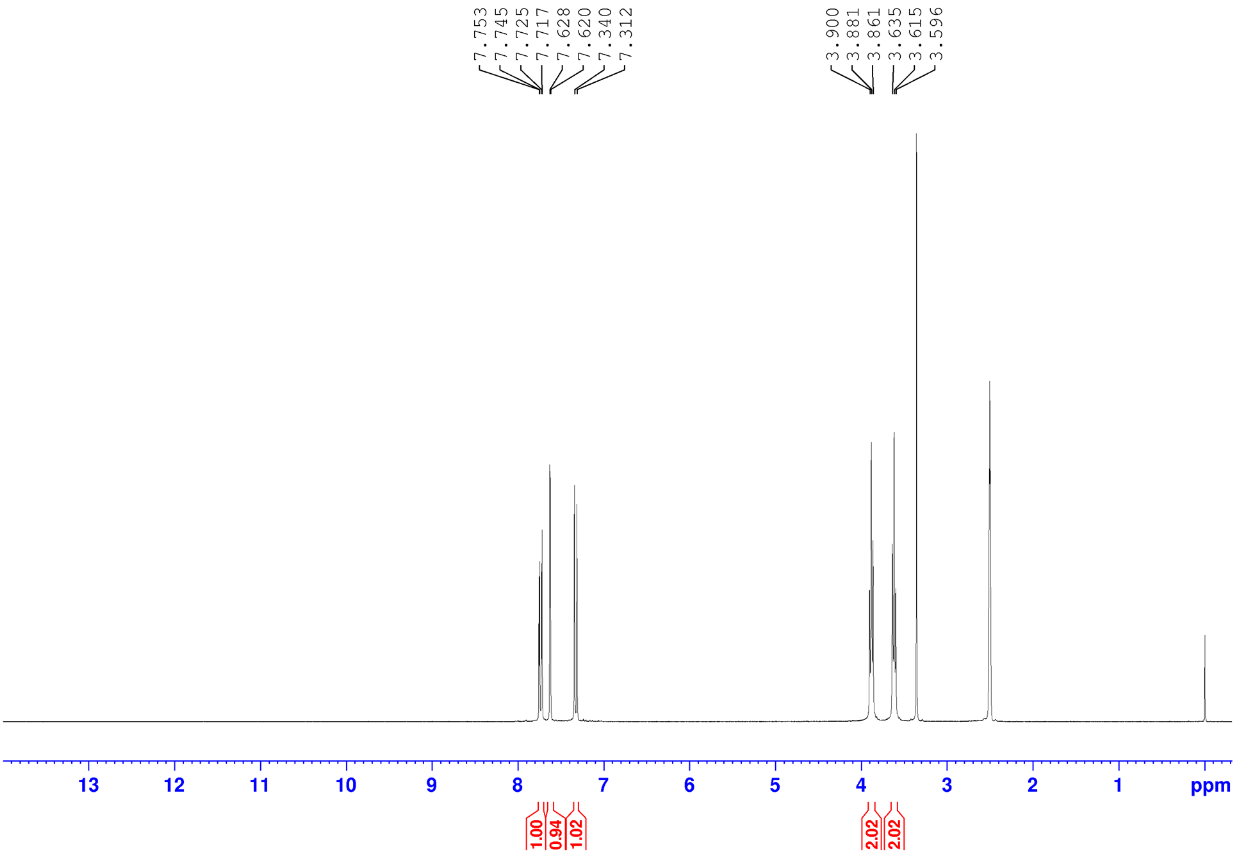

^{13}C NMR (125 MHz)

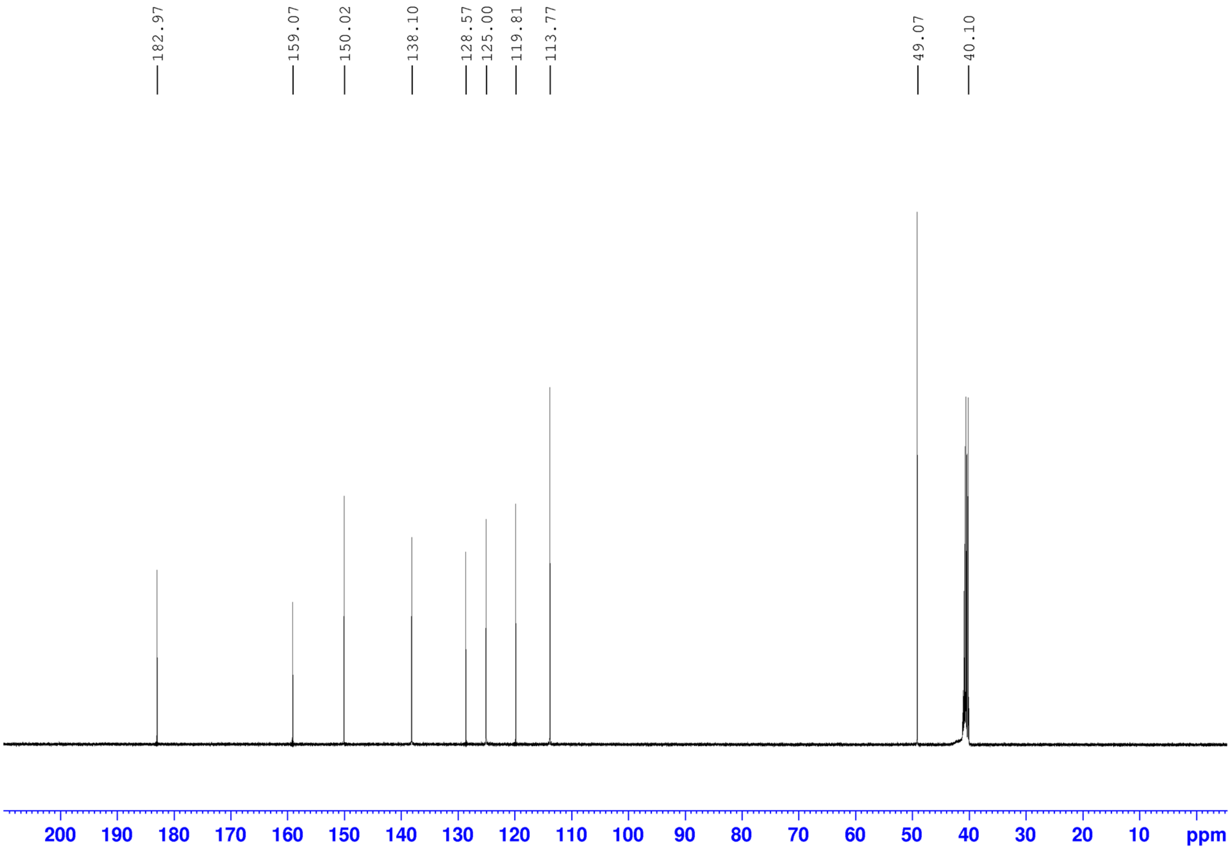

COSY NMR (300 MHz)

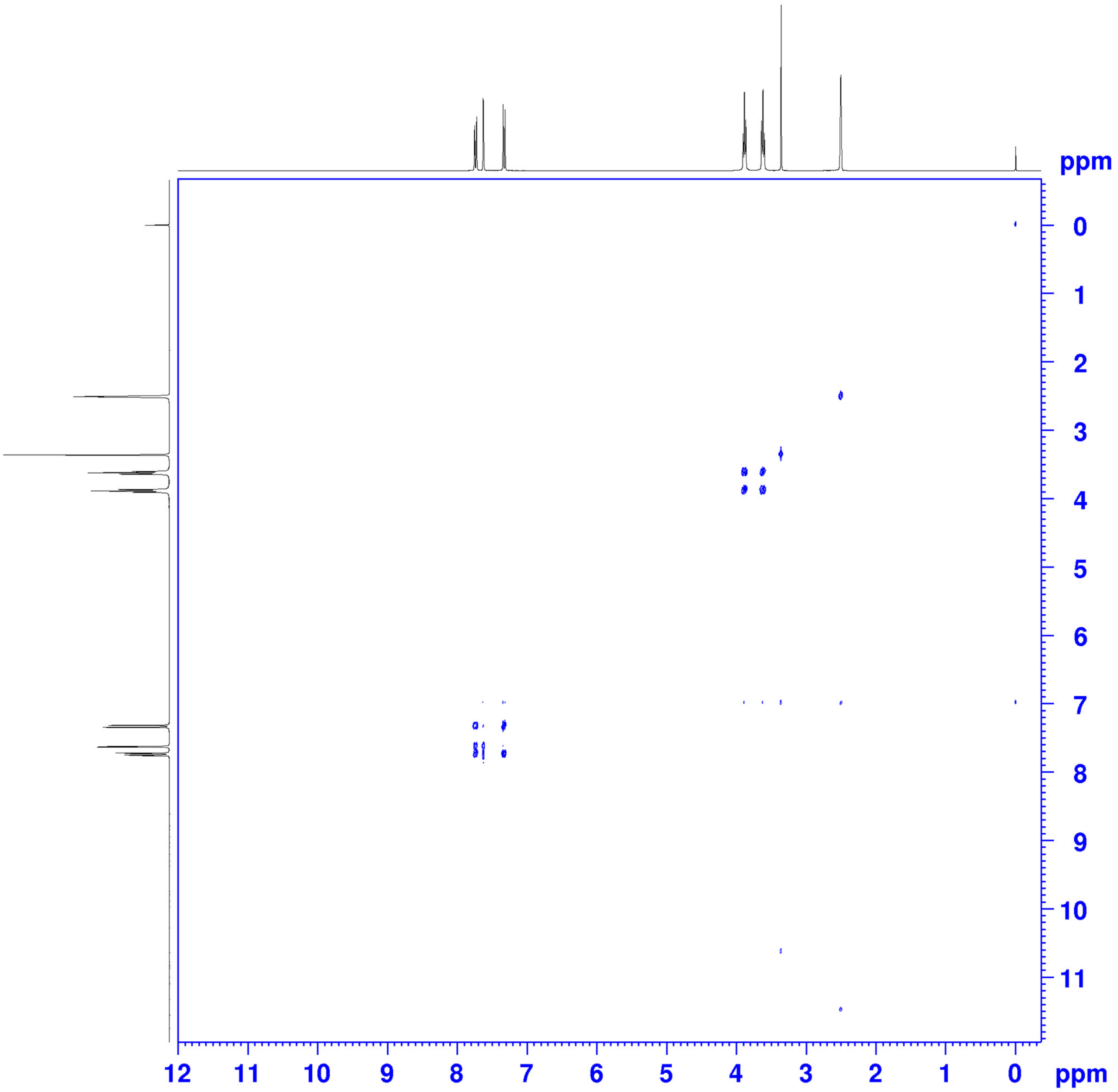

HRMS

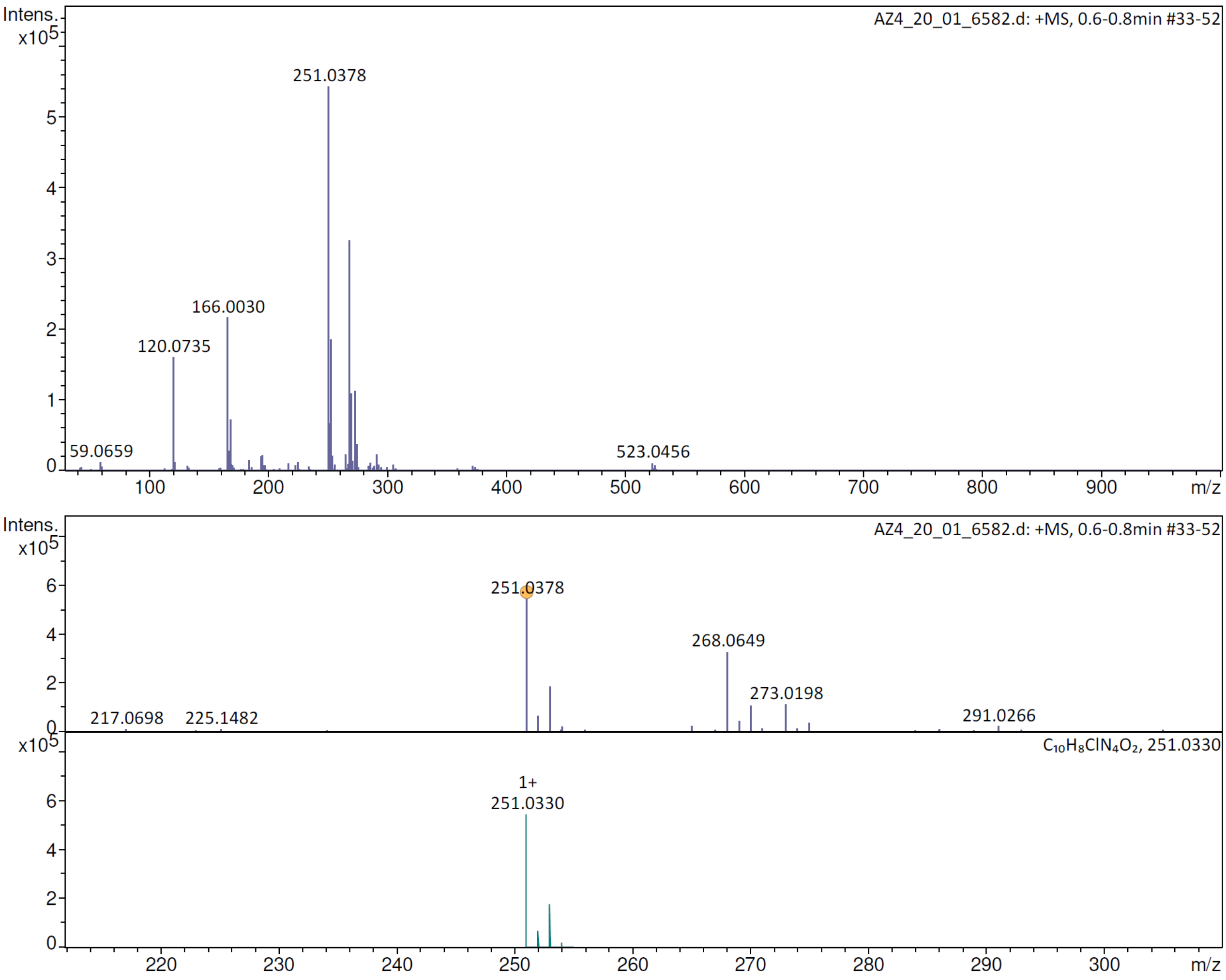

Intens.
x10^5
AZ4_20_01_6582.d: +MS, 0.6-0.8min #33-52
251.0378
166.0030
120.0735
59.0659
523.0456
m/z
268.0649
273.0198
217.0698
225.1482
291.0266
C₁₀H₈ClN₄O₂, 251.0330
1+
251.0330

Characterization spectra of propargyl esters 20-22.

Propargyl 3,4,5-trihydroxybenzoate (20)

^{1}H NMR (300 MHz)

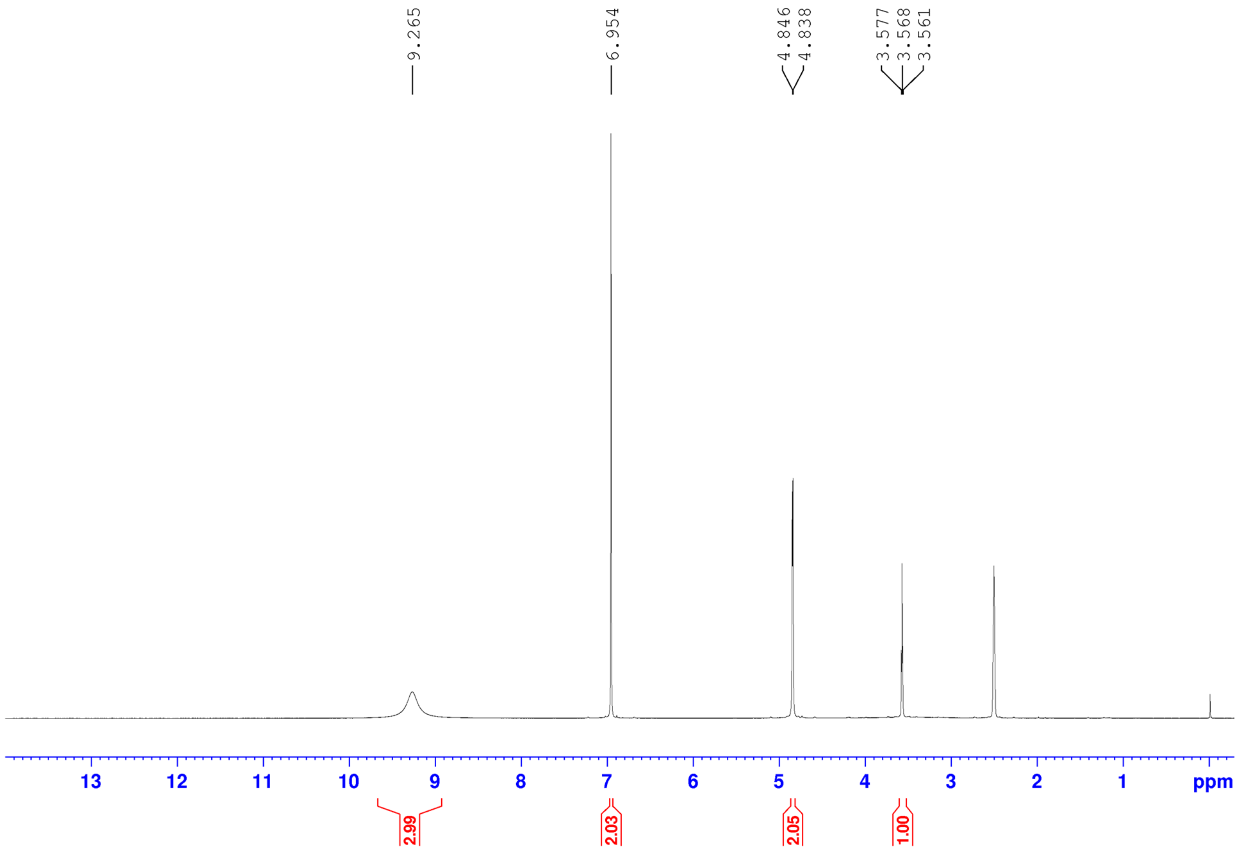

^{13}C NMR (125 MHz)

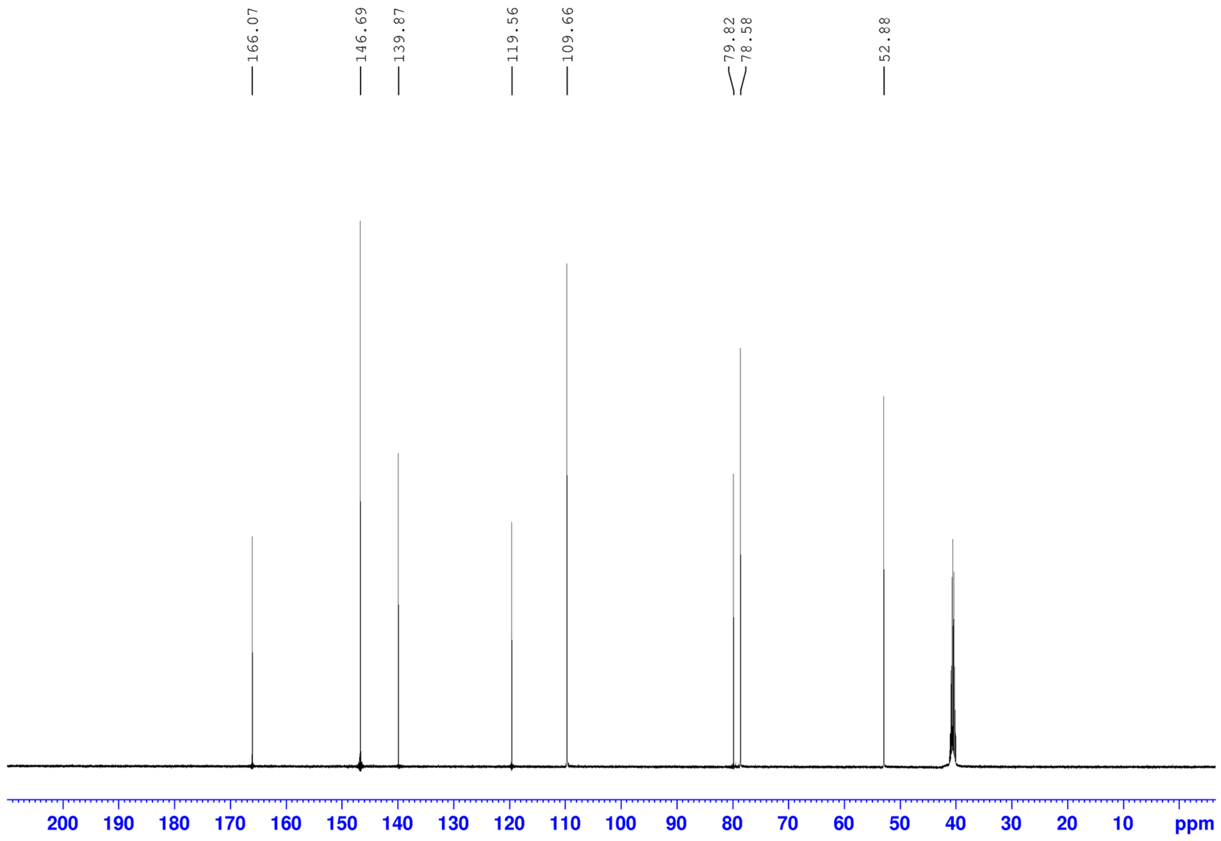

COSY NMR (300 MHz)

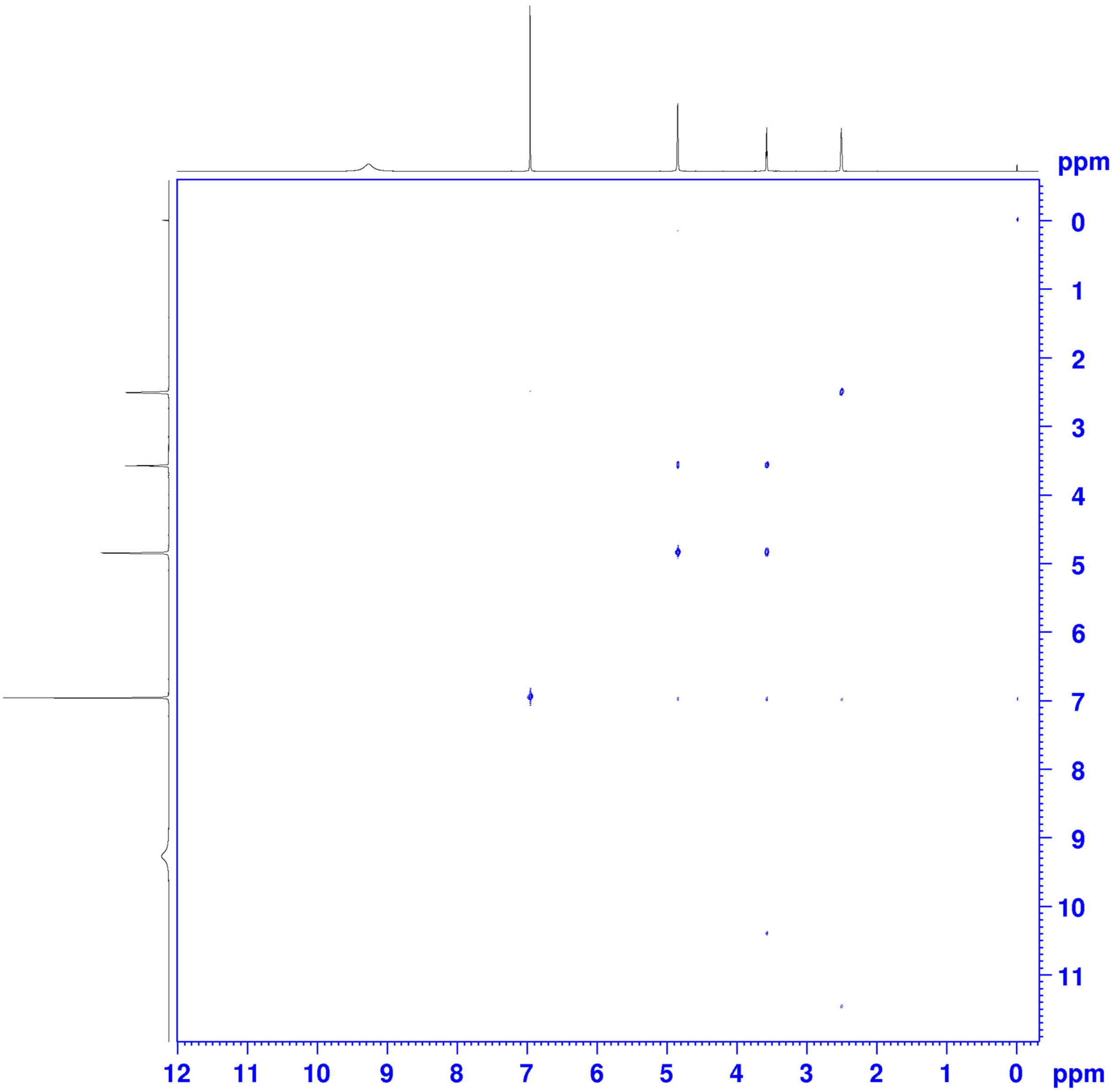

HRMS

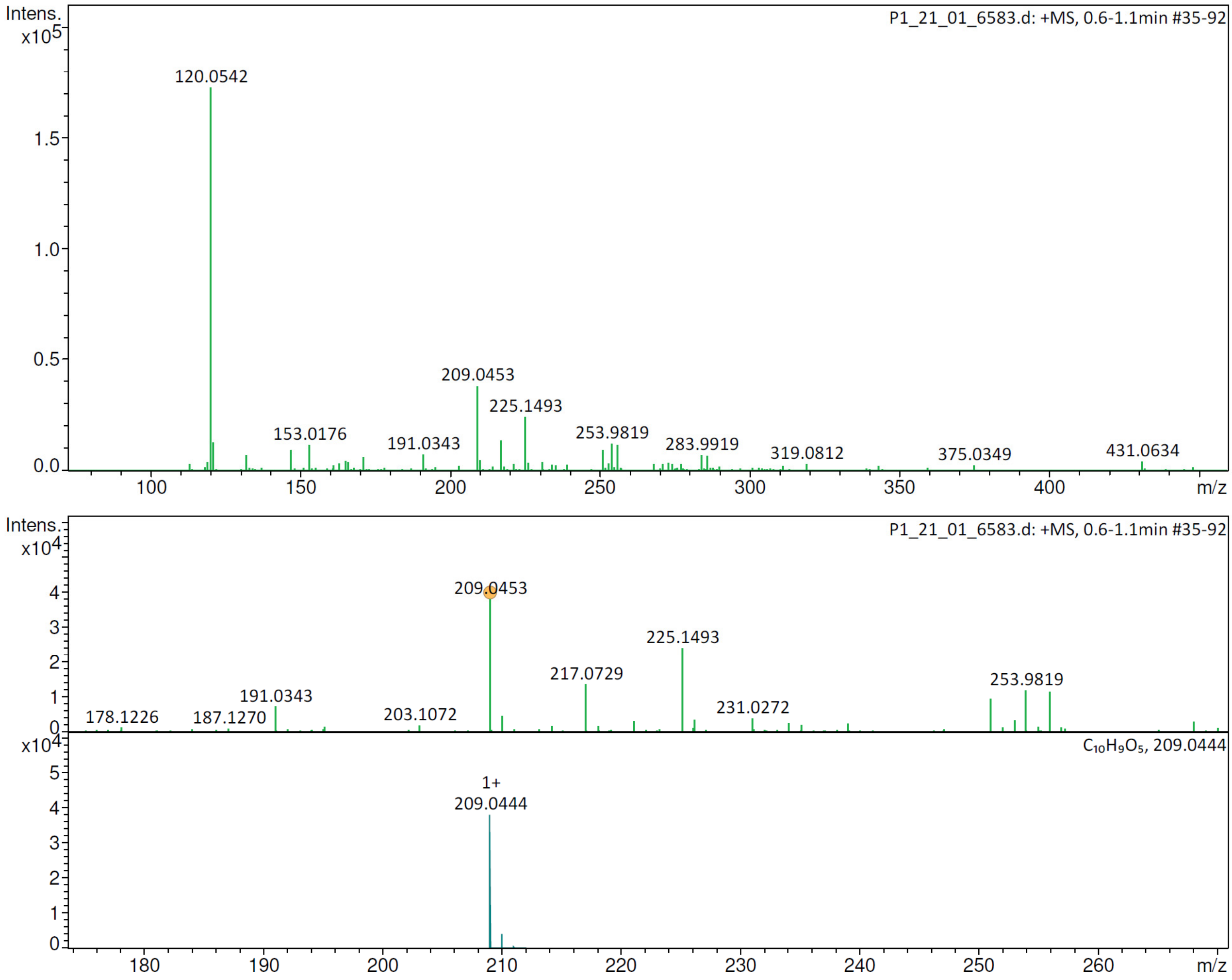
Intens.
x10^5
P1_21_01_6583.d: +MS, 0.6-1.1min #35-92
120.0542
209.0453
225.1493
153.0176
191.0343
253.9819
283.9919
319.0812
375.0349
431.0634
m/z
Intens.
x10^4
P1_21_01_6583.d: +MS, 0.6-1.1min #35-92
209.0453
225.1493
217.0729
253.9819
191.0343
231.0272
178.1226
187.1270
203.1072
C10H9O5, 209.0444
1+
209.0444
m/z

Propargyl 4-hydroxybenzoate (21)

^{1}H NMR (300 MHz)

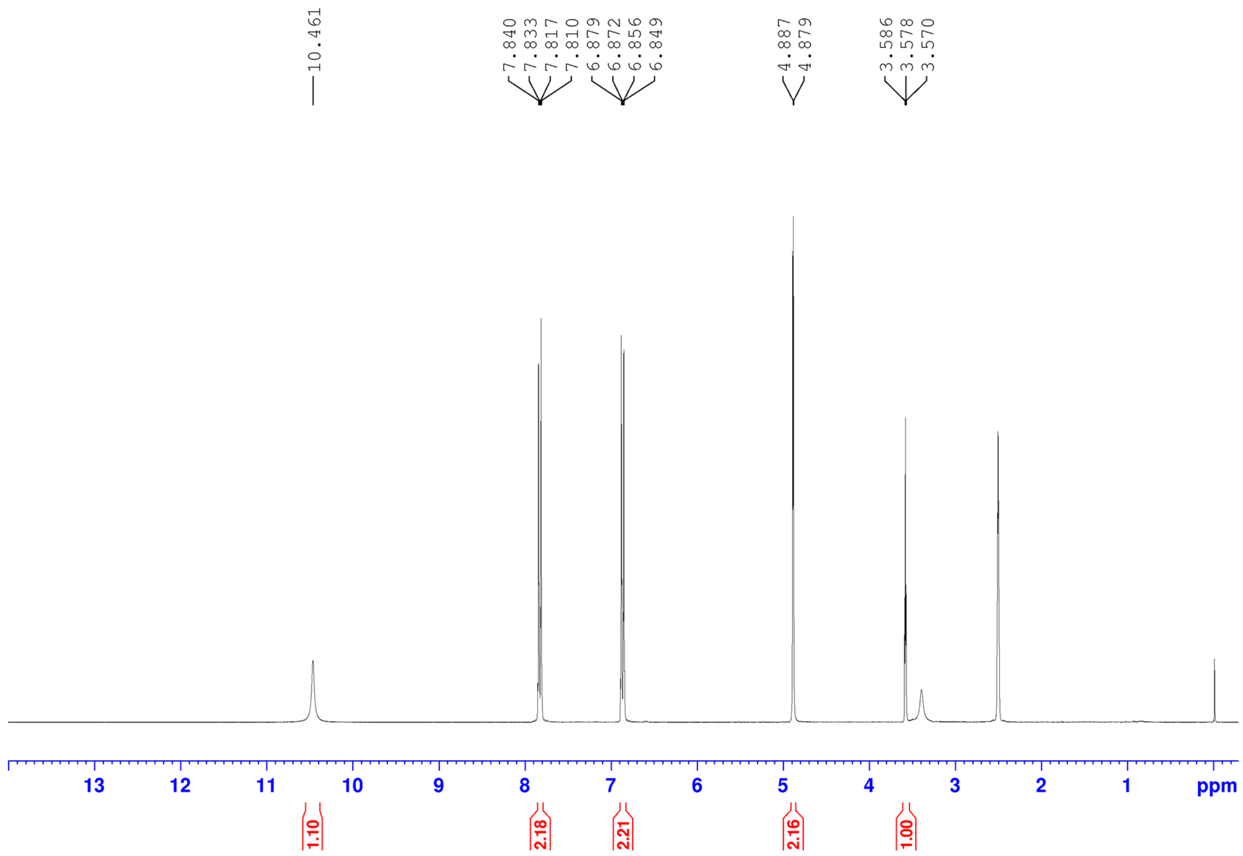

^{13}C NMR (125 MHz)

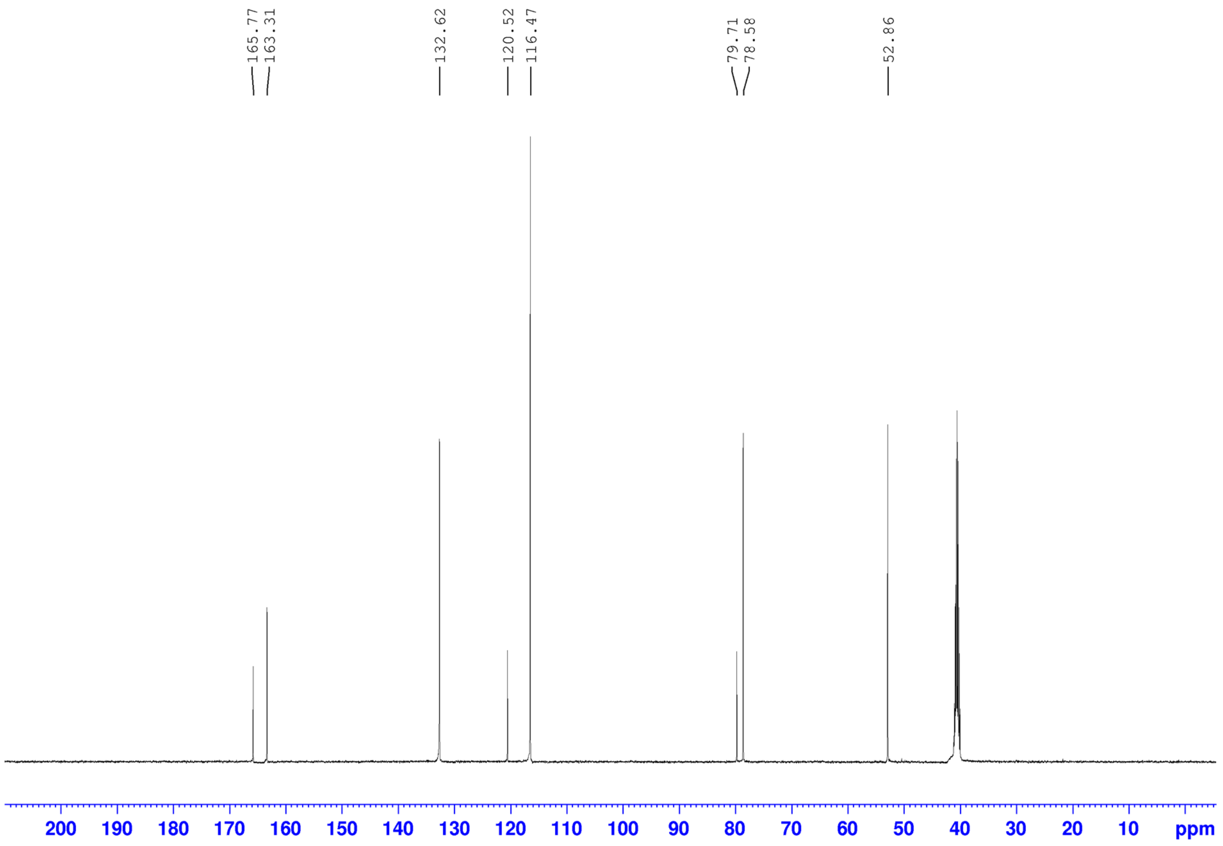

COSY NMR (300 MHz)

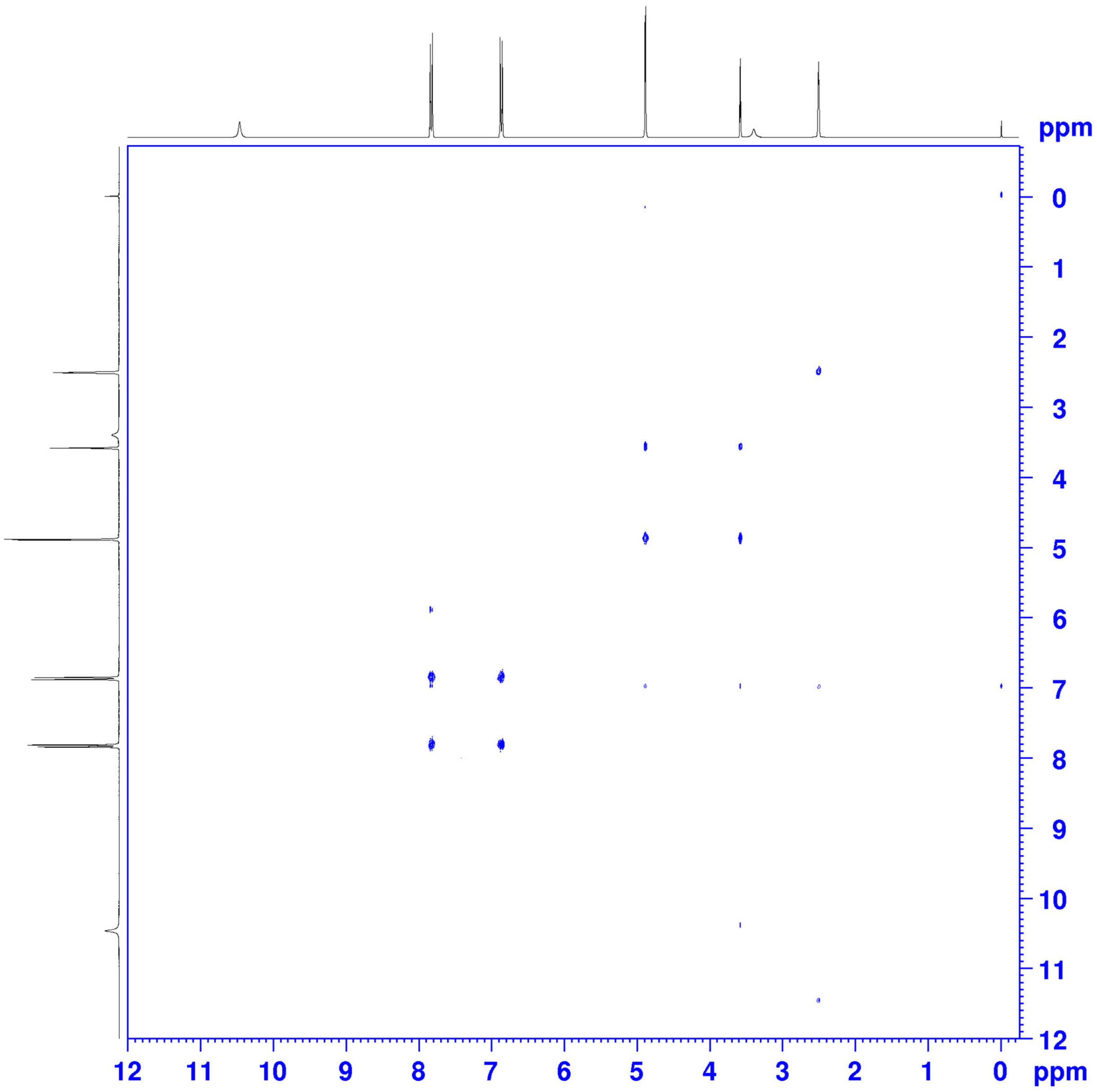

HRMS

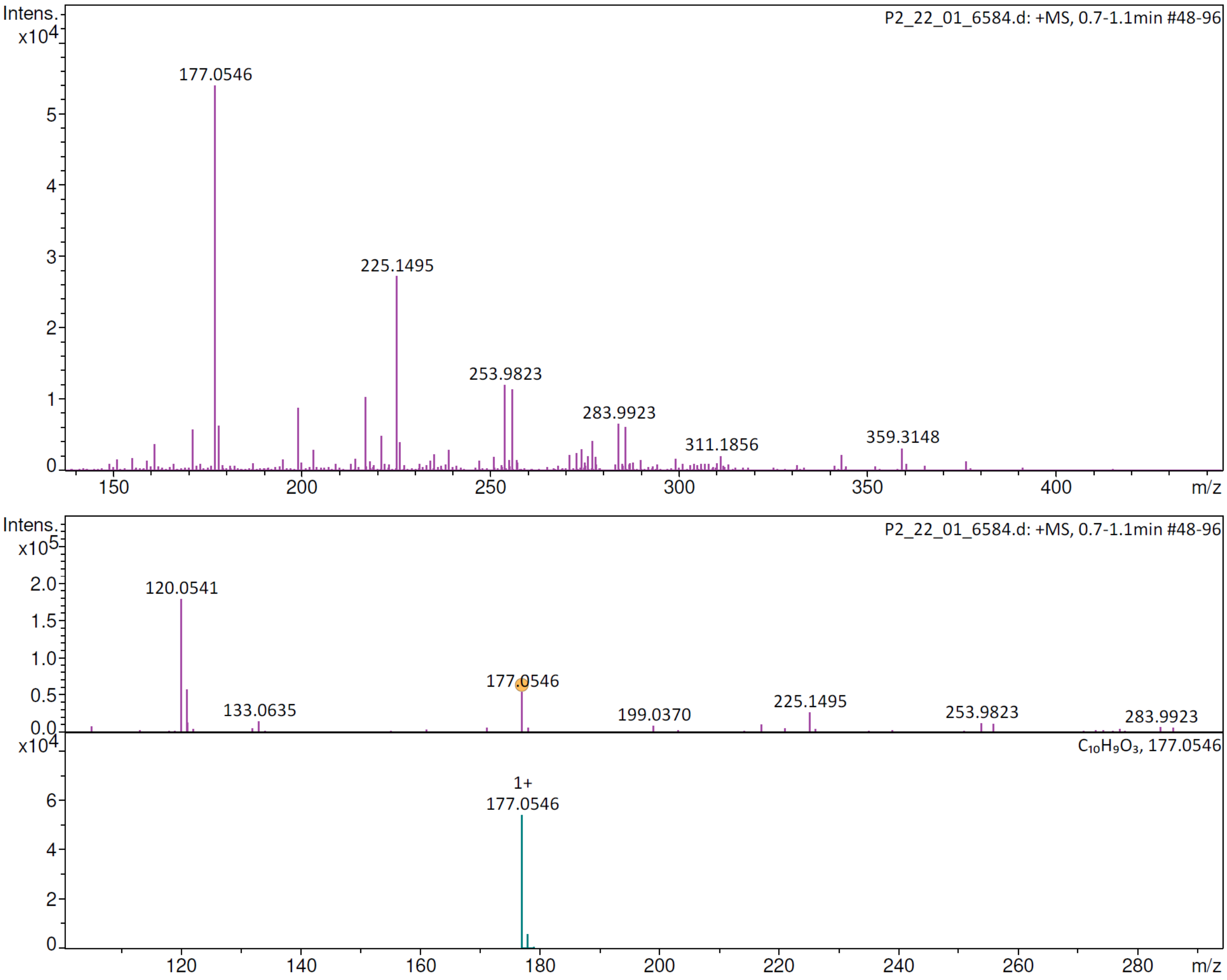

Intens.
x10^4
P2_22_01_6584.d: +MS, 0.7-1.1min #48-96
177.0546
225.1495
253.9823
283.9923
311.1856
359.3148
m/z
Intens.
x10^5
P2_22_01_6584.d: +MS, 0.7-1.1min #48-96
120.0541
133.0635
177.0546
199.0370
225.1495
253.9823
283.9923
x10^4
C10H9O3, 177.0546
1+
177.0546
m/z

Propargyl (*E*)-3-(3,4-dihydroxyphenyl)acrylate (22)

^{1}H NMR (300 MHz)

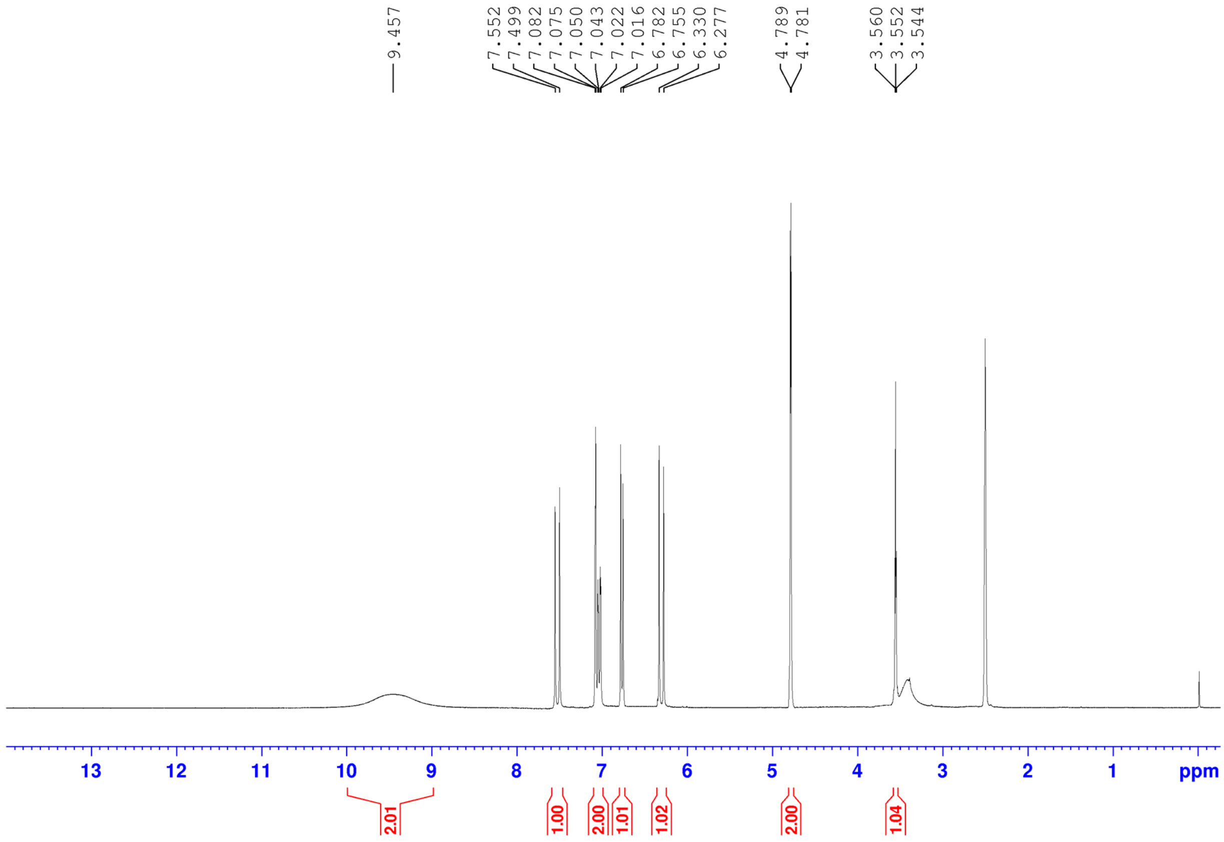

^{13}C NMR (125 MHz)

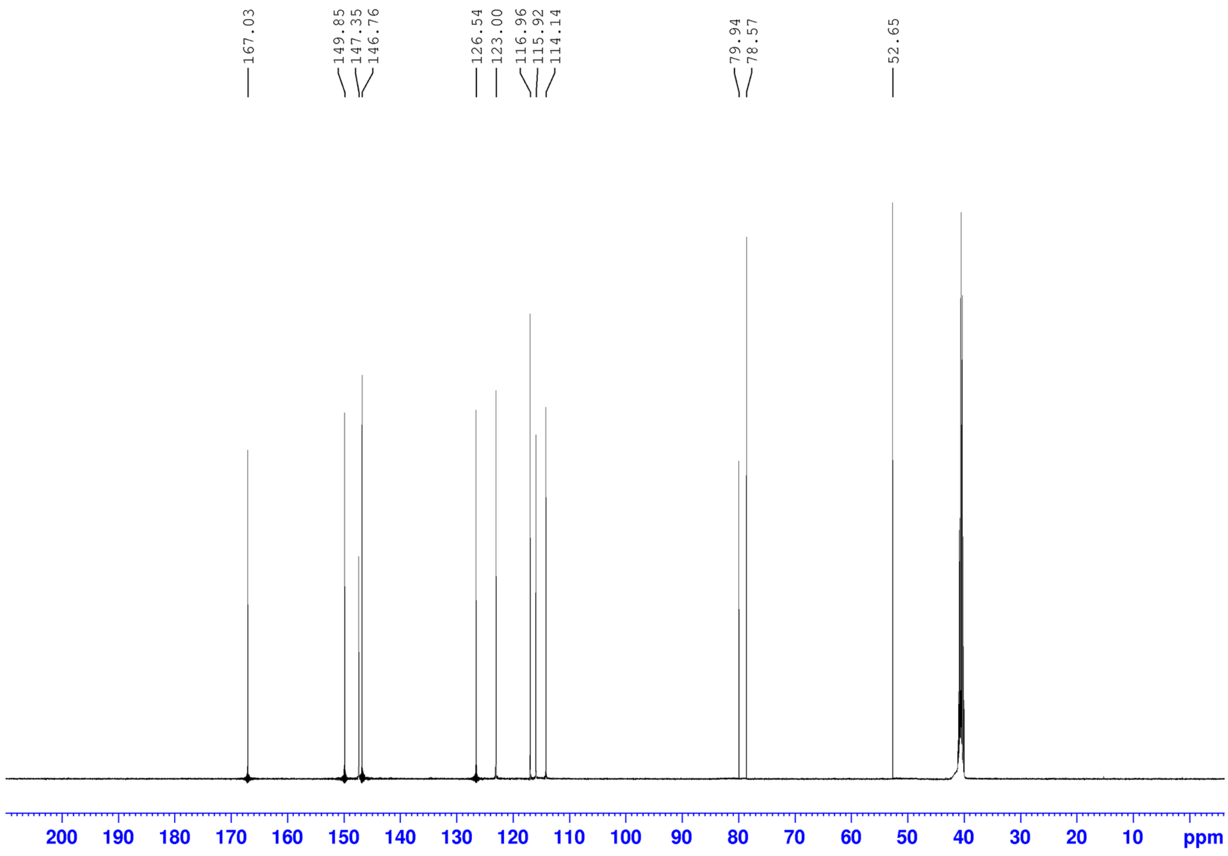

COSY NMR (300 MHz)

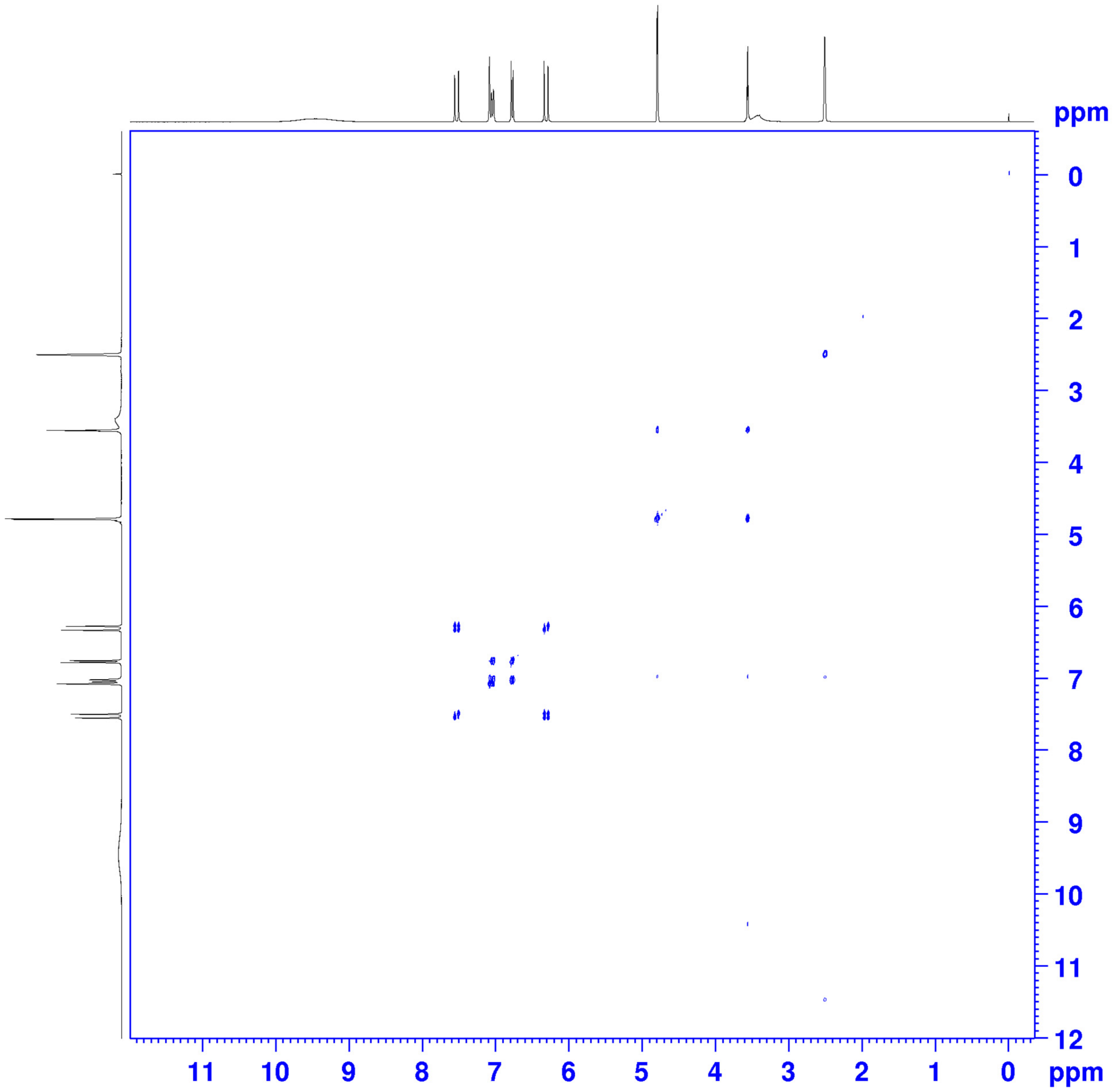

HRMS

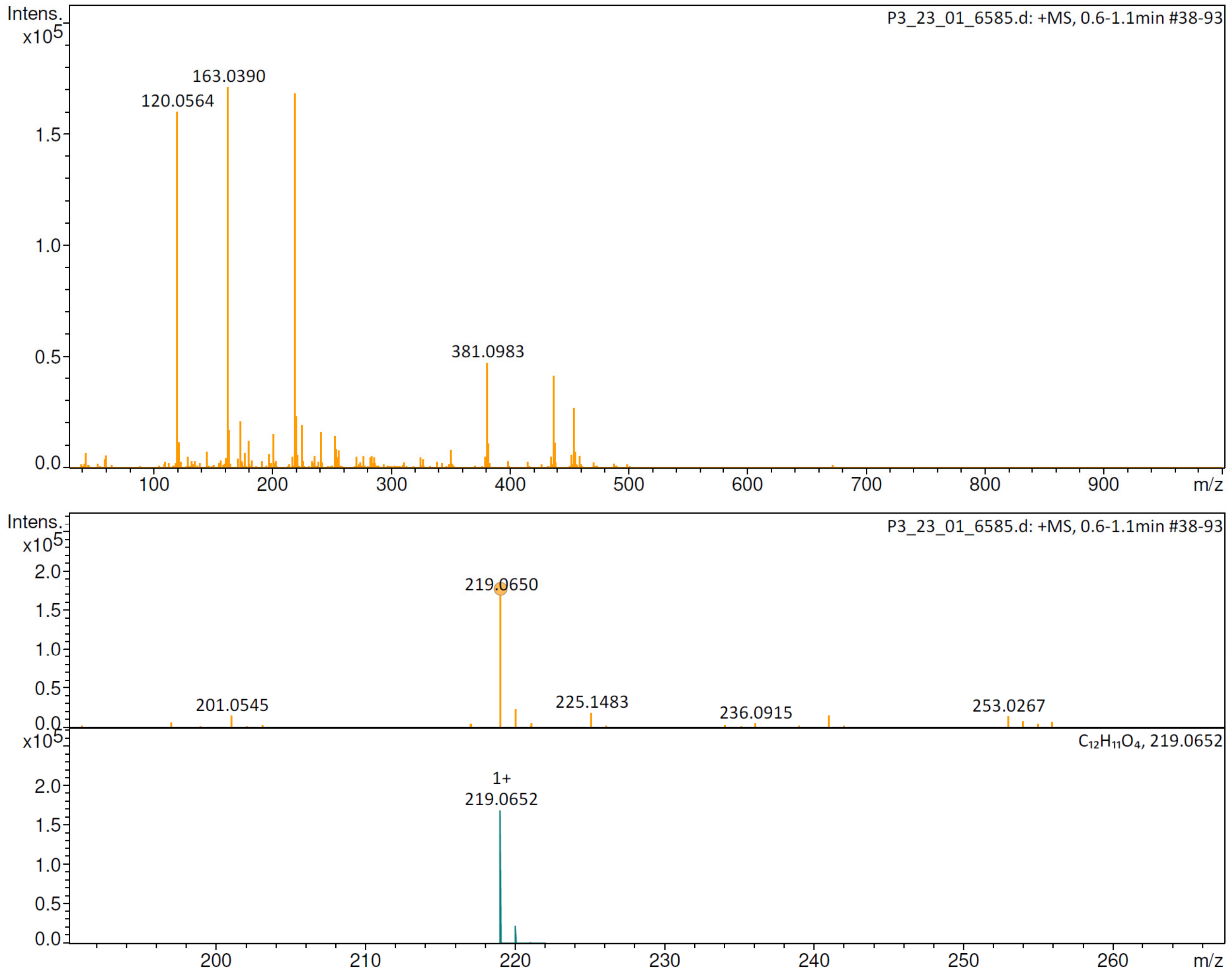

Intens.
x10^5
P3_23_01_6585.d: +MS, 0.6-1.1min #38-93
163.0390
120.0564
381.0983
m/z
Intens.
x10^5
P3_23_01_6585.d: +MS, 0.6-1.1min #38-93
219.0650
201.0545
225.1483
236.0915
253.0267
C12H11O4, 219.0652
1+
219.0652
m/z

^{1}H NMR (300 MHz) spectra of crude of the model reaction for synthesis of 23

- **Optimized reaction conditions**

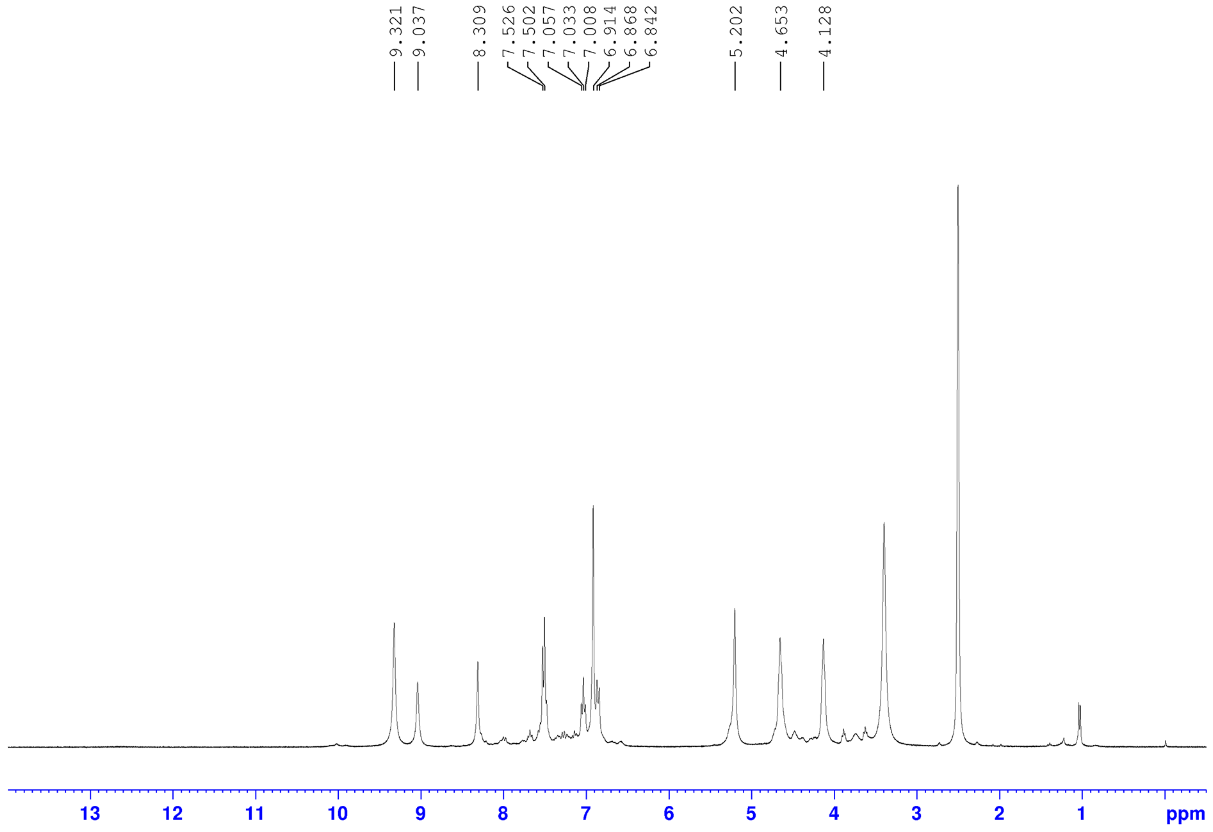

- **Gram-Scale reaction**

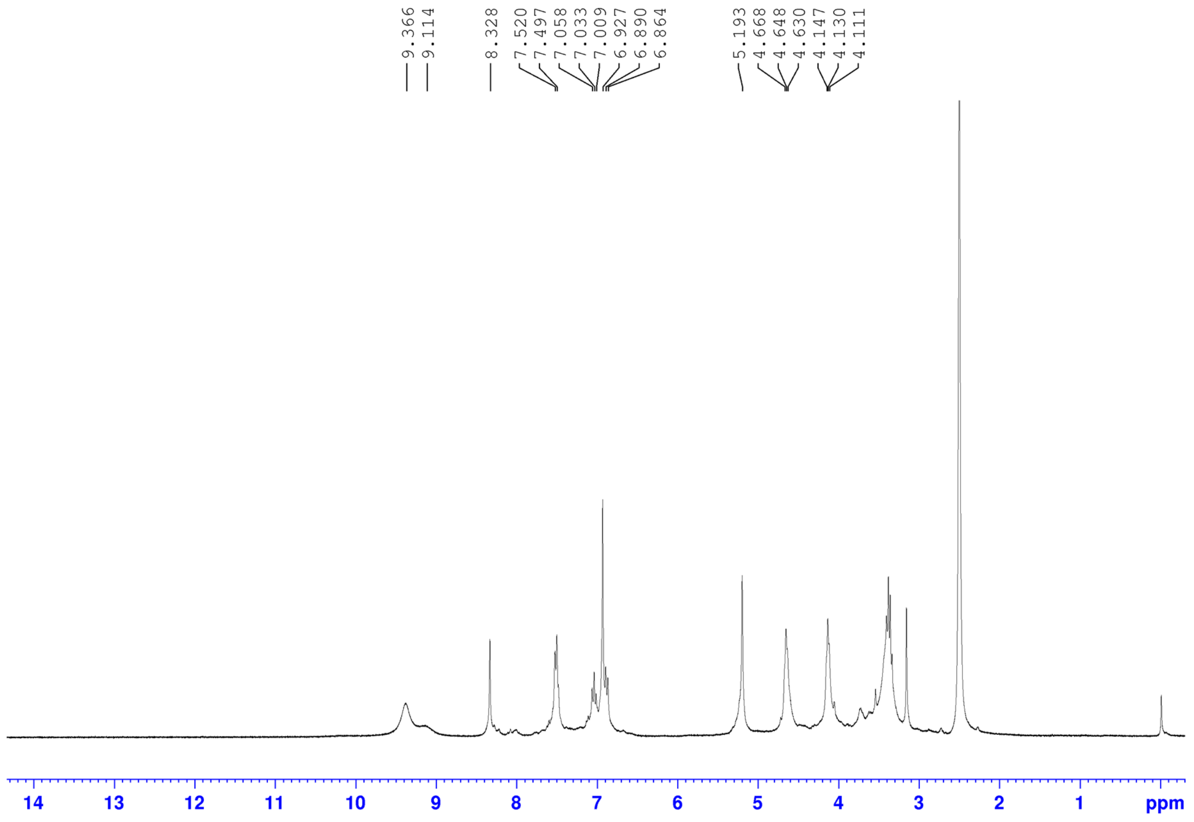

Characterization spectra of hybrid molecules 23-34

(1-(2-(isatin-1-yl)ethyl)-1,2,3-triazol-4-yl)methyl 3,4,5-trihydroxybenzoate (23)

^{1}H NMR (300 MHz)

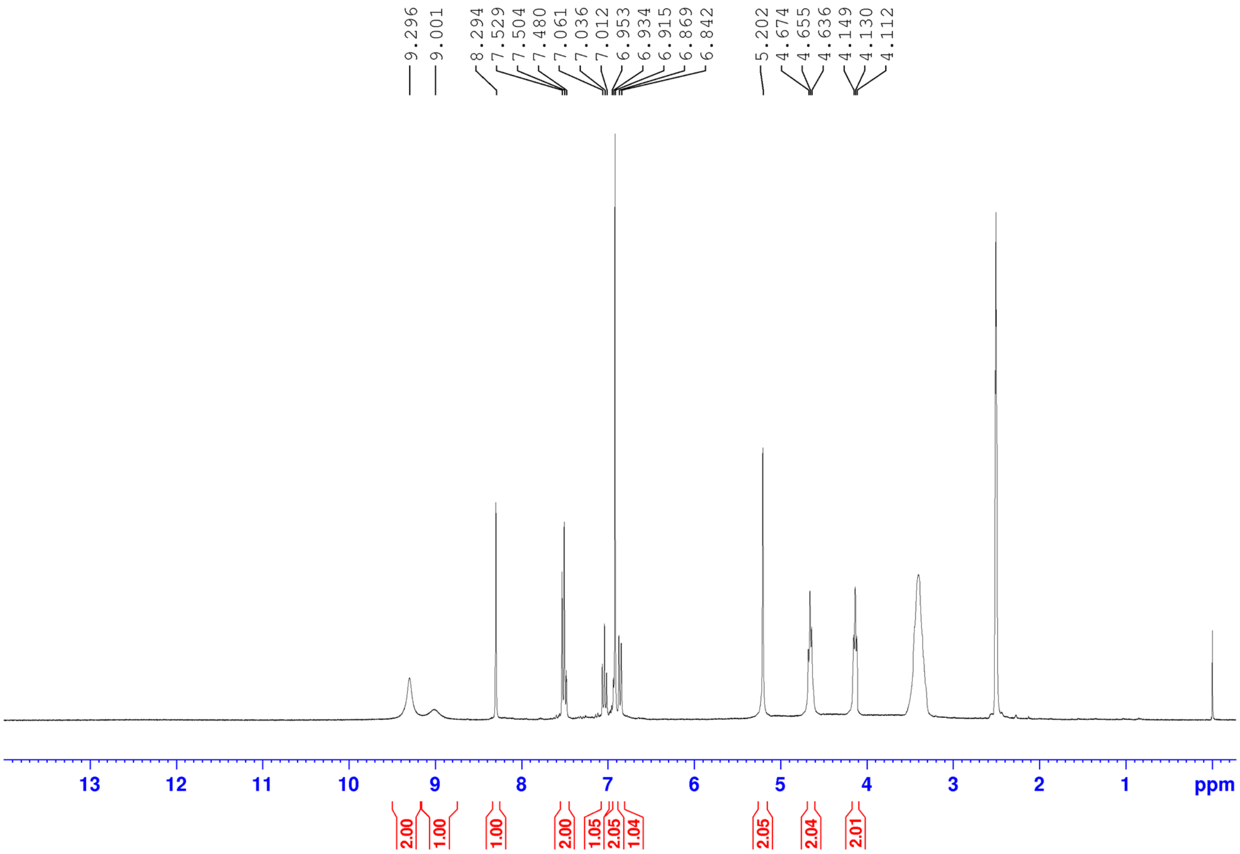

^{13}C NMR (125 MHz)

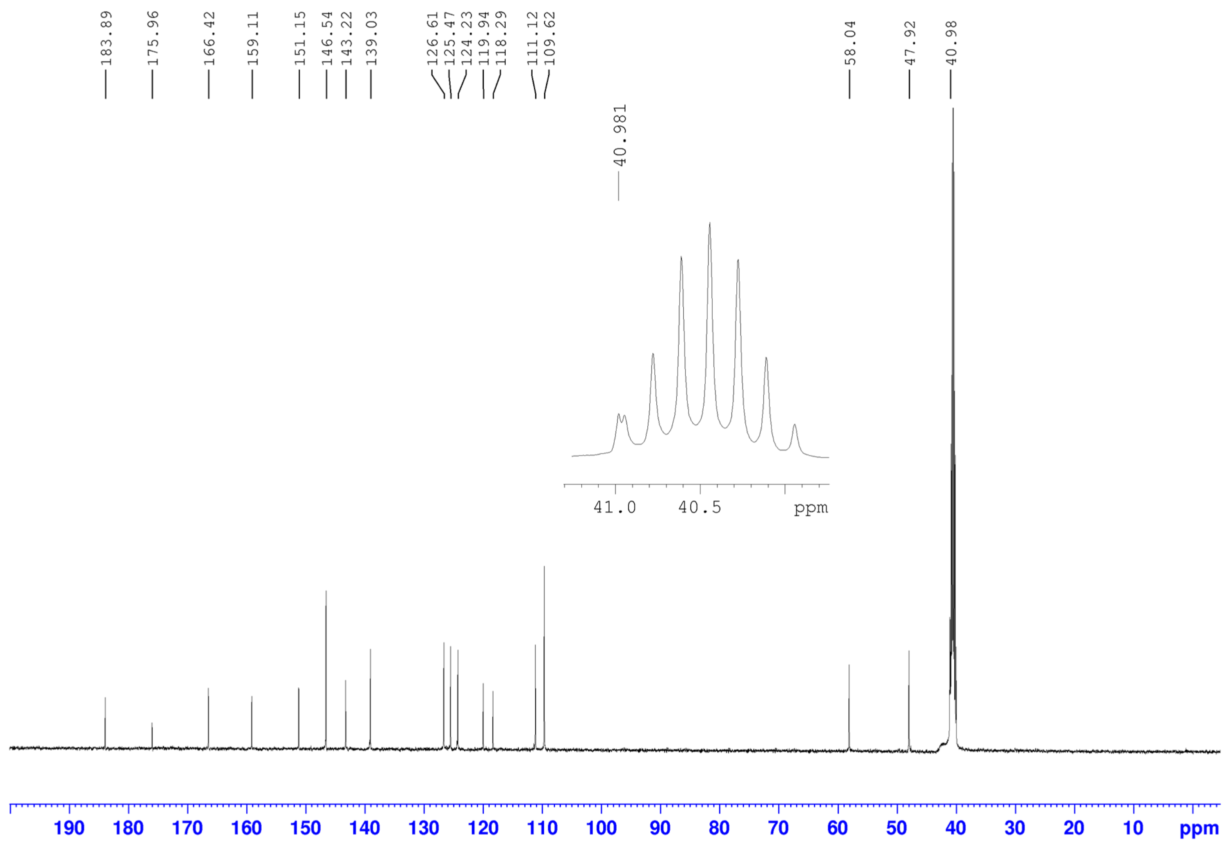

COSY NMR (300 MHz)

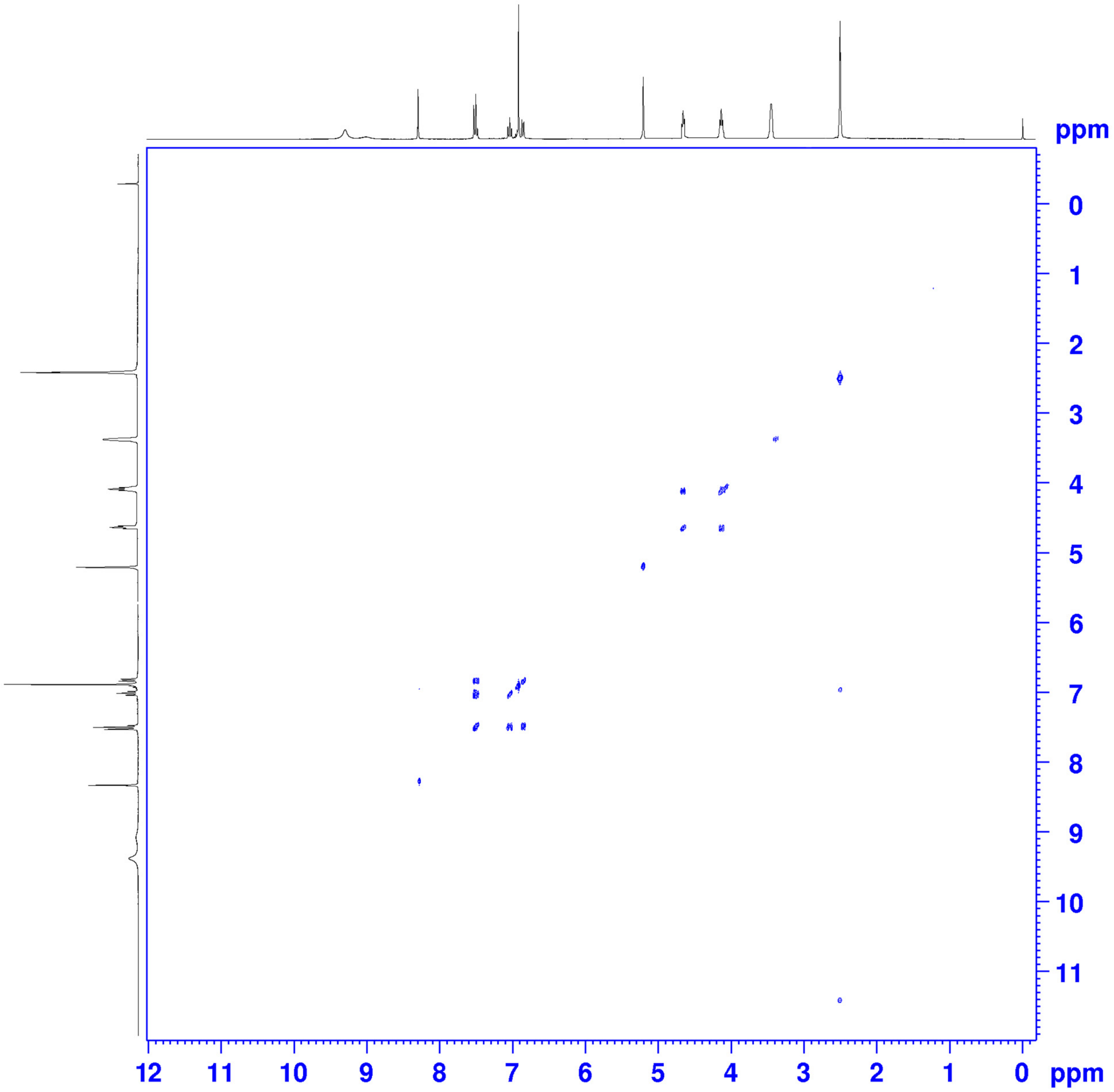

HRMS

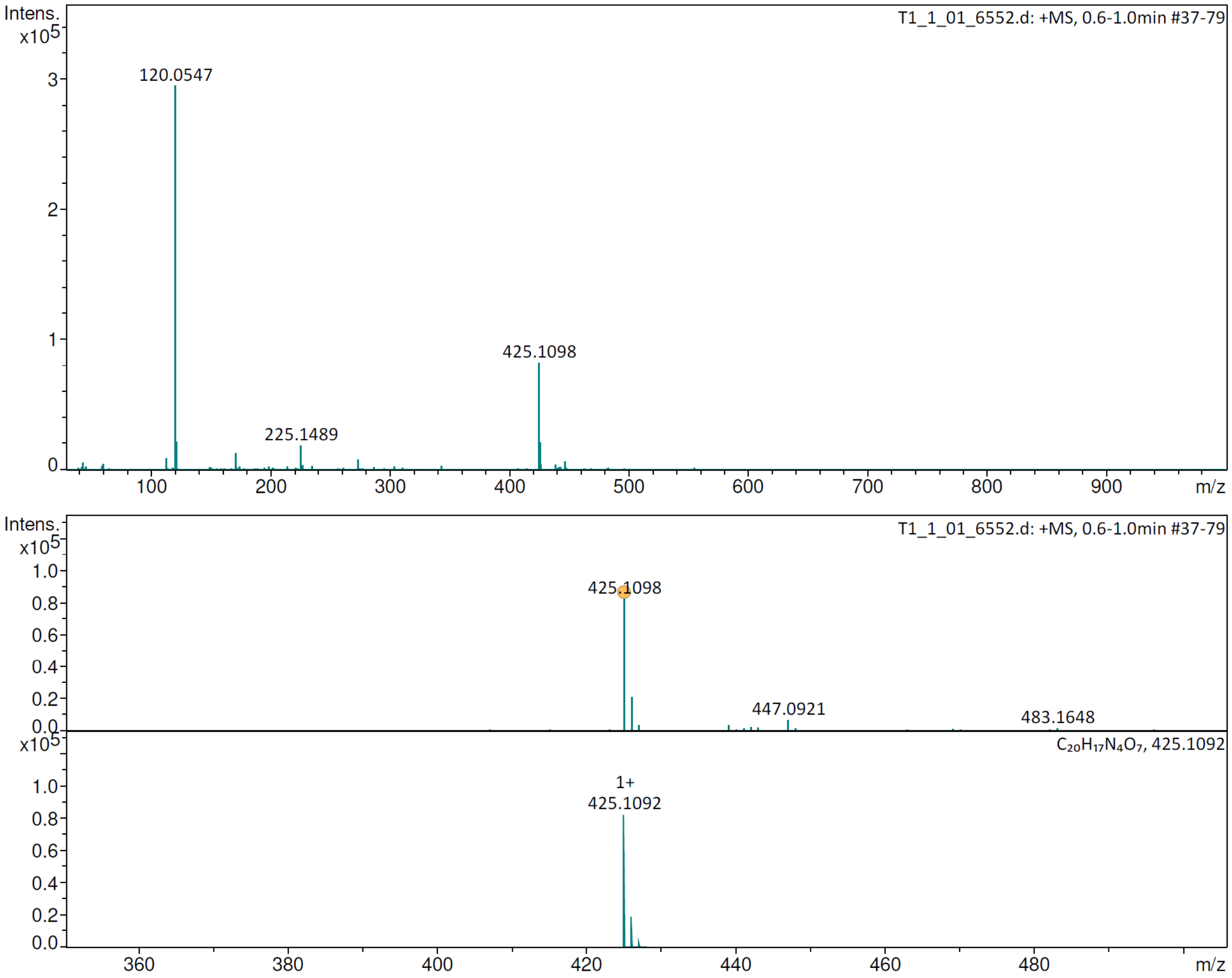
Intens.
x10^5
T1_1_01_6552.d: +MS, 0.6-1.0min #37-79
120.0547
425.1098
225.1489
m/z
Intens.
x10^5
T1_1_01_6552.d: +MS, 0.6-1.0min #37-79
425.1098
447.0921
483.1648
x10^5
C20H17N4O7, 425.1092
1+
425.1092
m/z

(1-(2-(5-methoxy-isatin-1-yl)ethyl)-1,2,3-triazol-4-yl)methyl 3,4,5-trihydroxybenzoate (24)

^{1}H NMR (300 MHz)

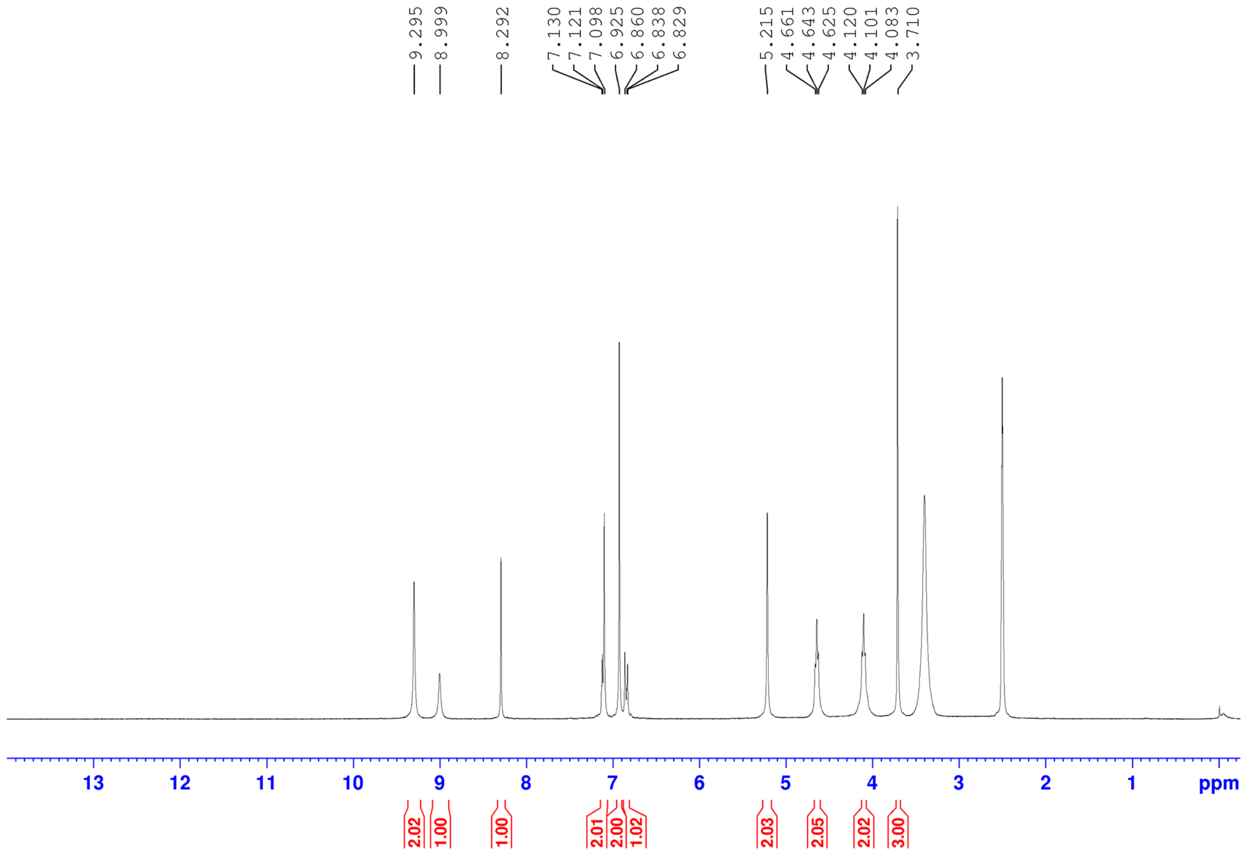

^{13}C NMR (125 MHz)

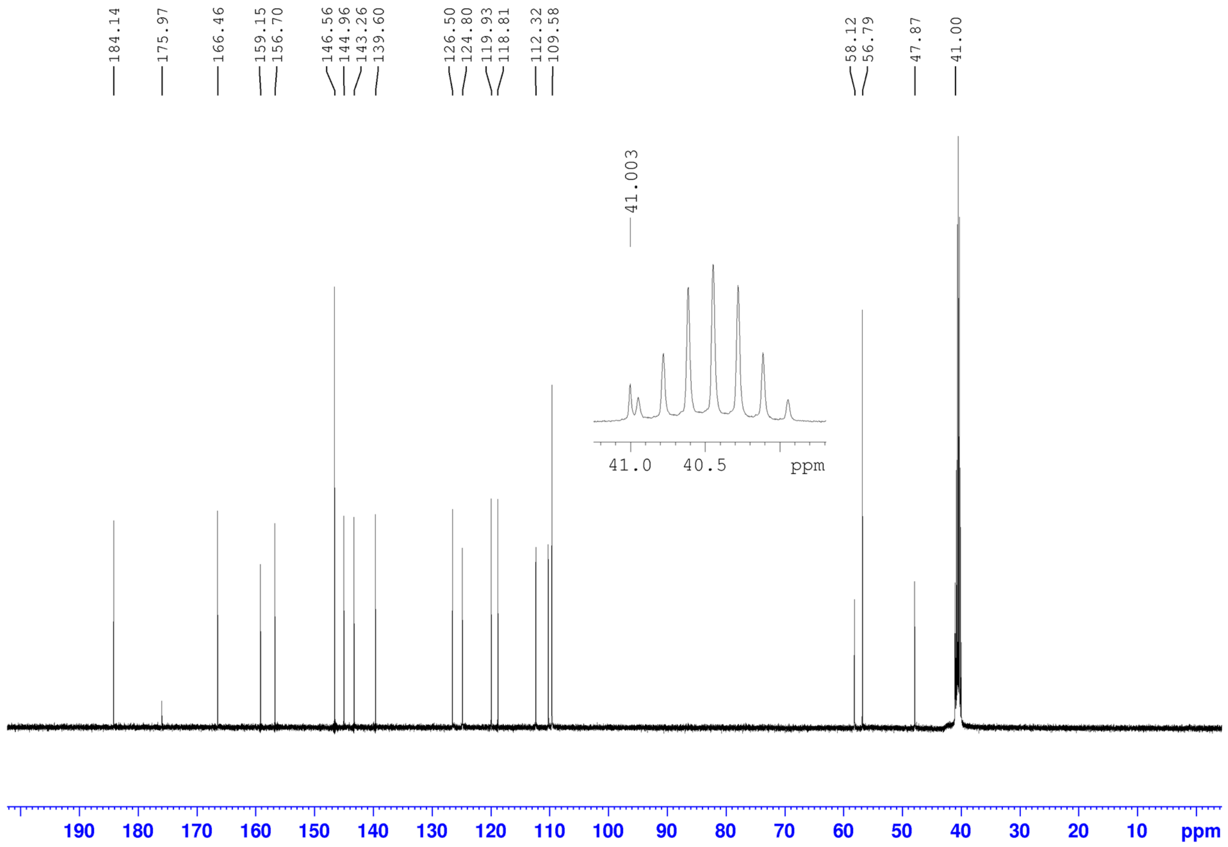

COSY NMR (300 MHz)

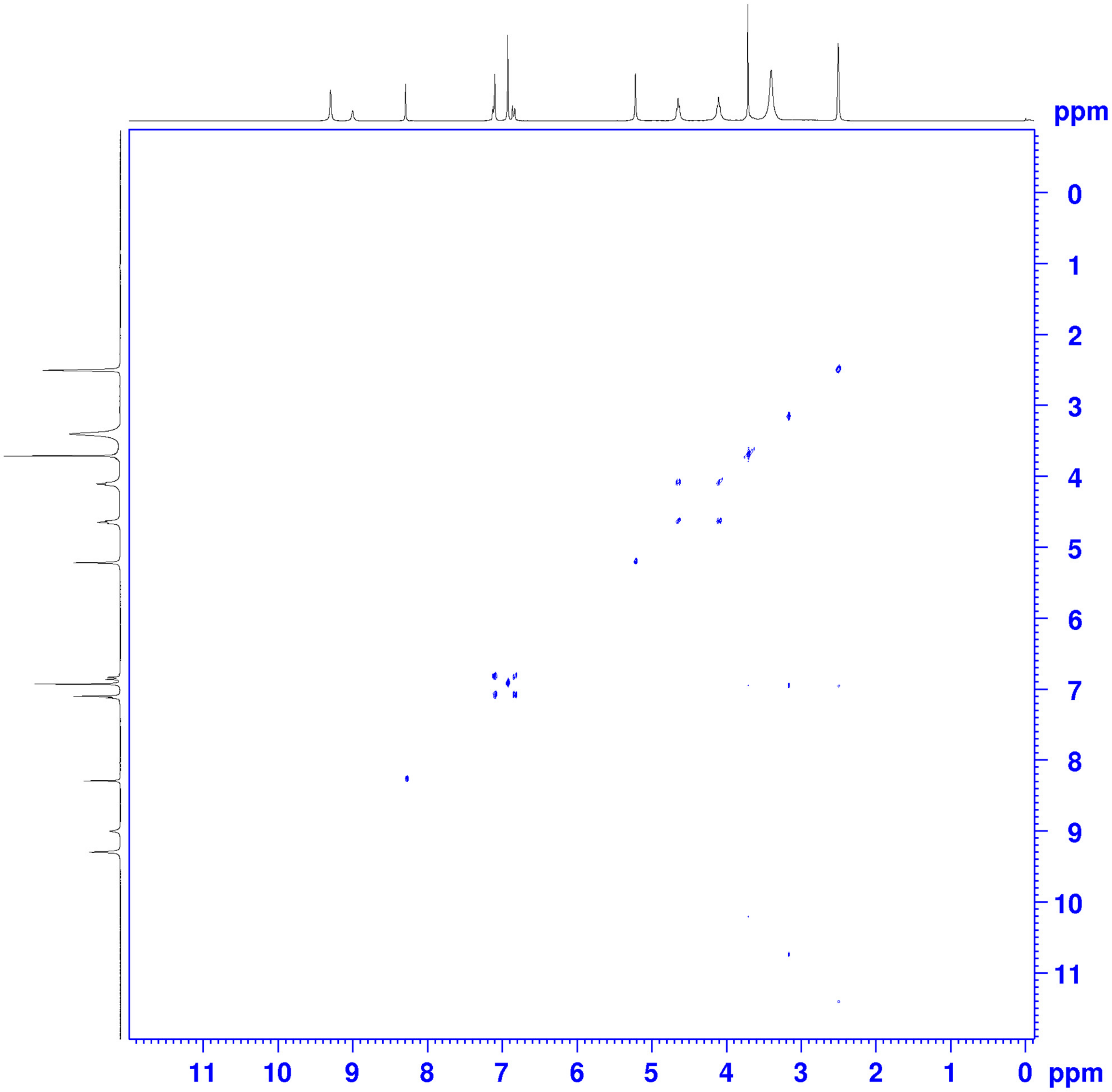

HRMS

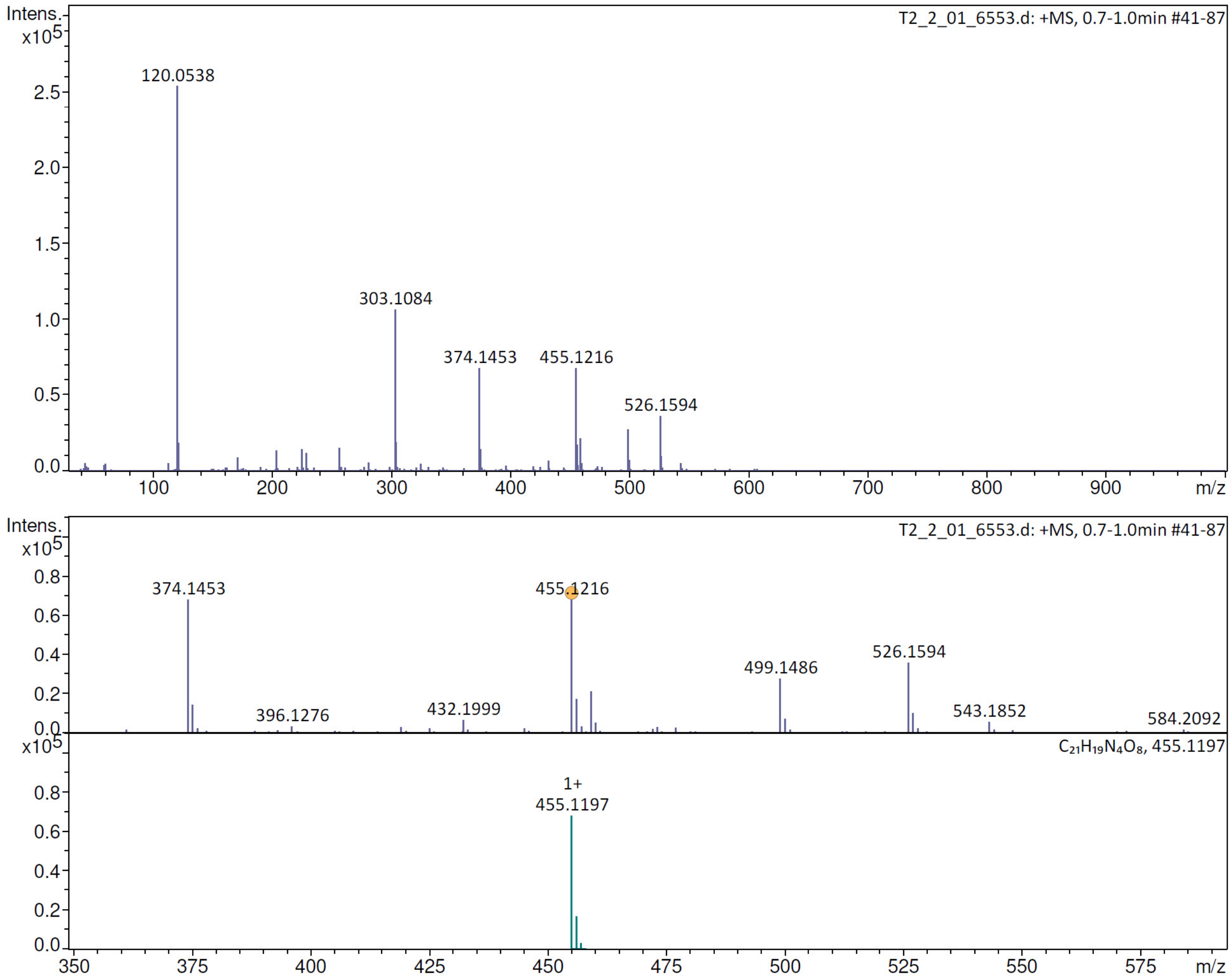

Intens.
x10^5
T2_2_01_6553.d: +MS, 0.7-1.0min #41-87
120.0538
303.1084
374.1453
455.1216
526.1594
m/z
374.1453
455.1216
396.1276
432.1999
499.1486
526.1594
543.1852
584.2092
C21H19N4O8, 455.1197
1+
455.1197

(1-(2-(5-nitro-isatin-1-yl)ethyl)-1,2,3-triazol-4-yl)methyl 3,4,5-trihydroxybenzoate (25)

^{1}H NMR (300 MHz)

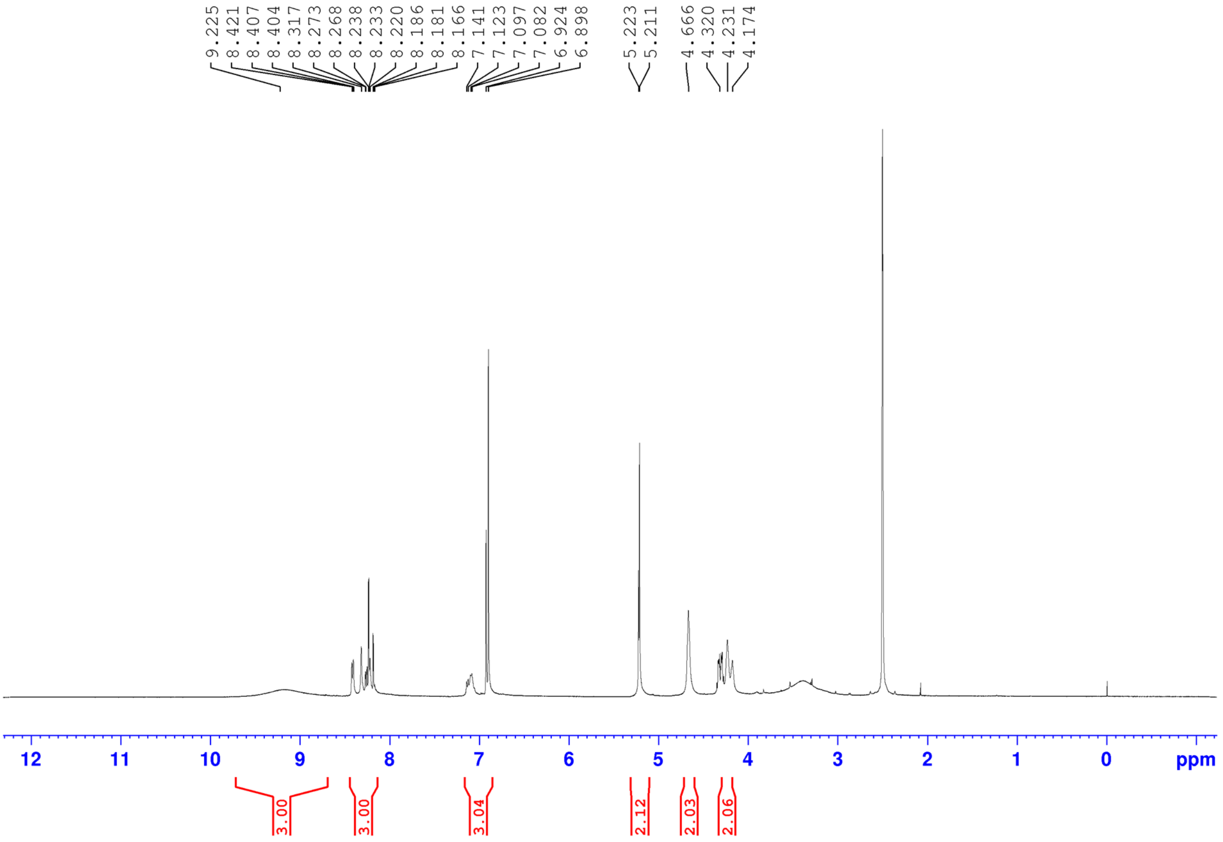

^{13}C NMR (125 MHz)

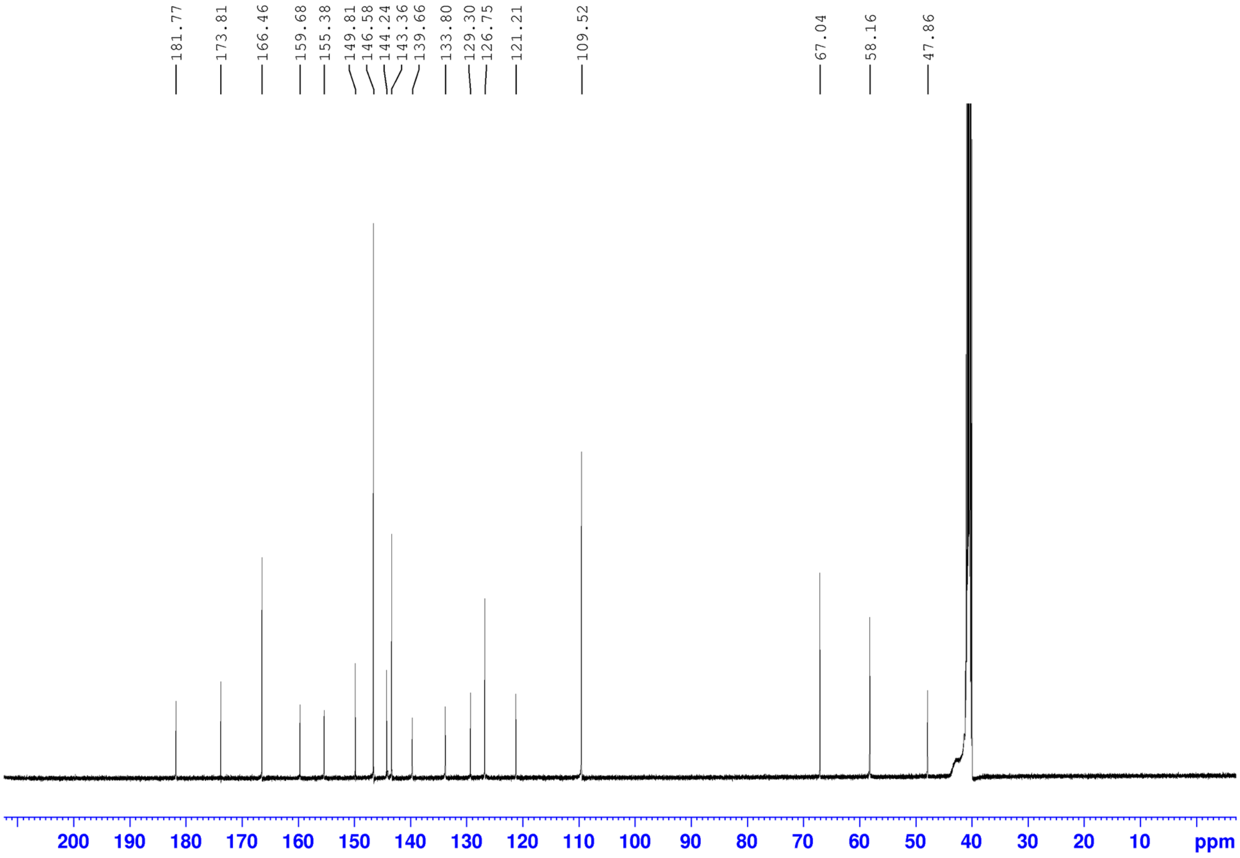

COSY NMR (300 MHz)

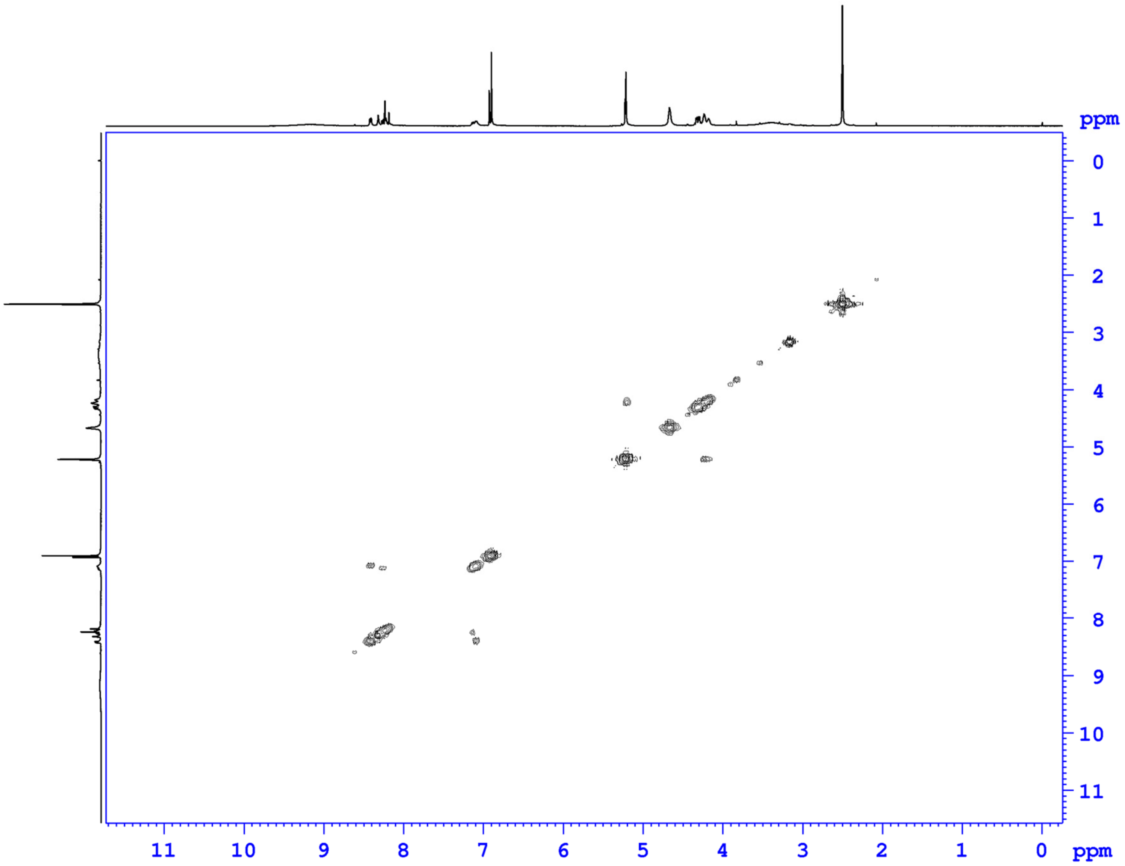

(1-(2-(5-chloro-isatin-1-yl)ethyl)-1,2,3-triazol-4-yl)methyl 3,4,5-trihydroxybenzoate (26)

^{1}H NMR (300 MHz)

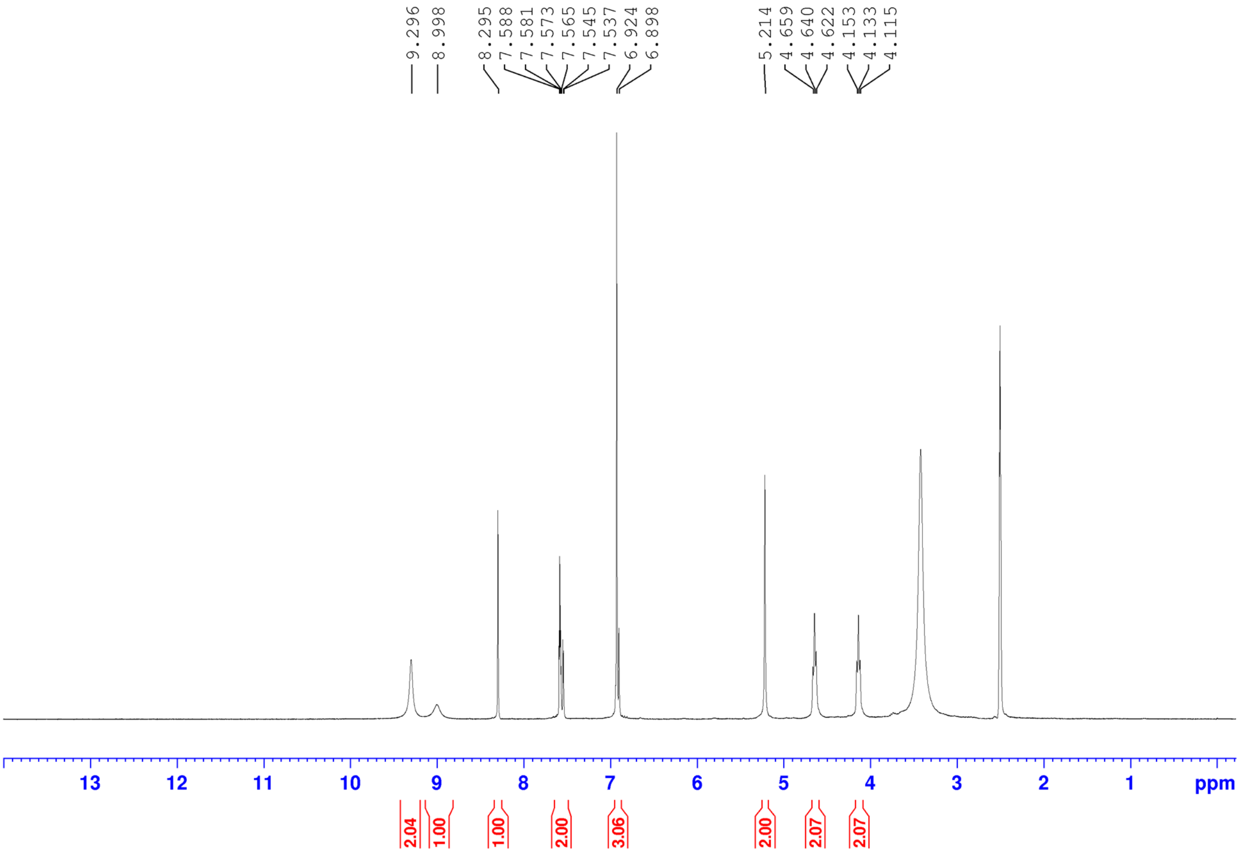

^{13}C NMR (125 MHz)

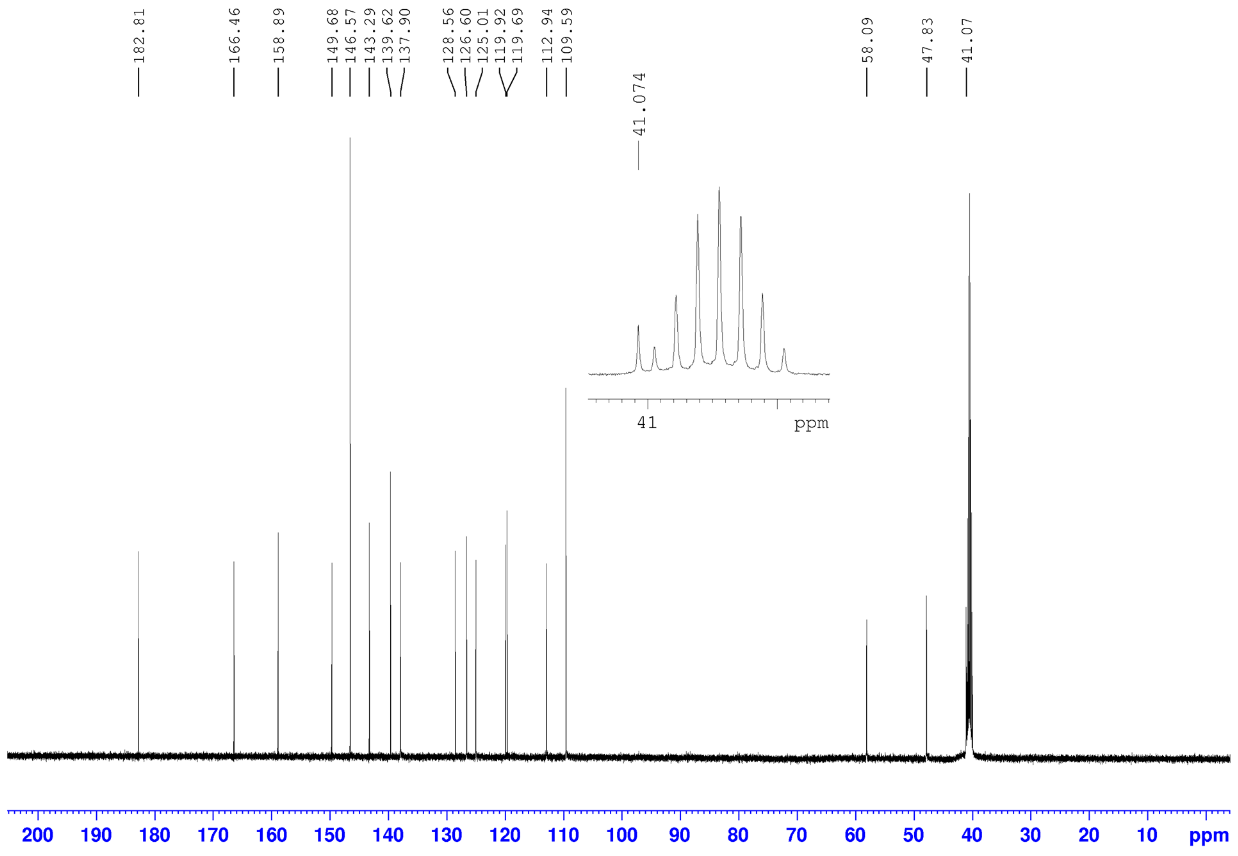

COSY NMR (300 MHz)

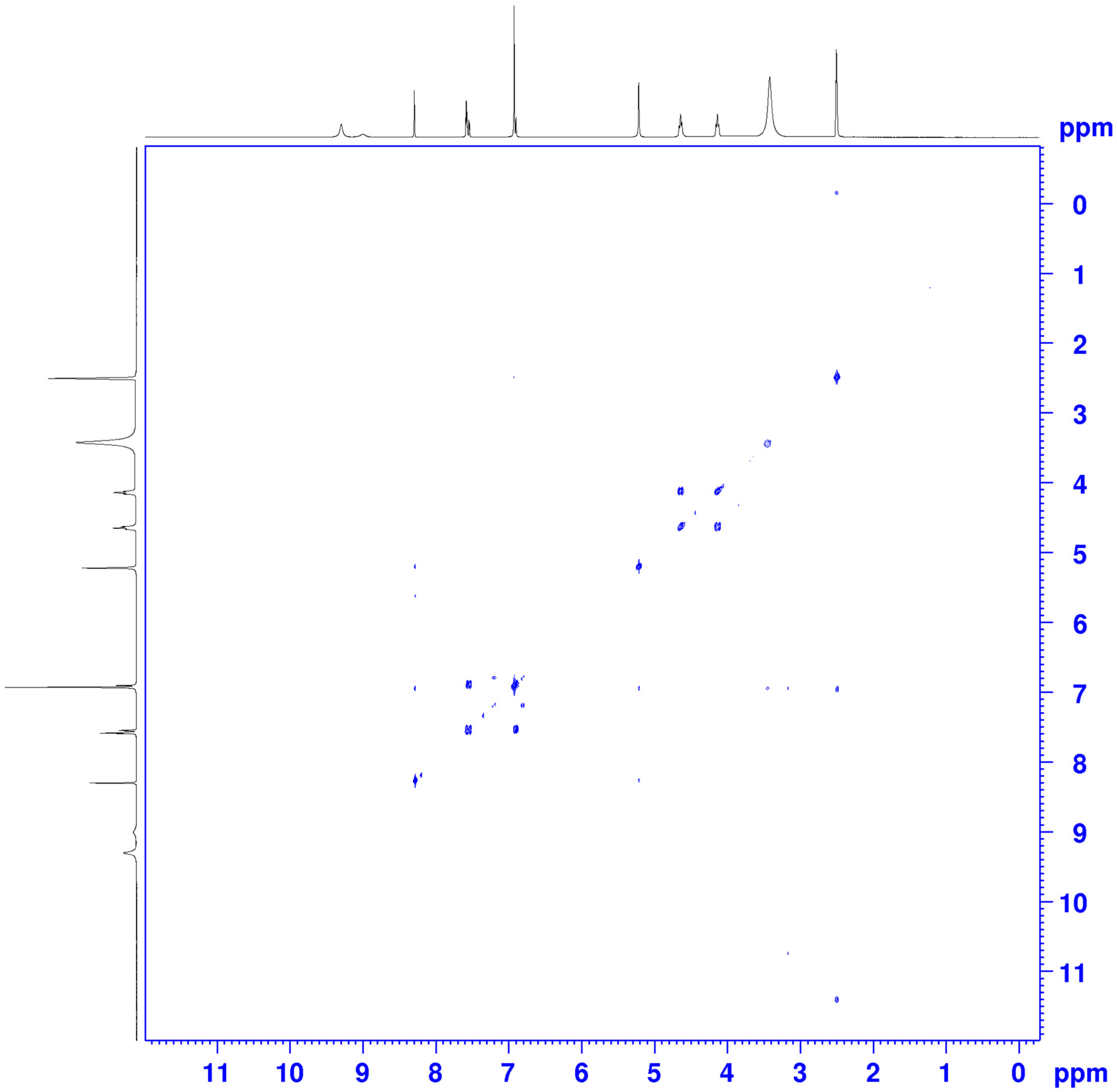

HRMS

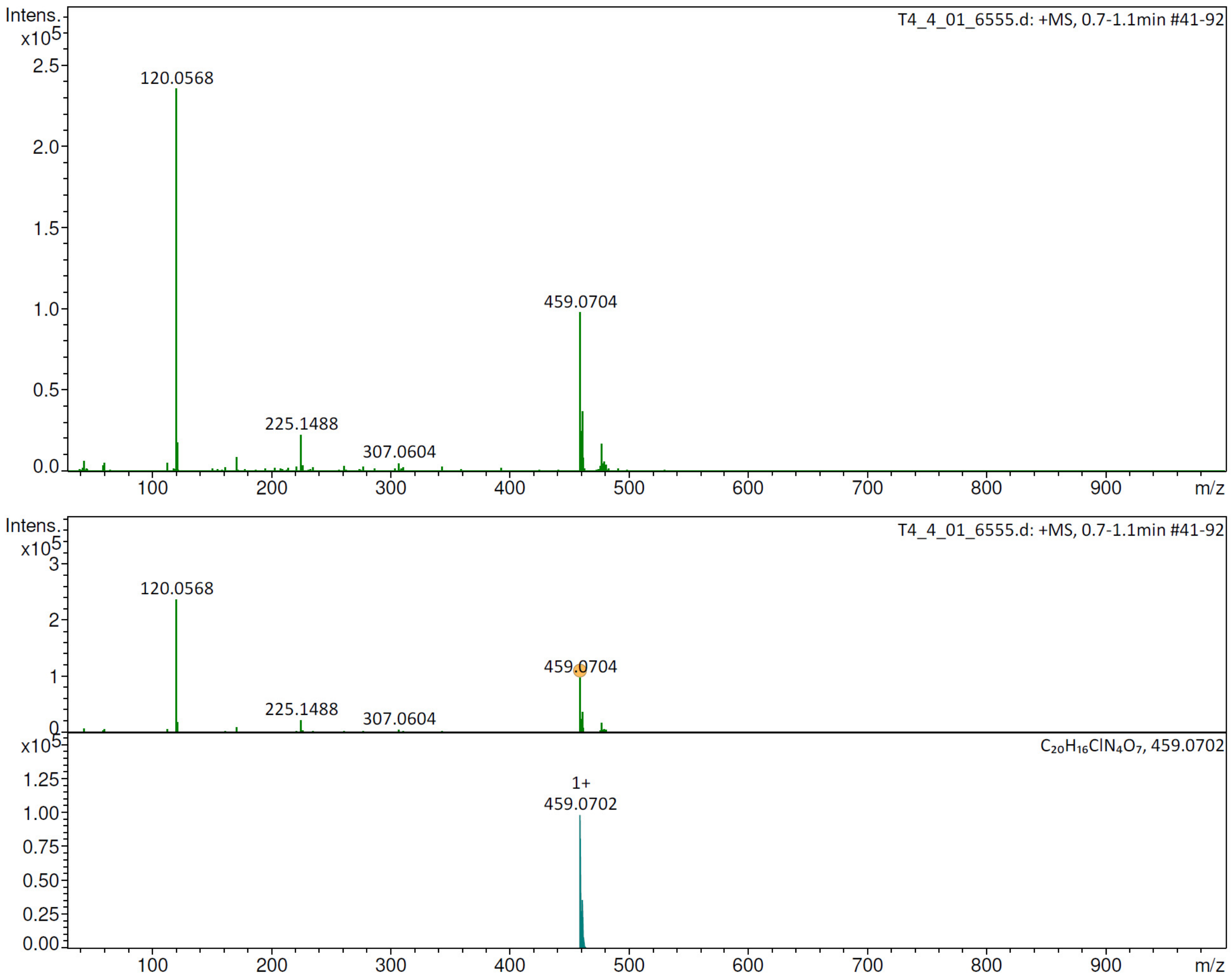

Intens.
x10^5
T4_4_01_6555.d: +MS, 0.7-1.1min #41-92
120.0568
459.0704
225.1488
307.0604
m/z
C20H16ClN4O7, 459.0702
1+
459.0702

(1-(2-(isatin-1-yl)ethyl)-1,2,3-triazol-4-yl)methyl 4-hydroxybenzoate (27)

^{1}H NMR (300 MHz)

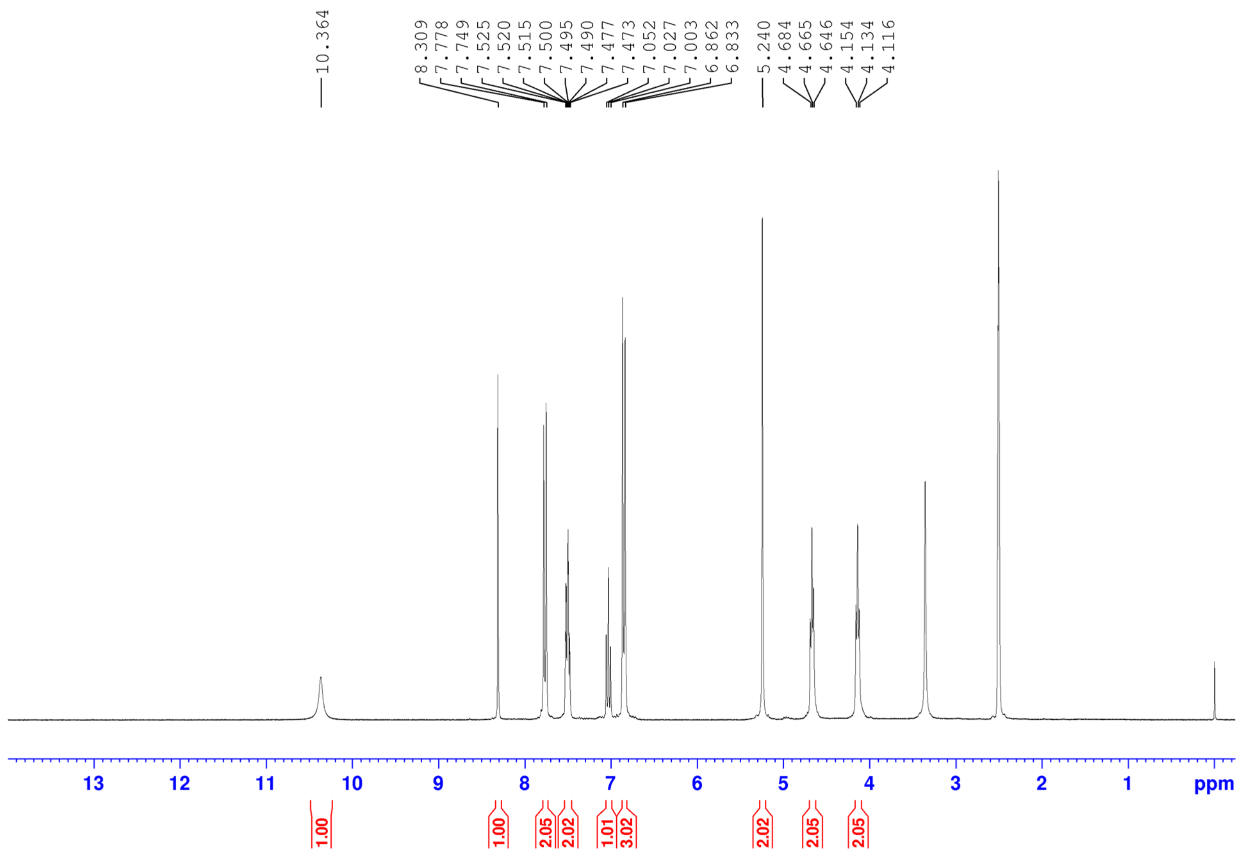

^{13}C NMR (125 MHz)

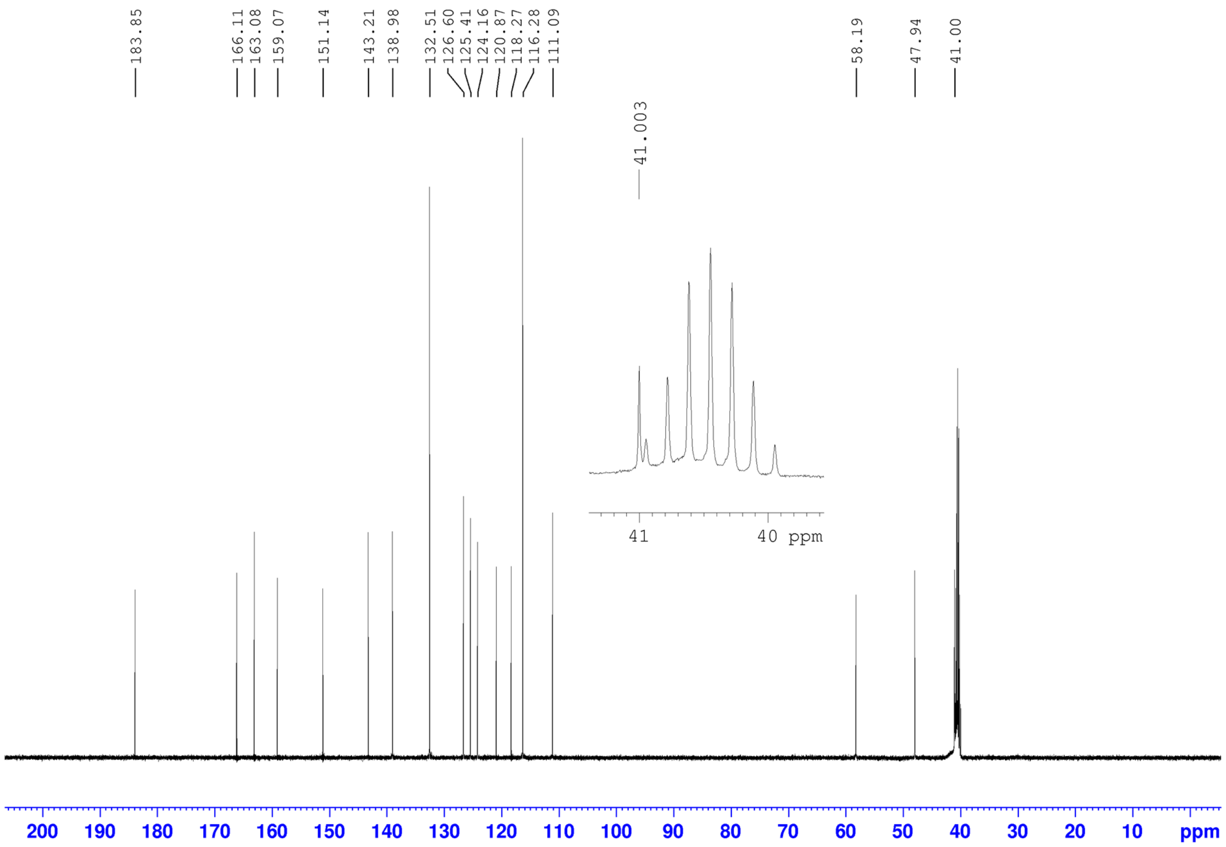

COSY NMR (300 MHz)

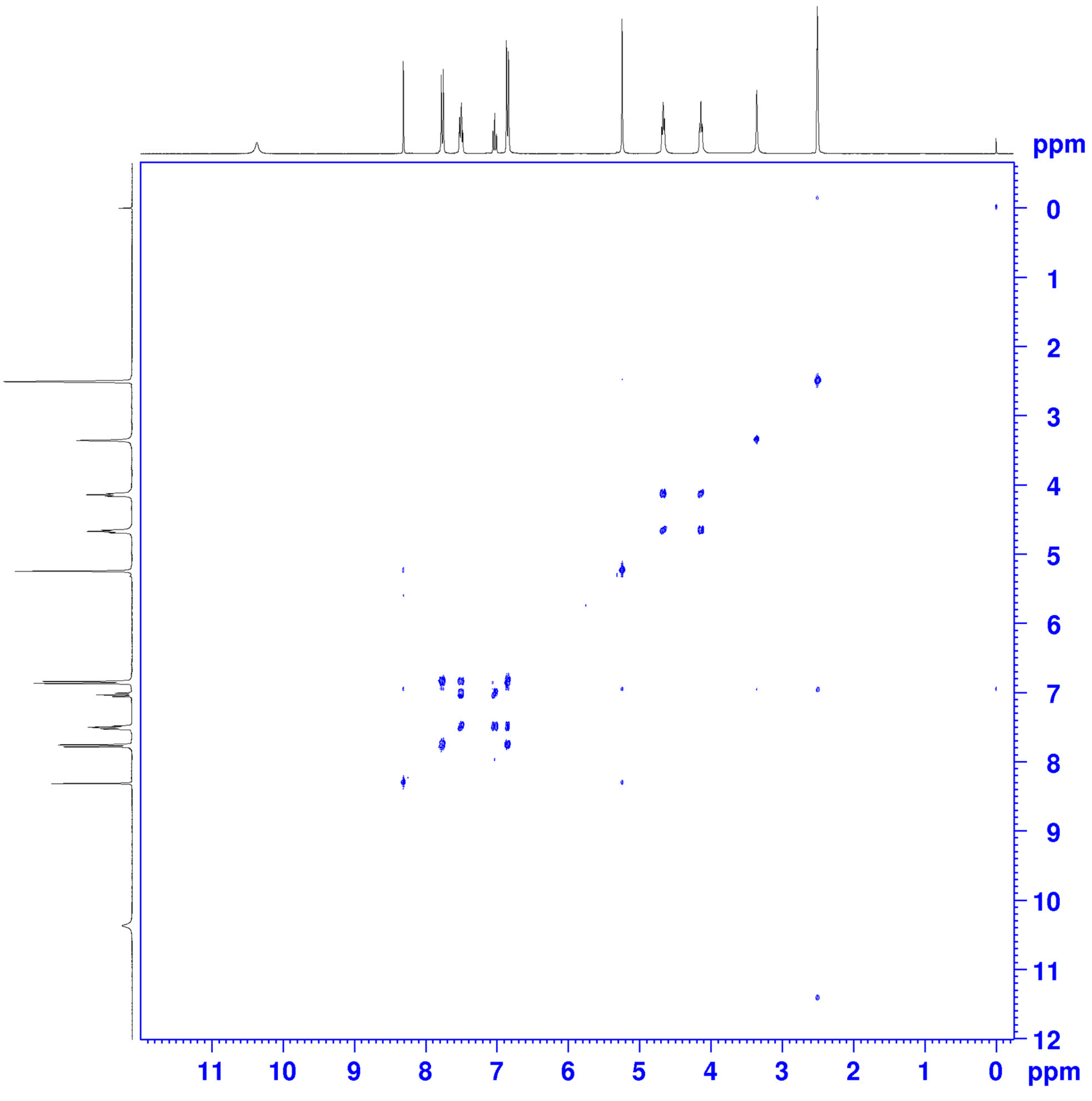

HRMS

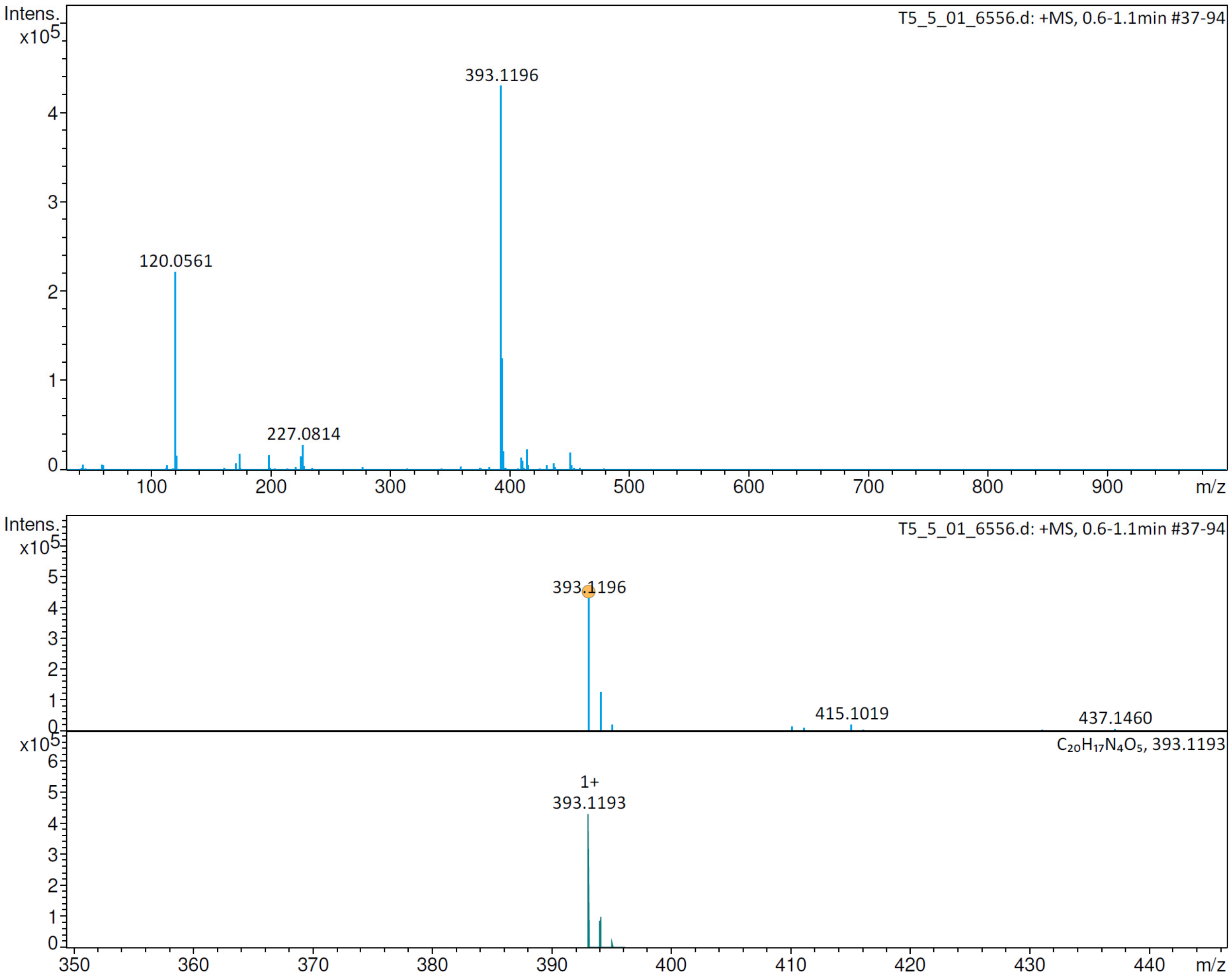

Intens.
x10^5
T5_5_01_6556.d: +MS, 0.6-1.1min #37-94
393.1196
120.0561
227.0814
m/z
393.1196
415.1019
437.1460
C20H17N4O5, 393.1193
1+
393.1193

(1-(2-(5-methoxy-isatin-1-yl)ethyl)-1,2,3-triazol-4-yl)methyl 4-hydroxybenzoate (28)

^{1}H NMR (300 MHz)

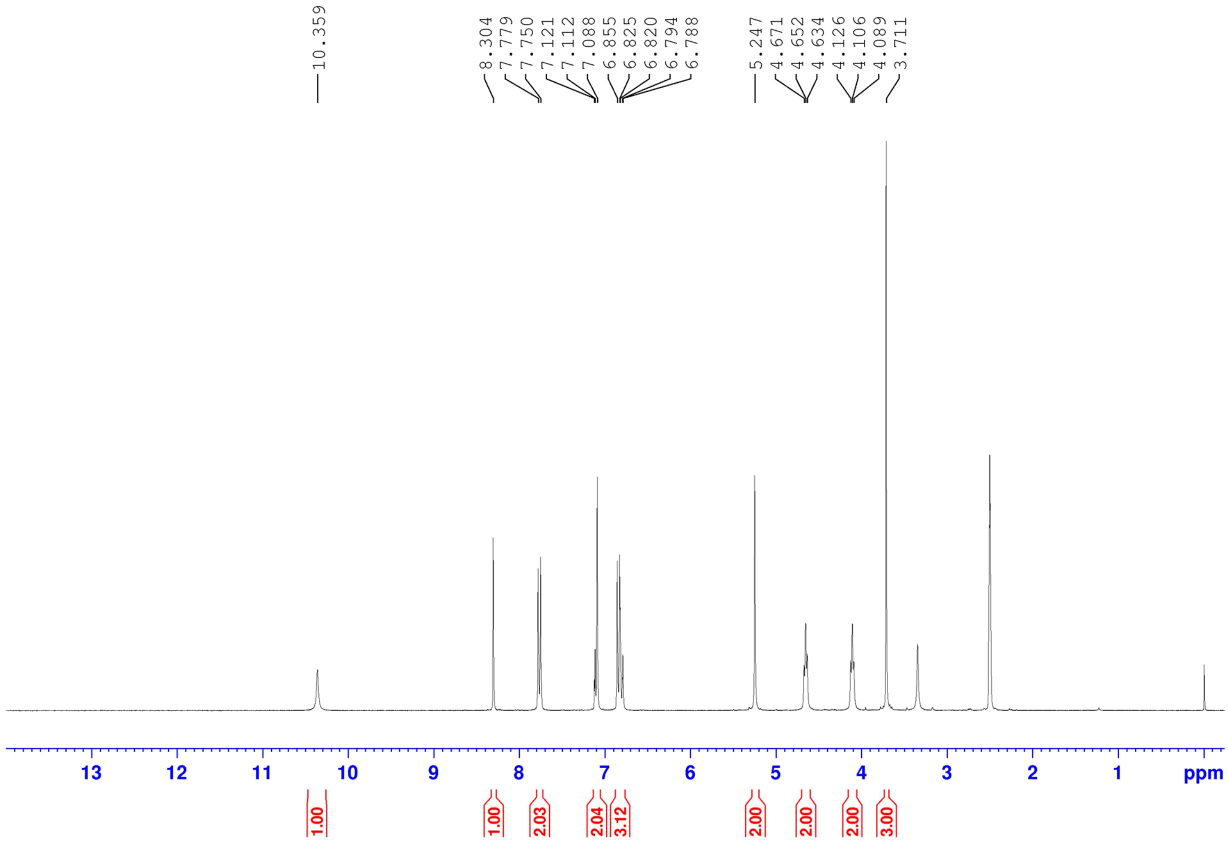

^{13}C NMR (125 MHz)

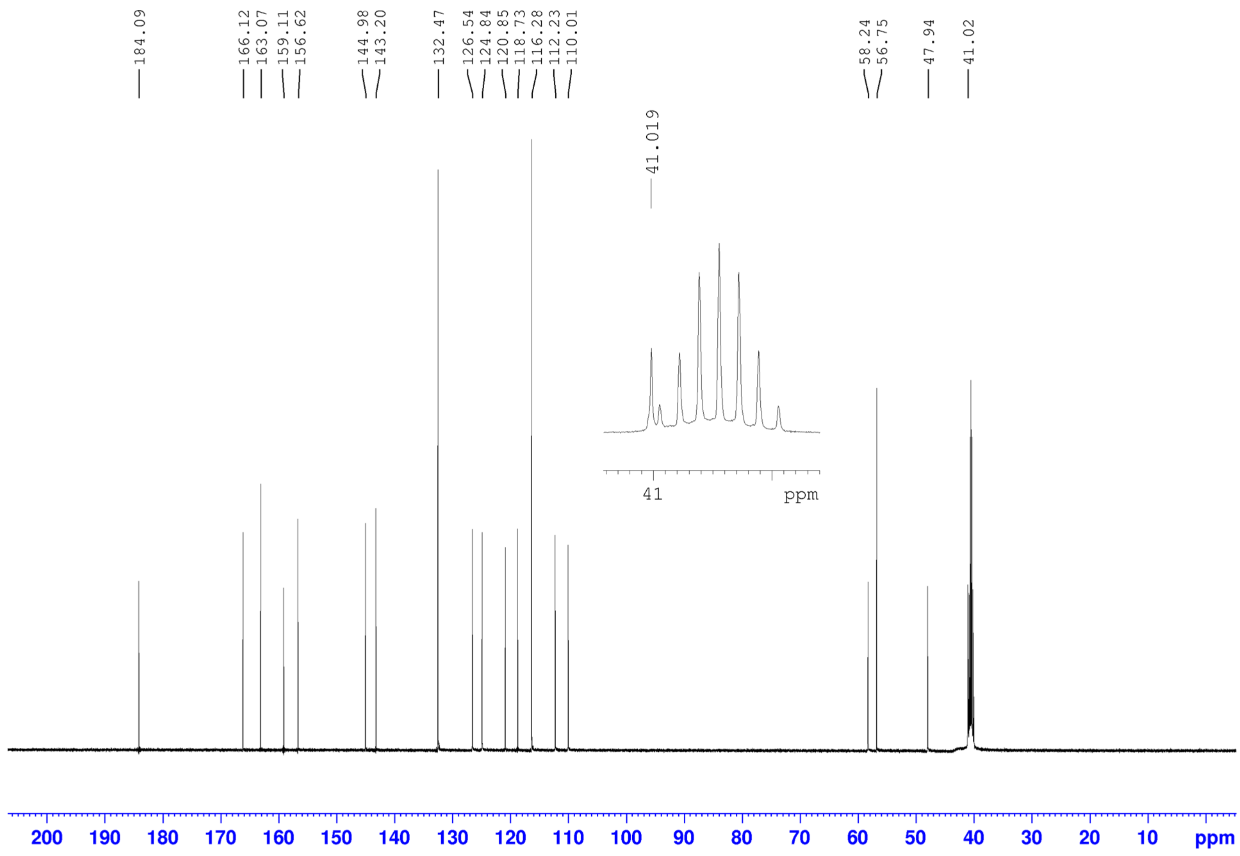

COSY NMR (300 MHz)

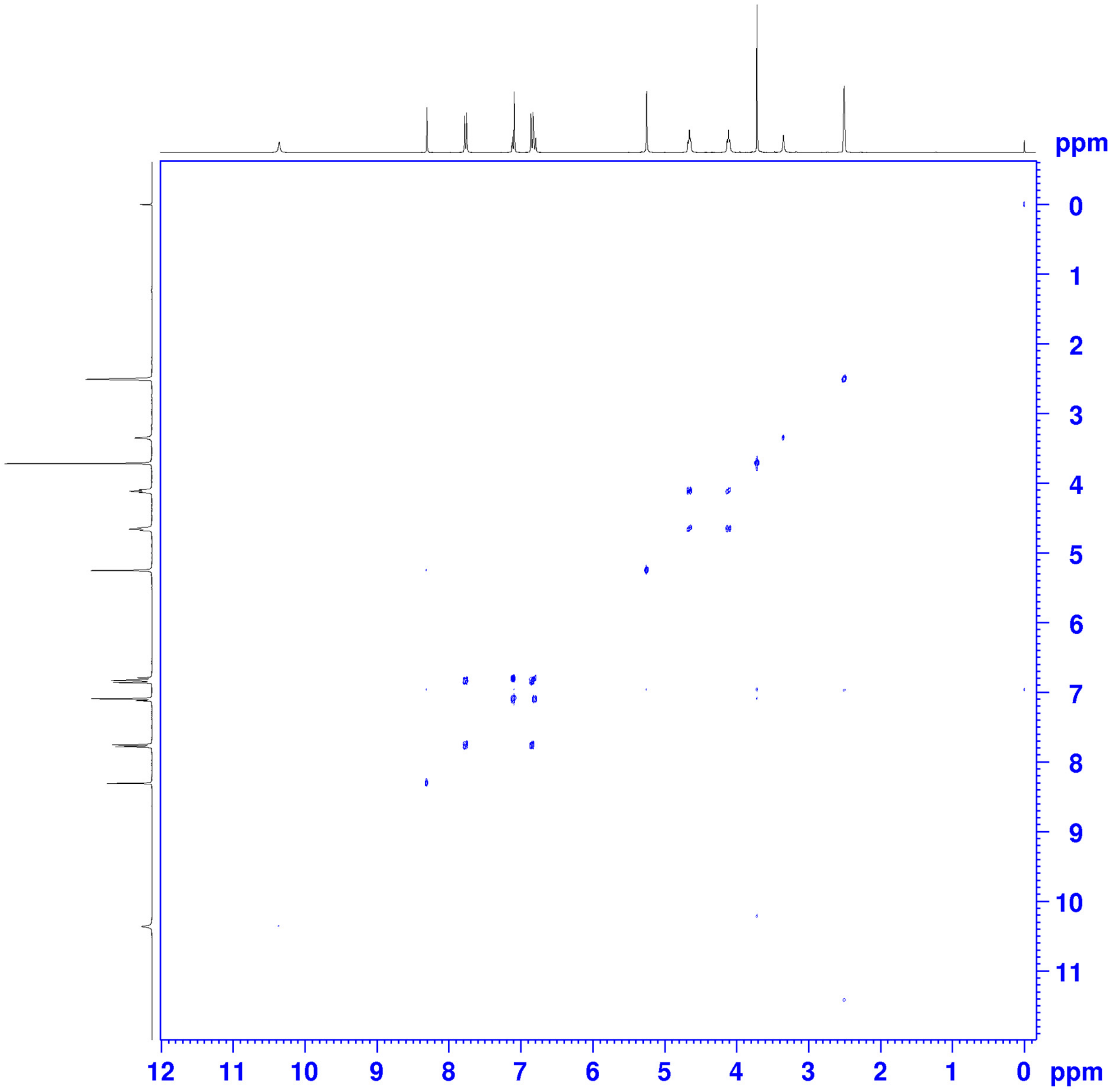

HRMS

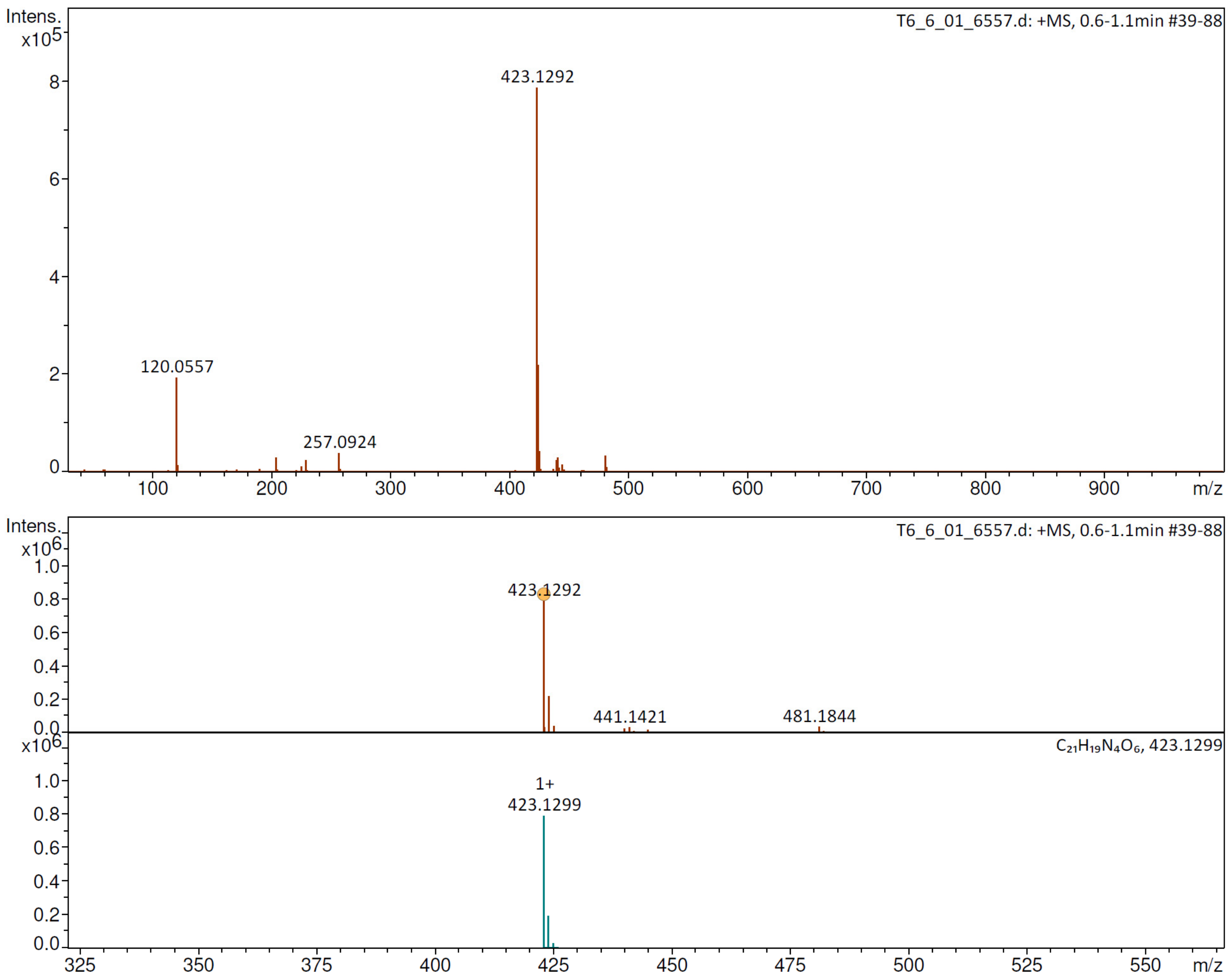

Intens.
x10^5
T6_6_01_6557.d: +MS, 0.6-1.1min #39-88
423.1292
120.0557
257.0924
m/z
Intens.
x10^6
T6_6_01_6557.d: +MS, 0.6-1.1min #39-88
423.1292
441.1421
481.1844
C21H19N4O6, 423.1299
1+
423.1299
m/z

(1-(2-(5-nitro-isatin-1-yl)ethyl)-1,2,3-triazol-4-yl)methyl 4-hydroxybenzoate (29)

^{1}H NMR (500 MHz)

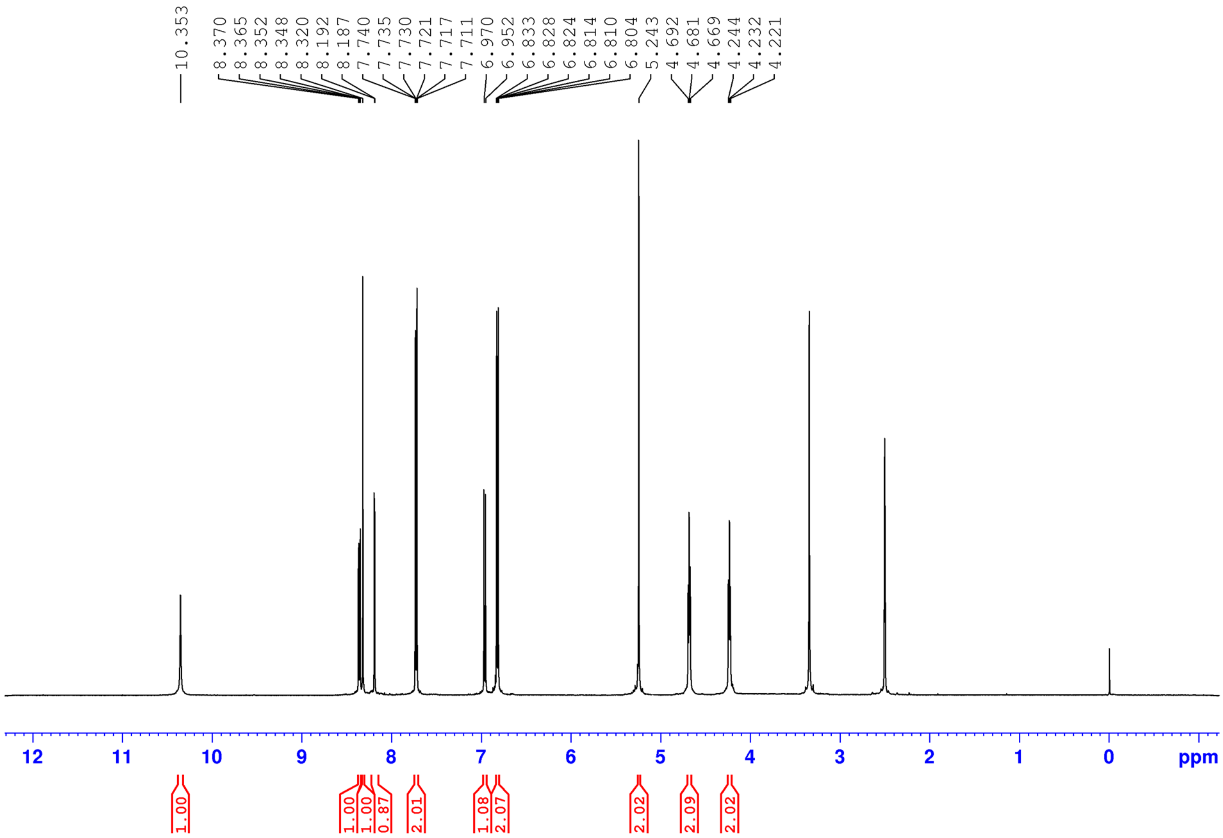

^{13}C NMR (125 MHz)

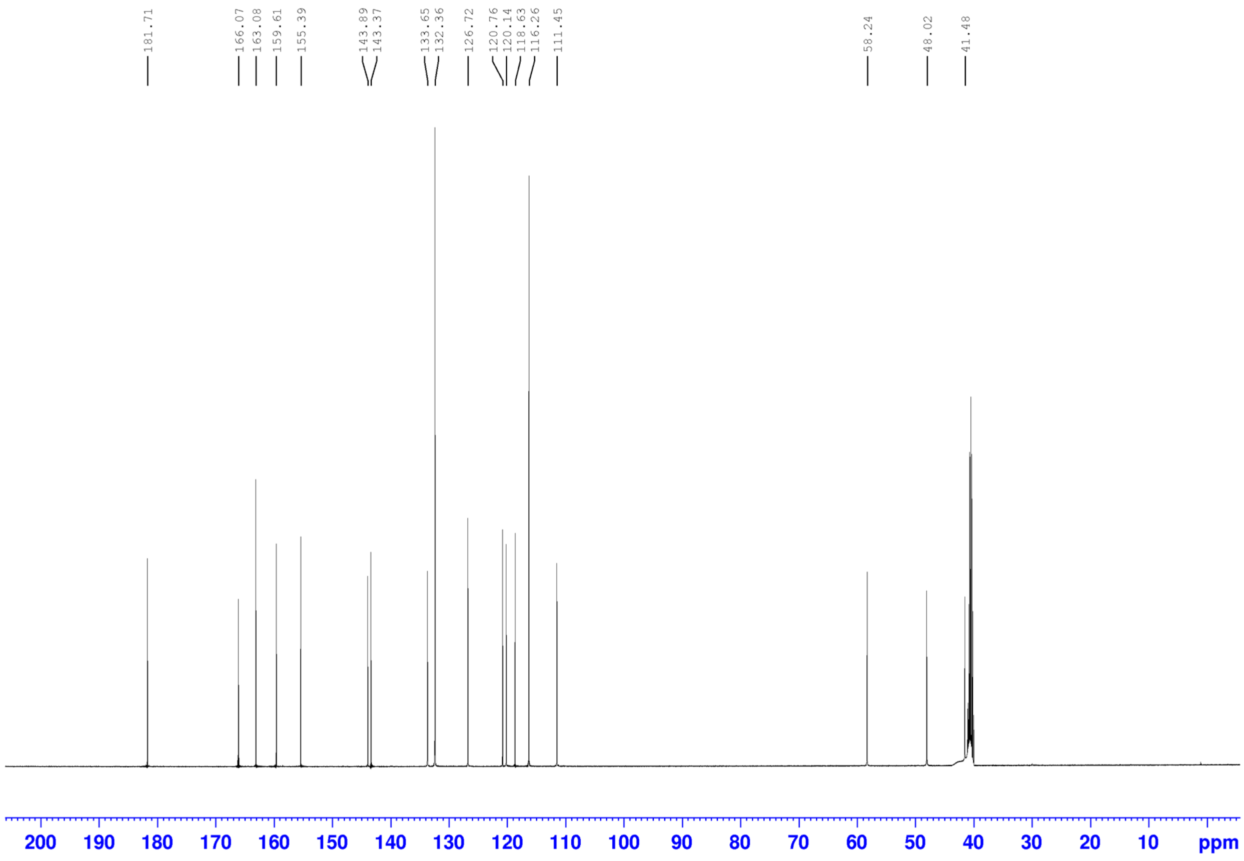

COSY NMR (500 MHz)

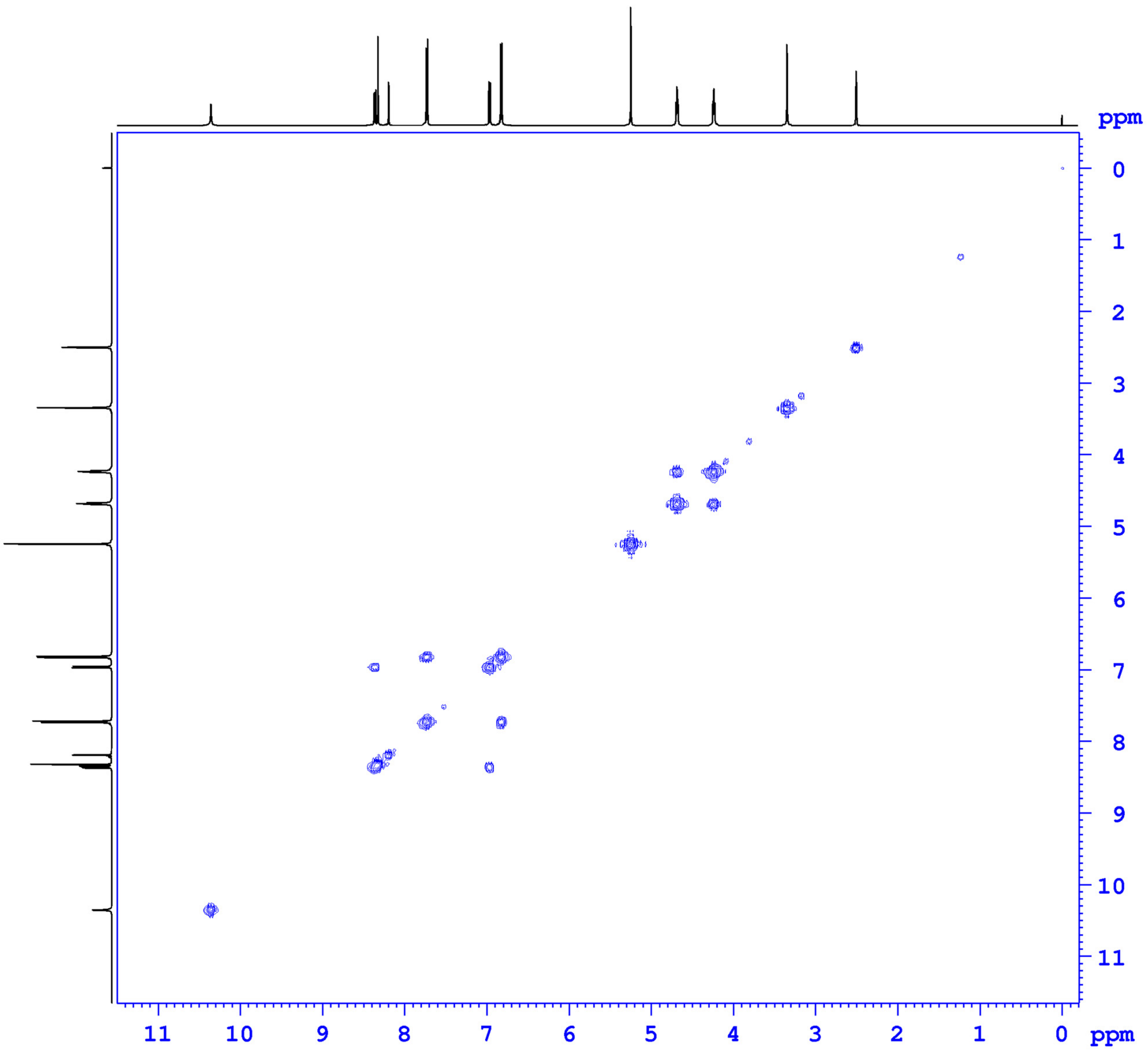

HRMS

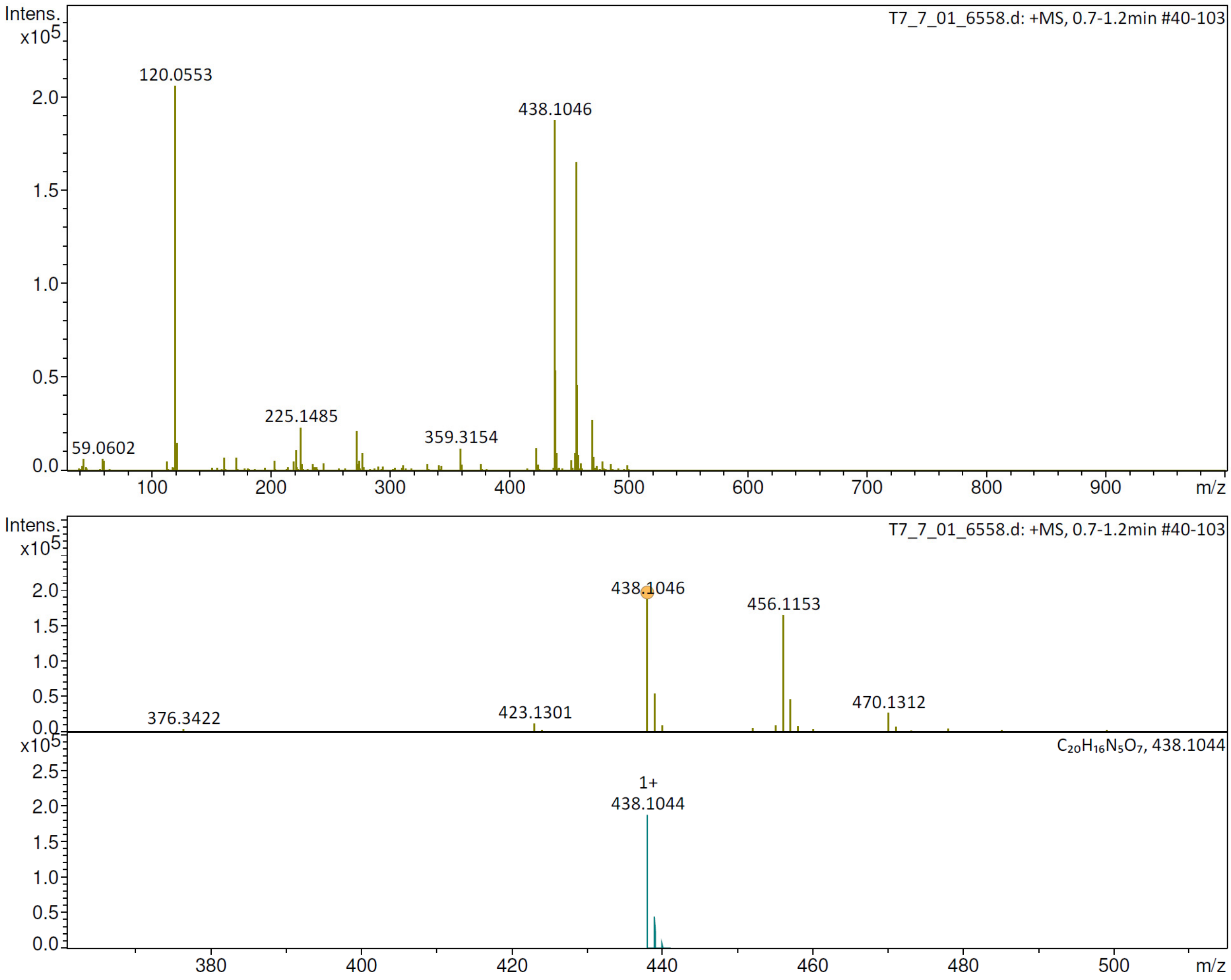
Intens.
x10^5
T7_7_01_6558.d: +MS, 0.7-1.2min #40-103
120.0553
438.1046
225.1485
359.3154
59.0602
m/z
T7_7_01_6558.d: +MS, 0.7-1.2min #40-103
438.1046
456.1153
470.1312
423.1301
376.3422
C20H16N5O7, 438.1044
1+
438.1044

(1-(2-(5-chloro-isatin-1-yl)ethyl)-1,2,3-triazol-4-yl)methyl 4-hydroxybenzoate (30)

^{1}H NMR (300 MHz)

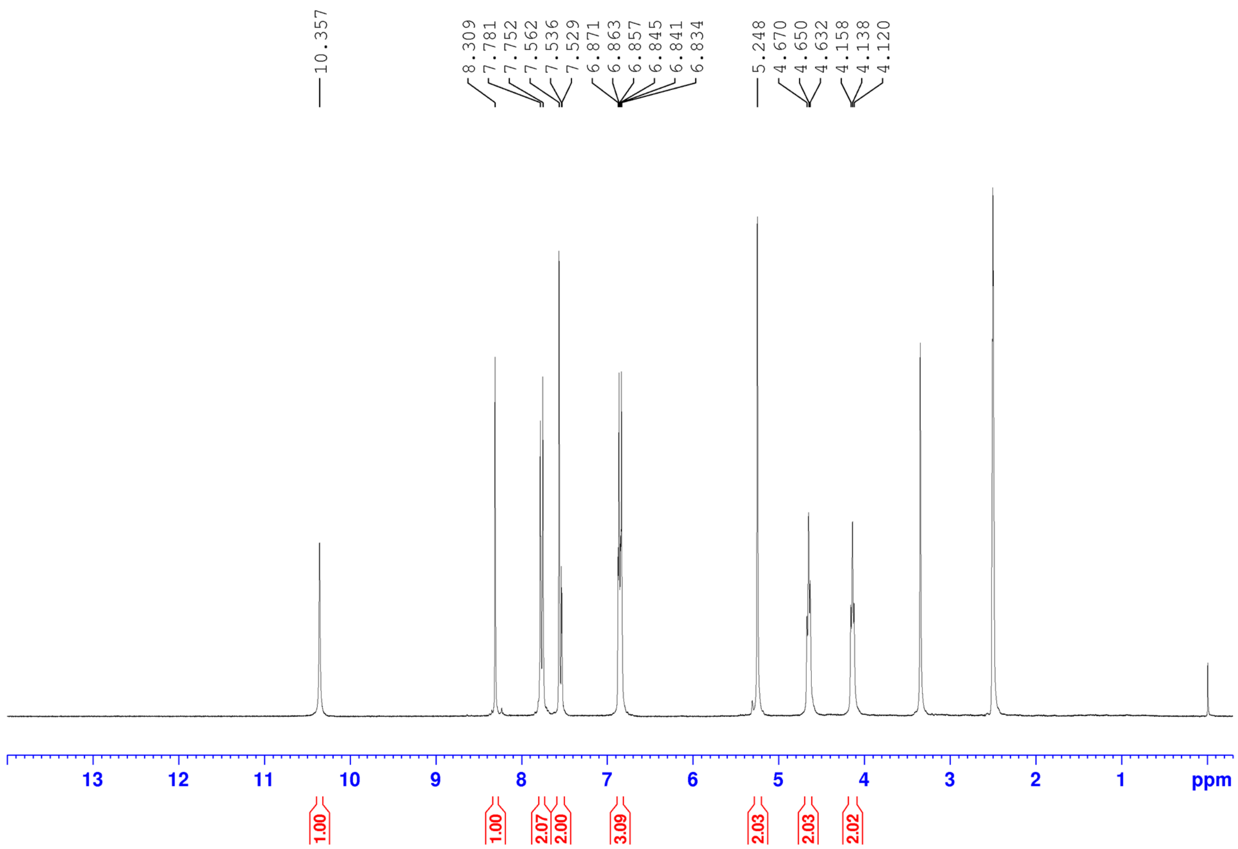

^{13}C NMR (125 MHz)

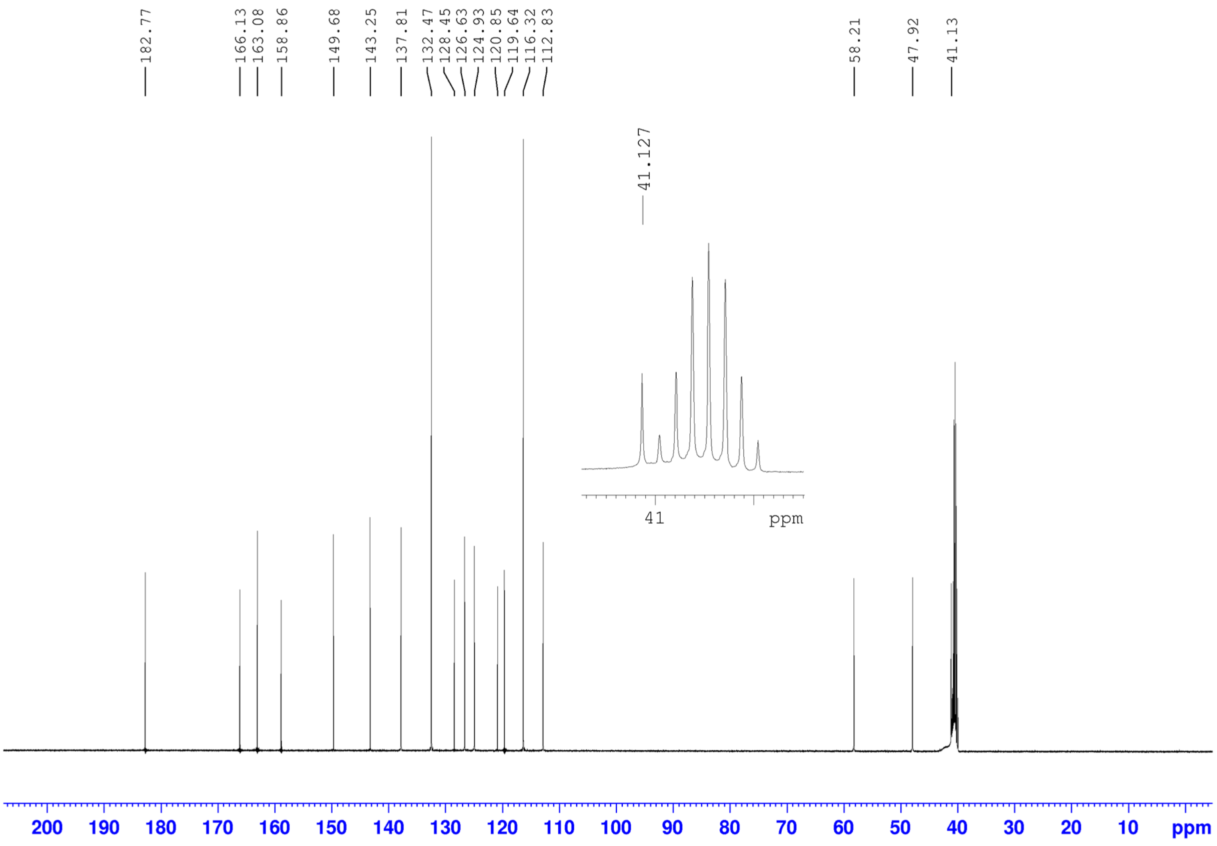

COSY NMR (300 MHz)

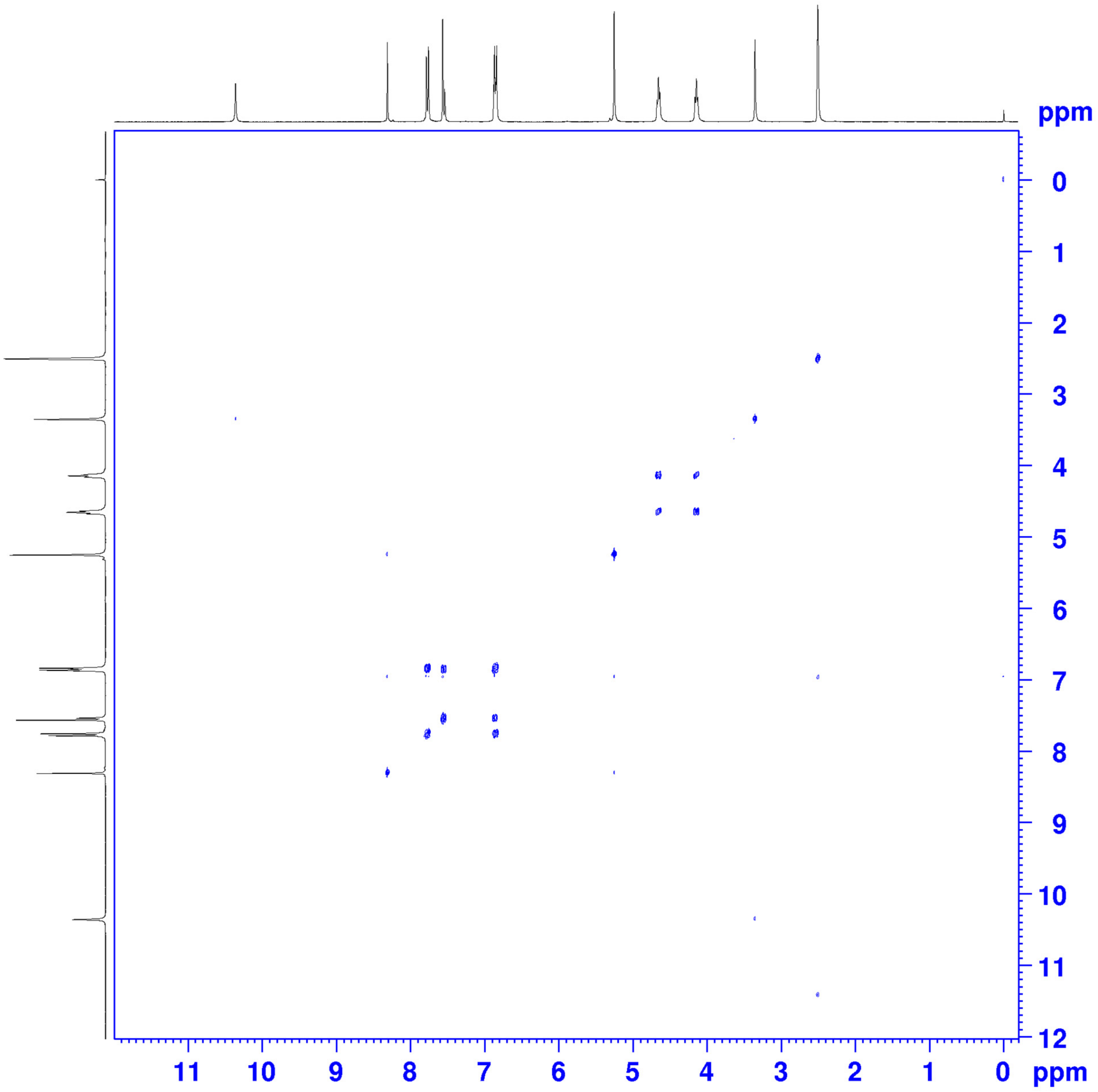

HRMS

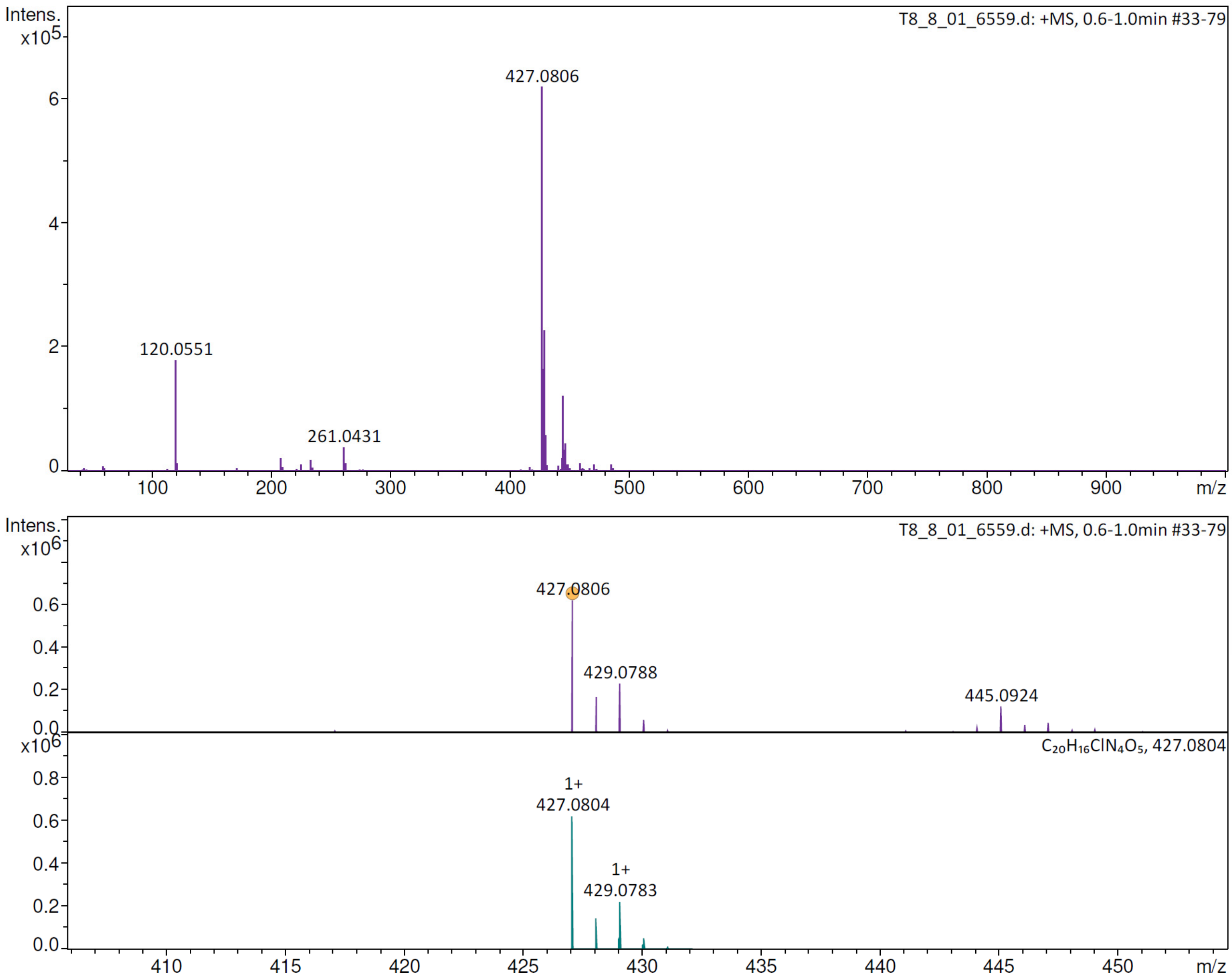
Intens.
x10^5
T8_8_01_6559.d: +MS, 0.6-1.0min #33-79
427.0806
120.0551
261.0431
m/z
Intens.
x10^6
T8_8_01_6559.d: +MS, 0.6-1.0min #33-79
427.0806
429.0788
445.0924
x10^6
C20H16ClN4O5, 427.0804
1+
427.0804
1+
429.0783
m/z

(1-(2-(isatin-1-yl)ethyl)-1,2,3-triazol-4-yl)methyl (E)-3-(3,4-dihydroxyphenyl)acrylate (31)

^{1}H NMR (300 MHz)

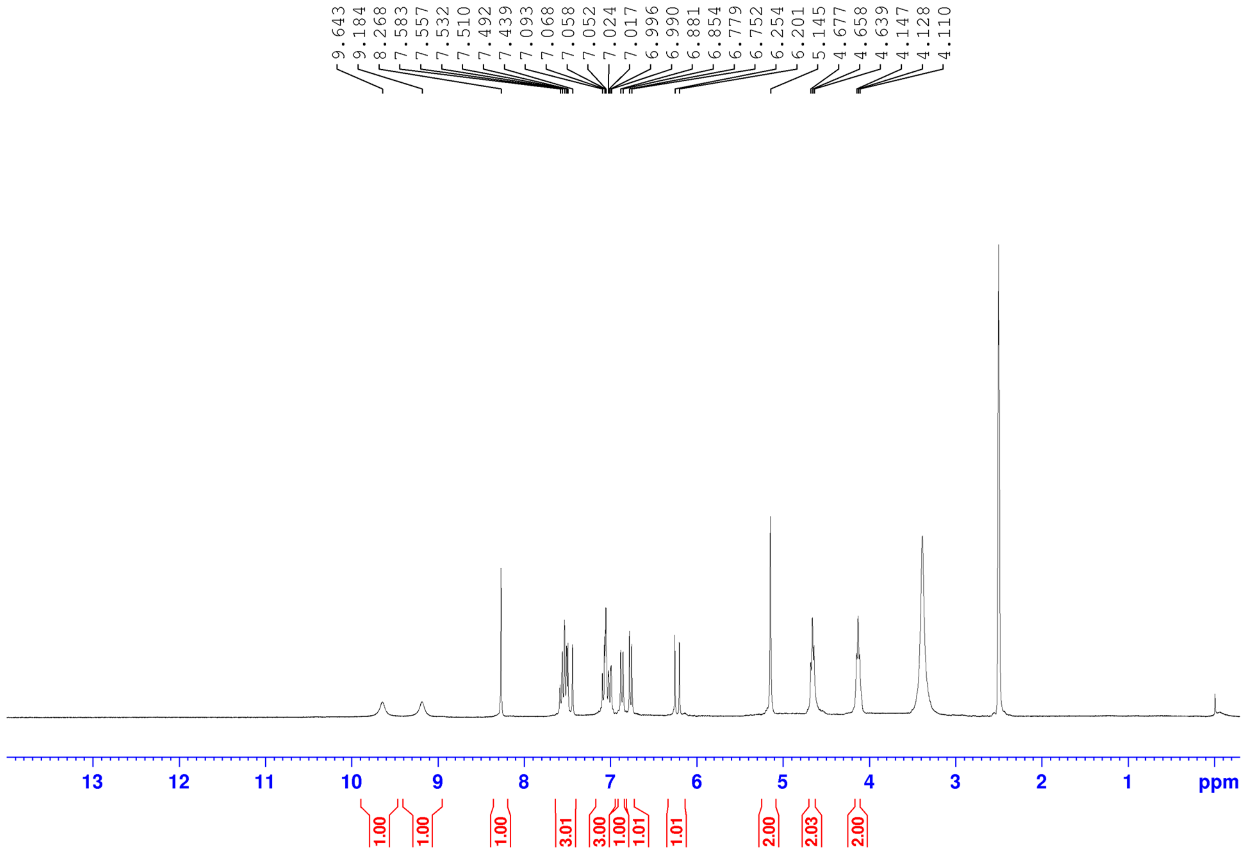

^{13}C NMR (125 MHz)

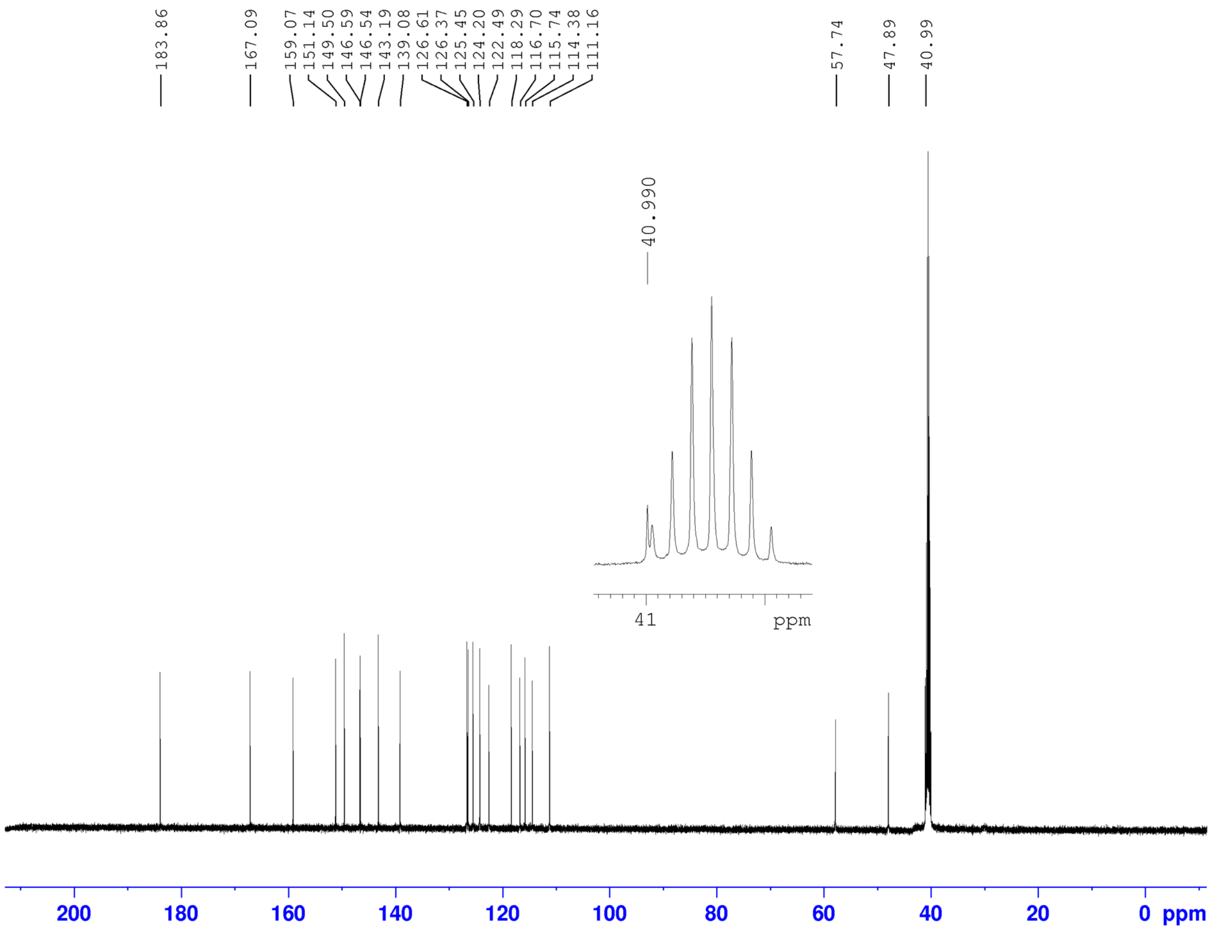

COSY NMR (300 MHz)

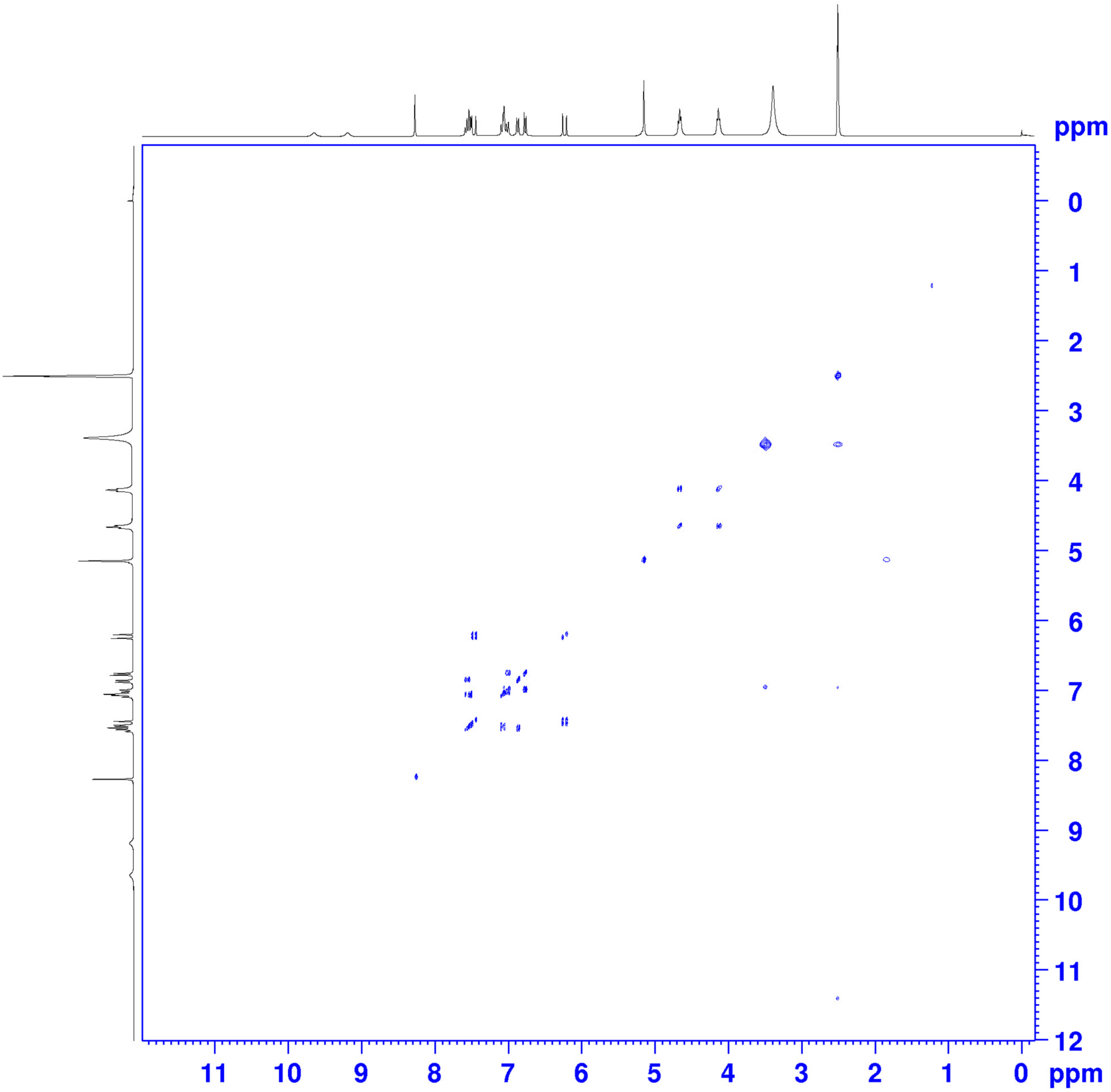

HRMS

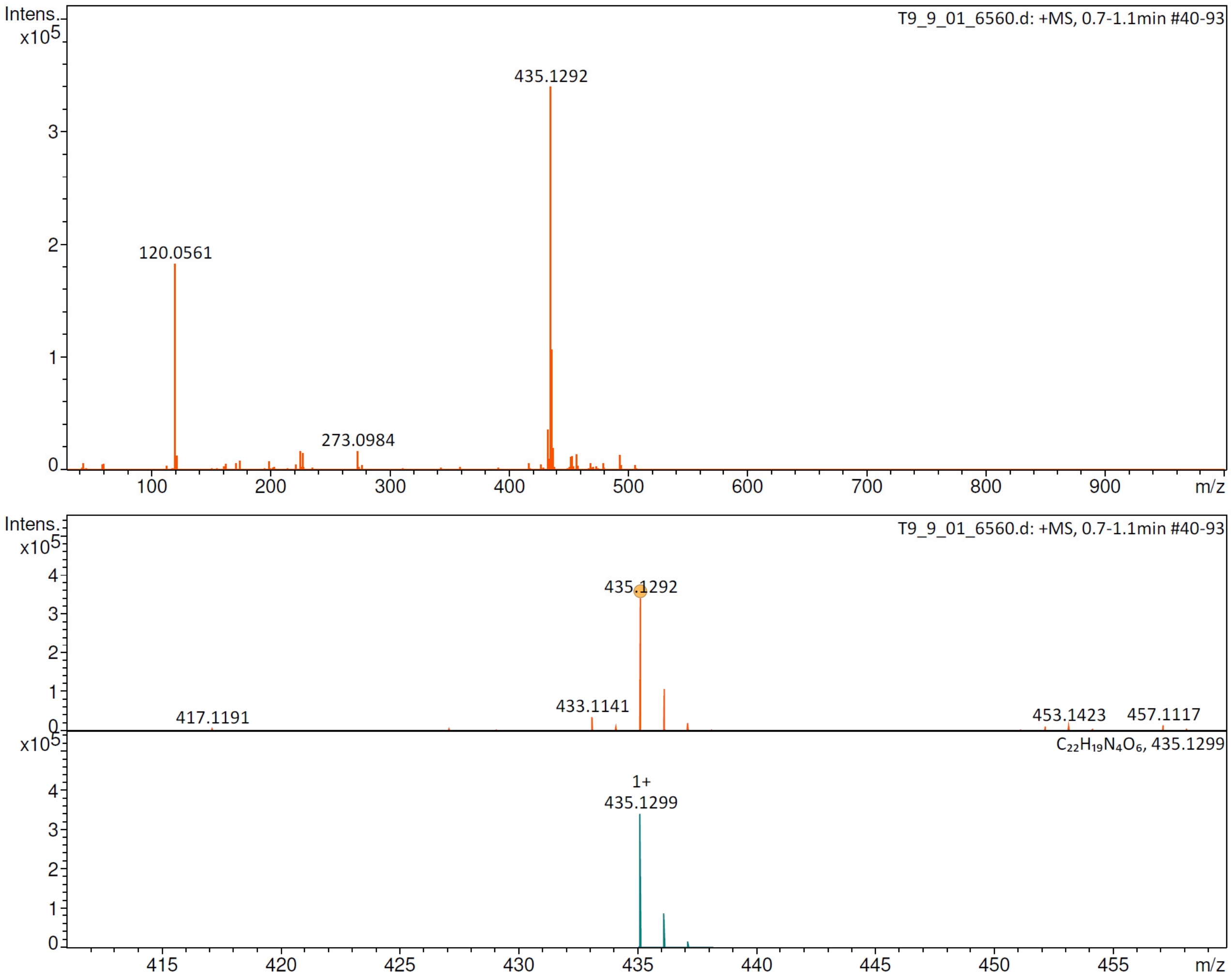

Intens.
x10^5
T9_9_01_6560.d: +MS, 0.7-1.1min #40-93
435.1292
120.0561
273.0984
m/z
Intens.
x10^5
T9_9_01_6560.d: +MS, 0.7-1.1min #40-93
435.1292
417.1191
433.1141
453.1423
457.1117
x10^5
C22H19N4O6, 435.1299
1+
435.1299
m/z

(1-(2-(5-methoxyisatin-1-yl)ethyl)-1,2,3-triazol-4-yl)methyl (E)-3-(3,4-dihydroxyphenyl)acrylate (32)

^{1}H NMR (500 MHz)

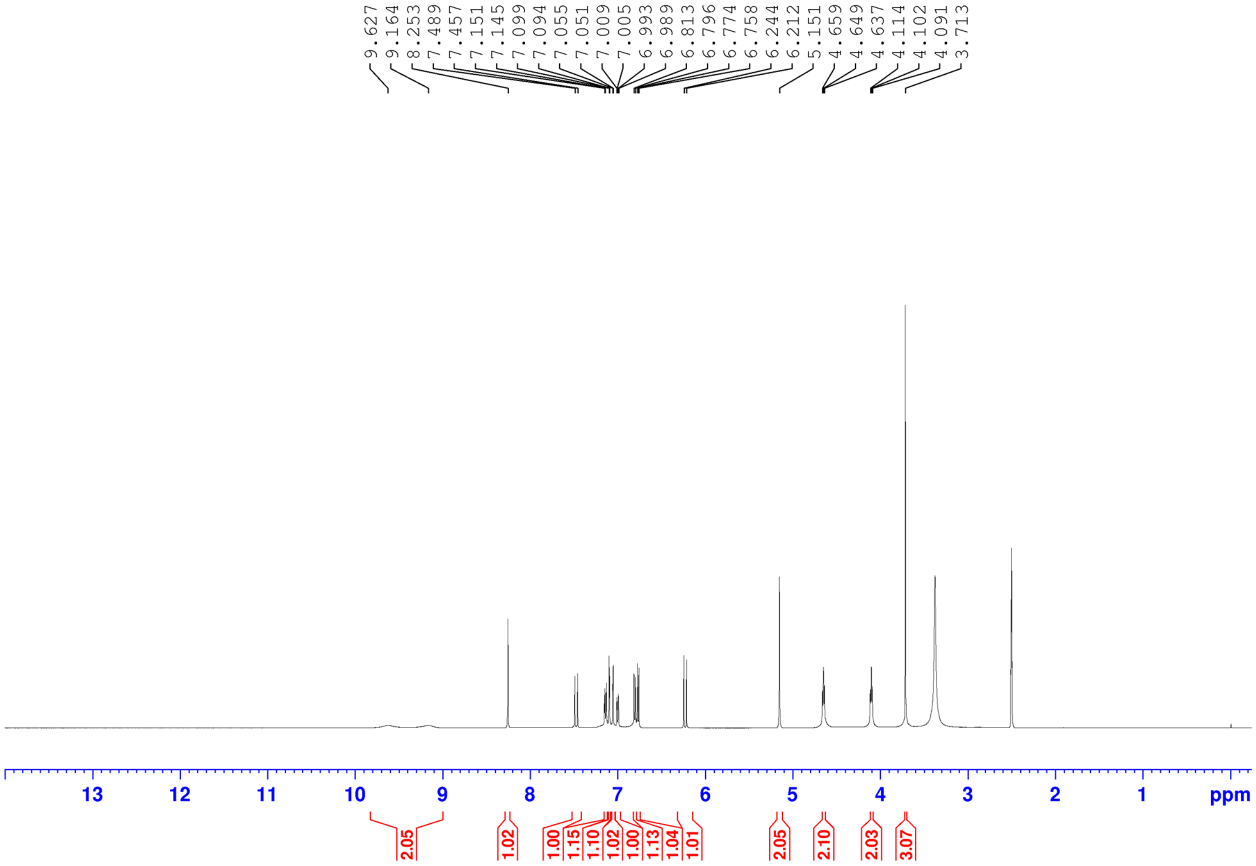

^{13}C NMR (125 MHz)

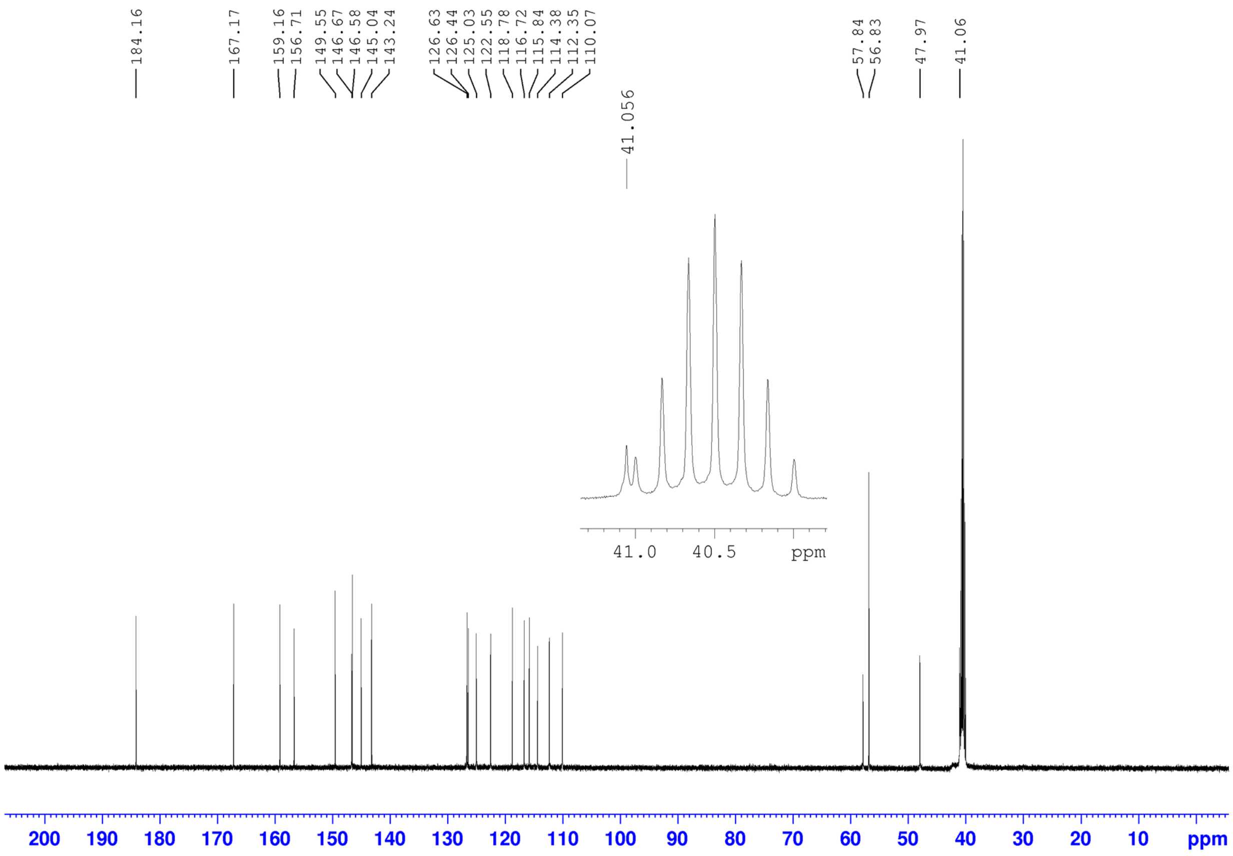

COSY NMR (300 MHz)

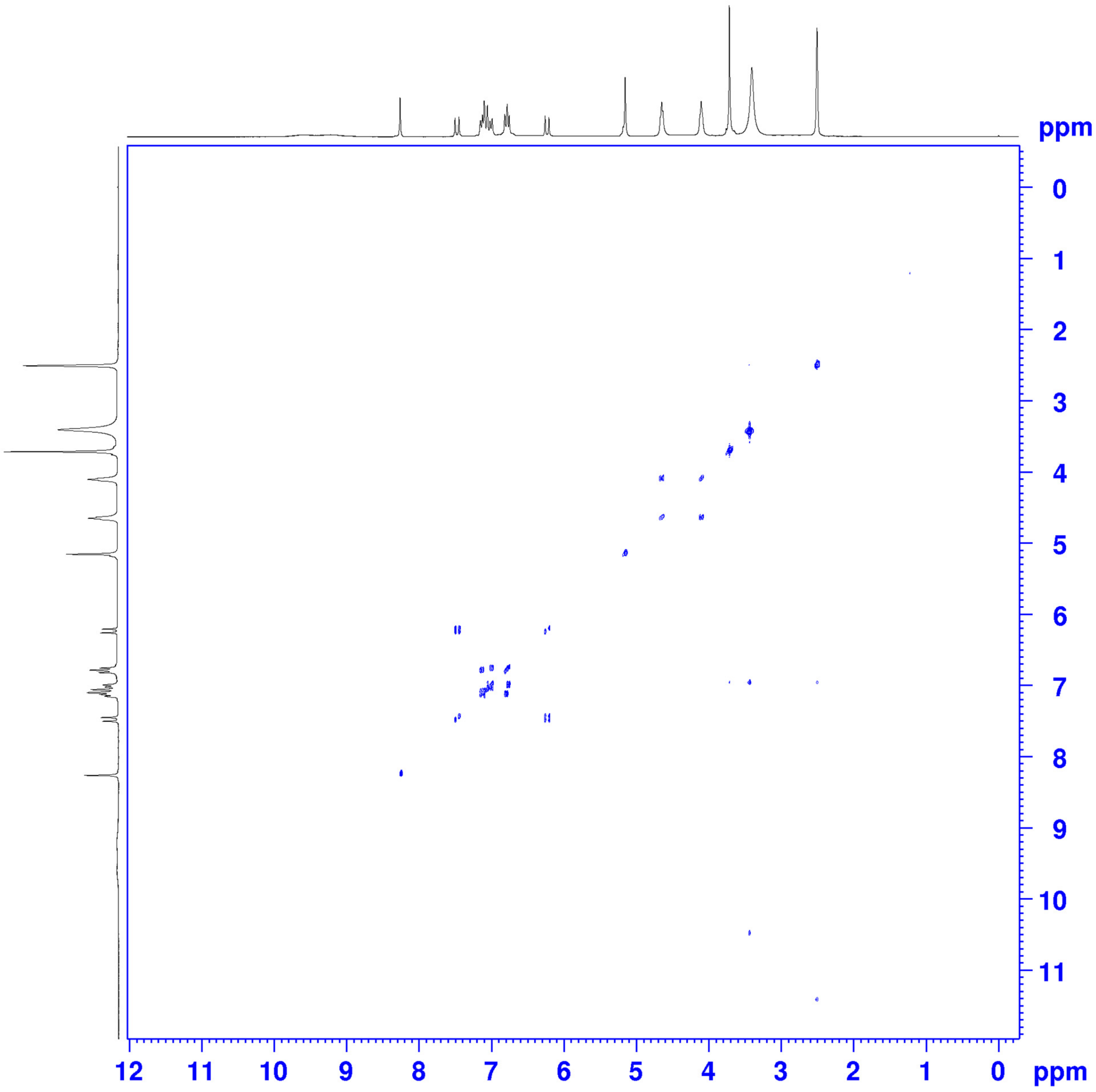

HRMS

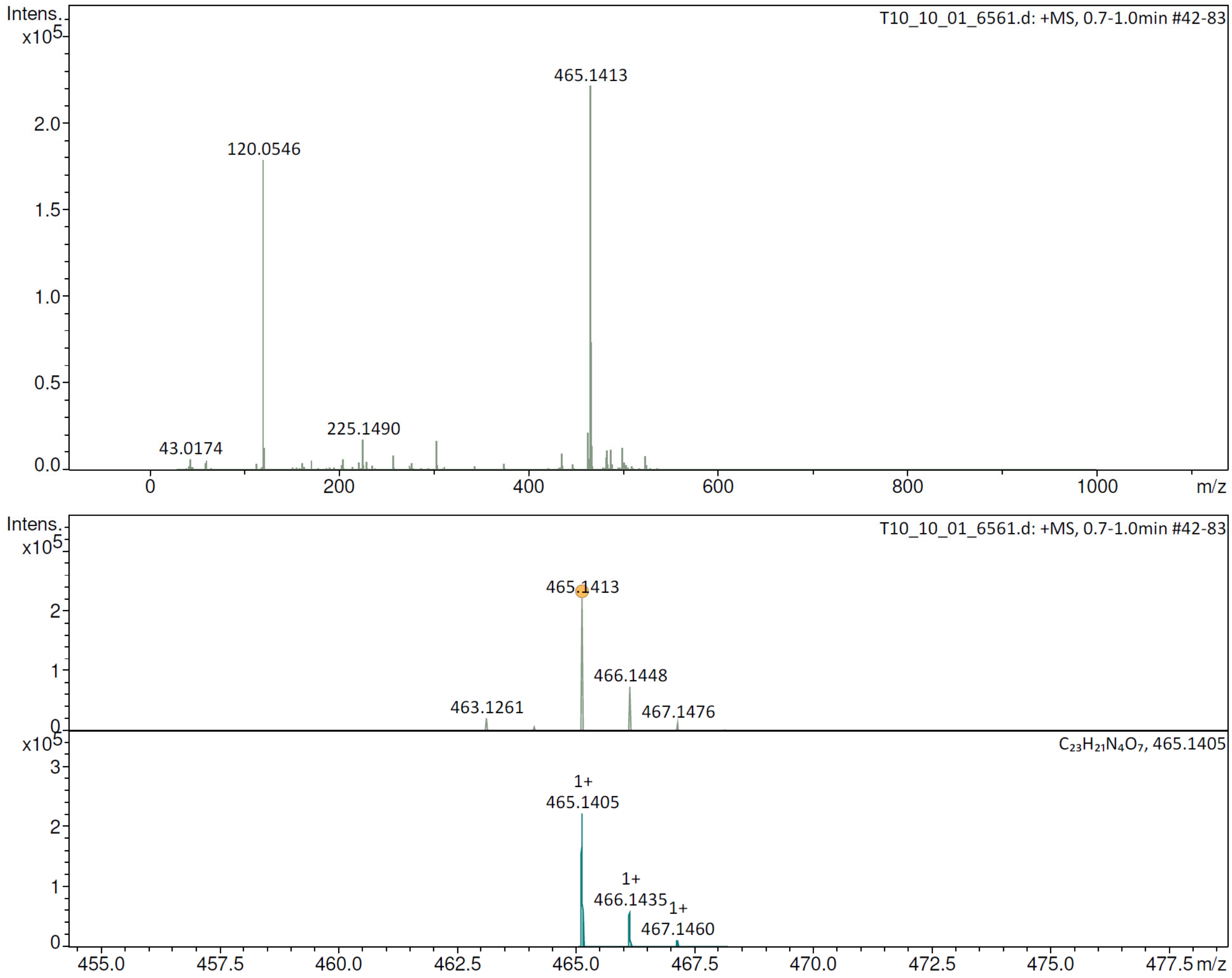

Intens.
x10^5
T10_10_01_6561.d: +MS, 0.7-1.0min #42-83
465.1413
120.0546
225.1490
43.0174
2.0
1.5
1.0
0.5
0.0
0
200
400
600
800
1000
m/z
Intens.
x10^5
T10_10_01_6561.d: +MS, 0.7-1.0min #42-83
465.1413
466.1448
463.1261
467.1476
x10^5
C23H21N4O7, 465.1405
1+
465.1405
1+
466.1435
1+
467.1460
455.0
457.5
460.0
462.5
465.0
467.5
470.0
472.5
475.0
477.5 m/z

(1-(2-(5-nitro-isatin-1-yl)ethyl)-1,2,3-triazol-4-yl)methyl (E)-3-(3,4-dihydroxyphenyl)acrylate (33)

^{1}H NMR (500 MHz)

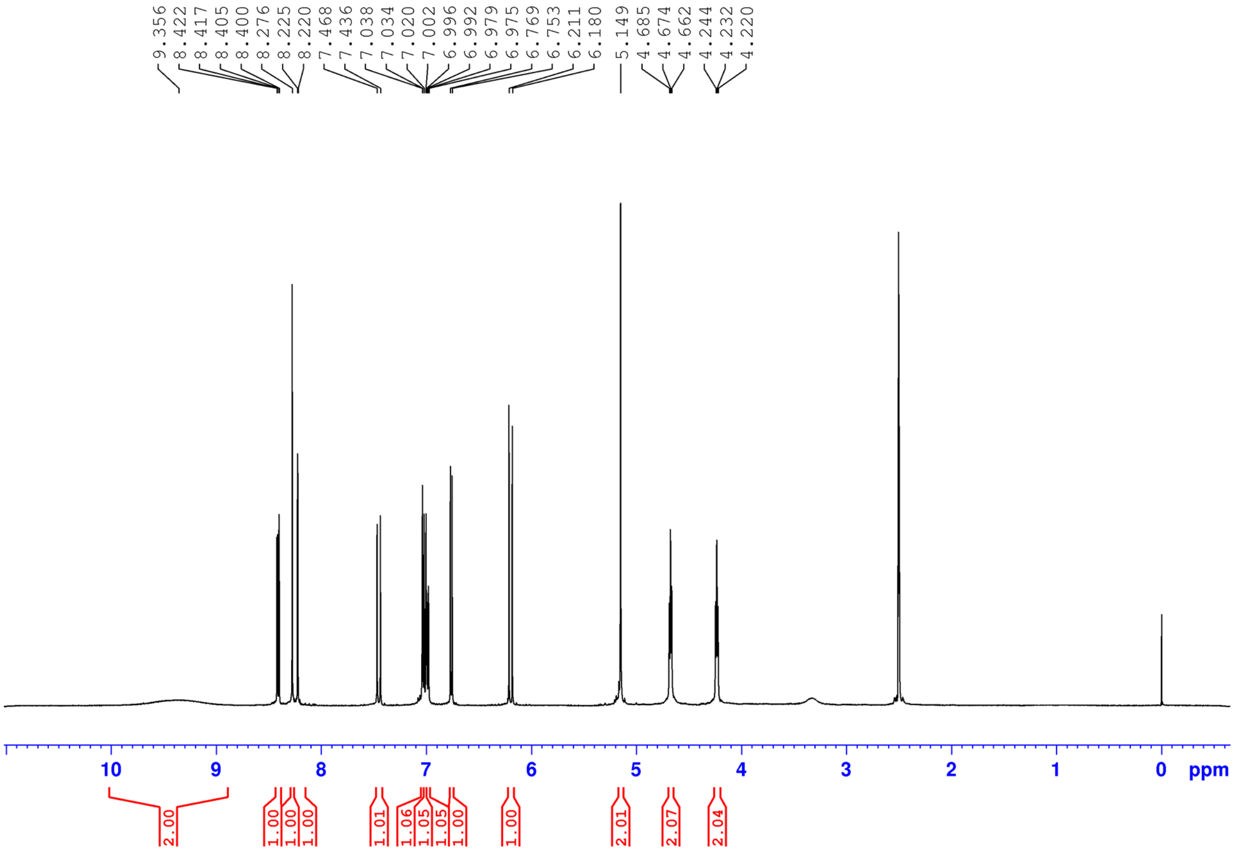

^{13}C NMR (125 MHz)

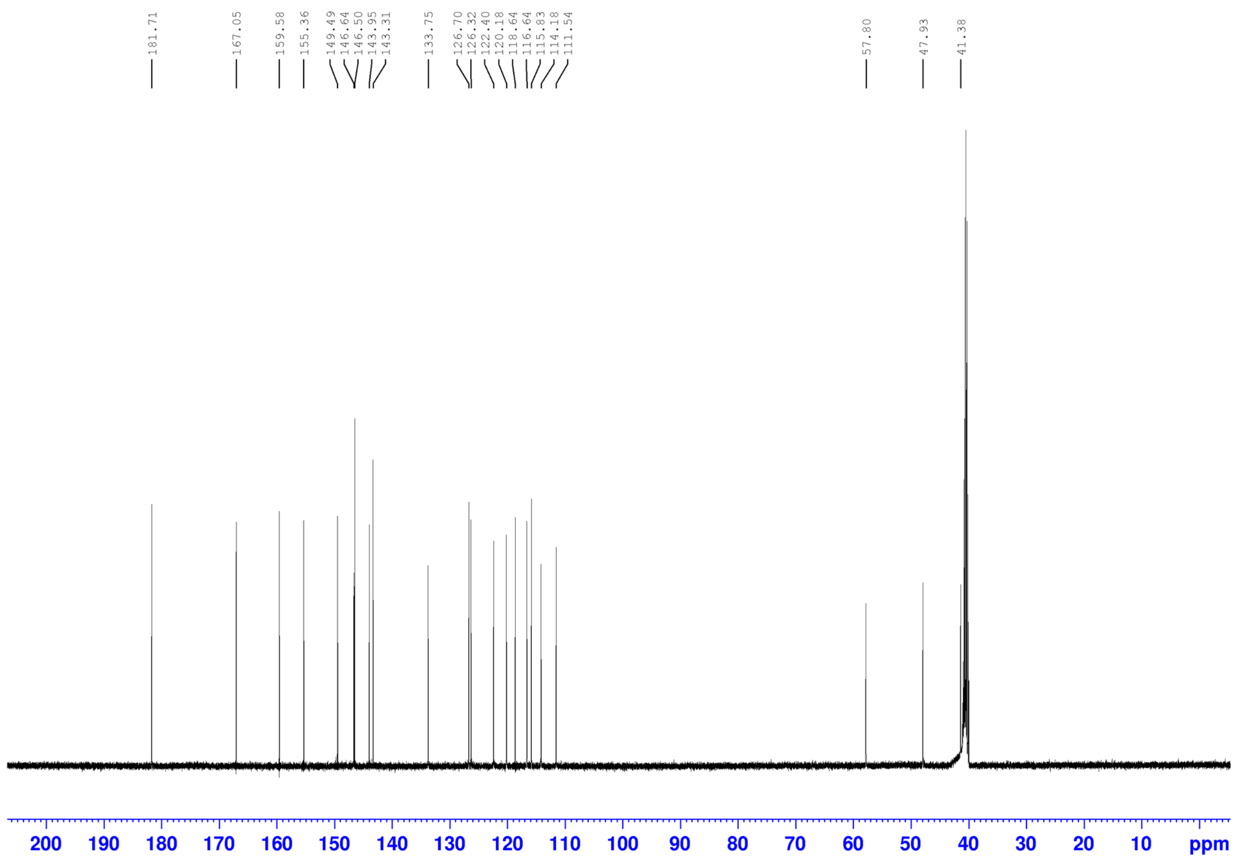

COSY NMR (500 MHz)

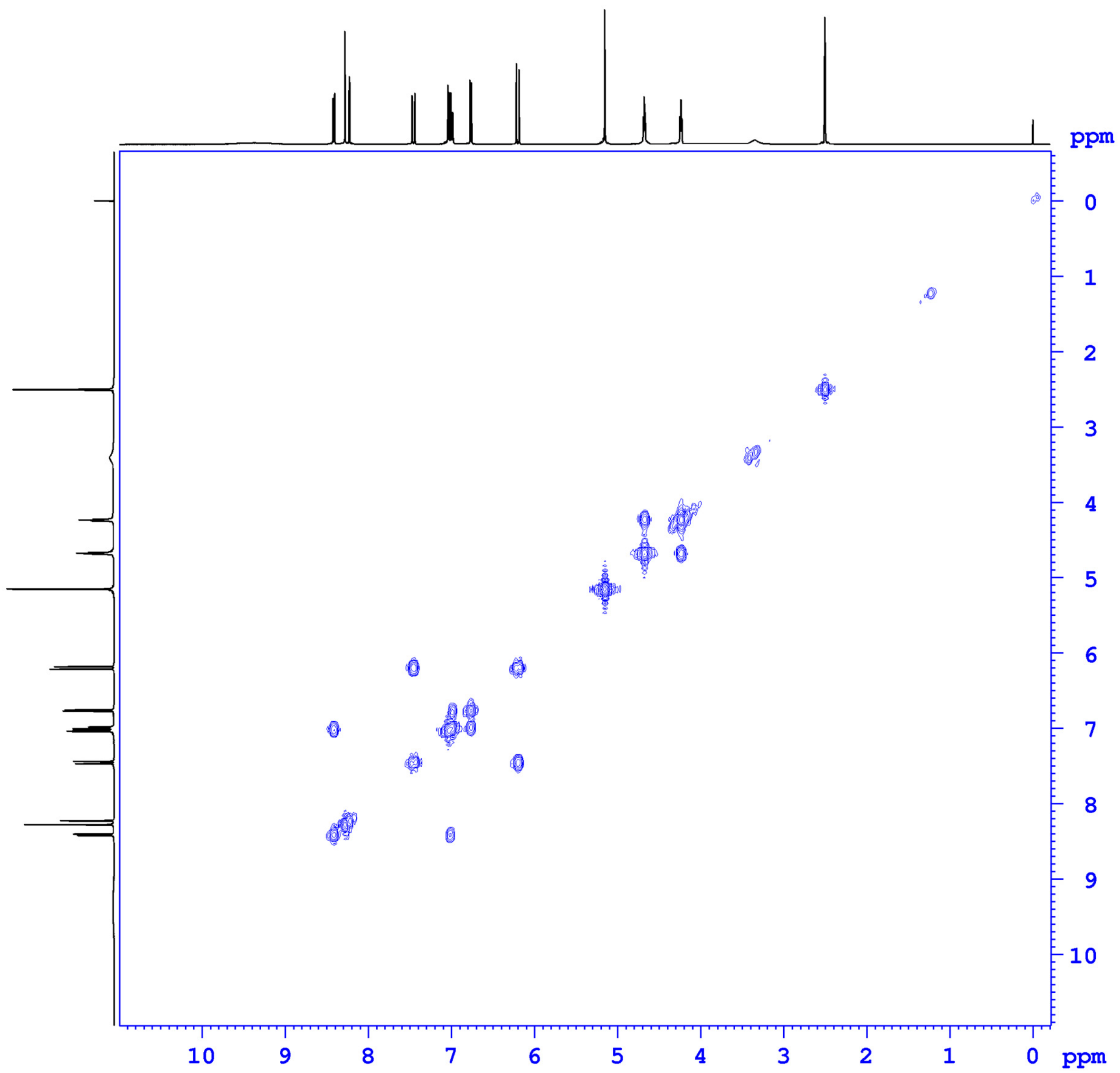

HRMS

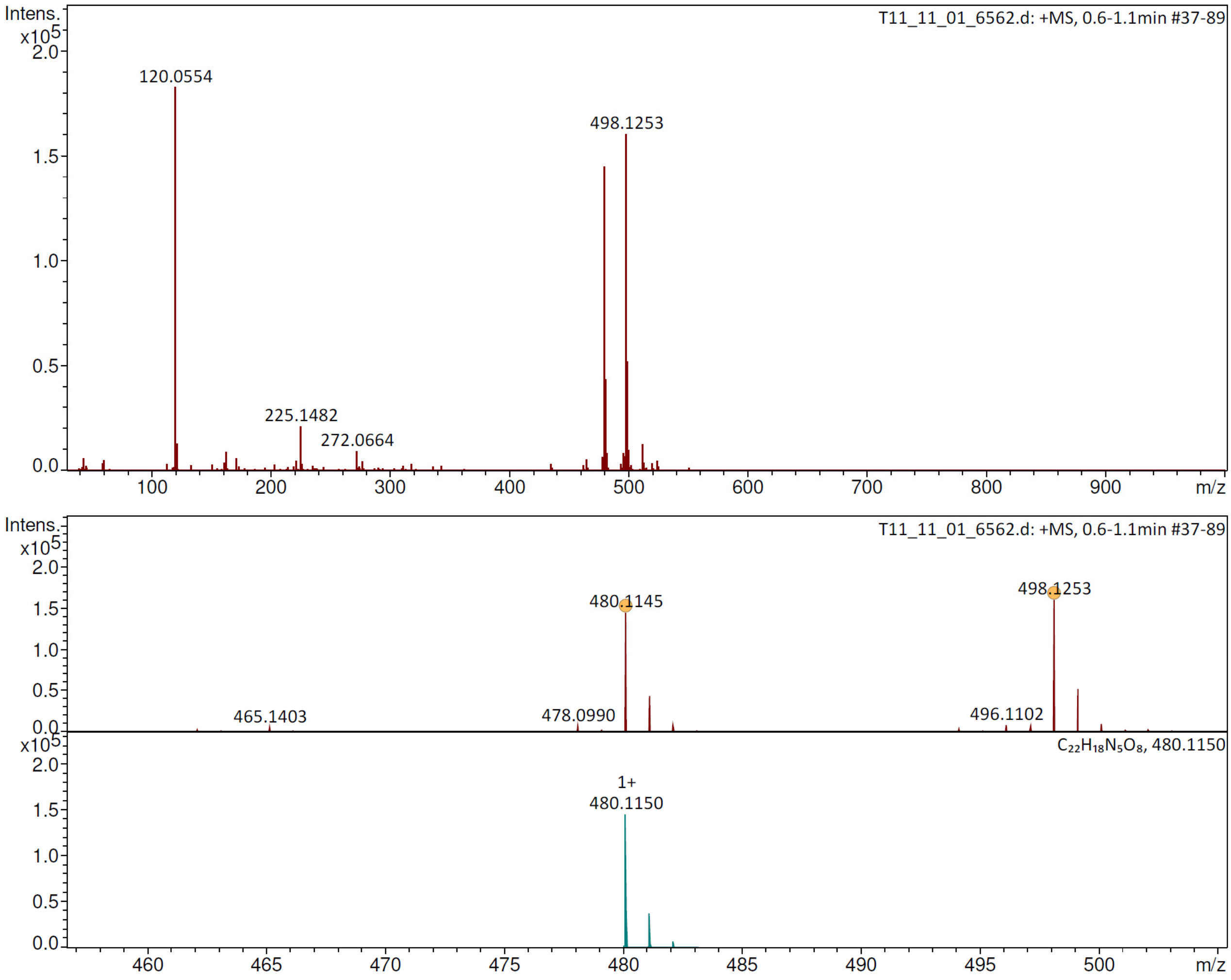

Intens.
x10^5
T11_11_01_6562.d: +MS, 0.6-1.1min #37-89
120.0554
498.1253
225.1482
272.0664
m/z
480.1145
498.1253
465.1403
478.0990
496.1102
C22H18N5O8, 480.1150
1+
480.1150

(1-(2-(5-chloro-isatin-1-yl)ethyl)-1,2,3-triazol-4-yl)methyl (E)-3-(3,4-dihydroxyphenyl)acrylate (34)

^{1}H NMR (300 MHz)

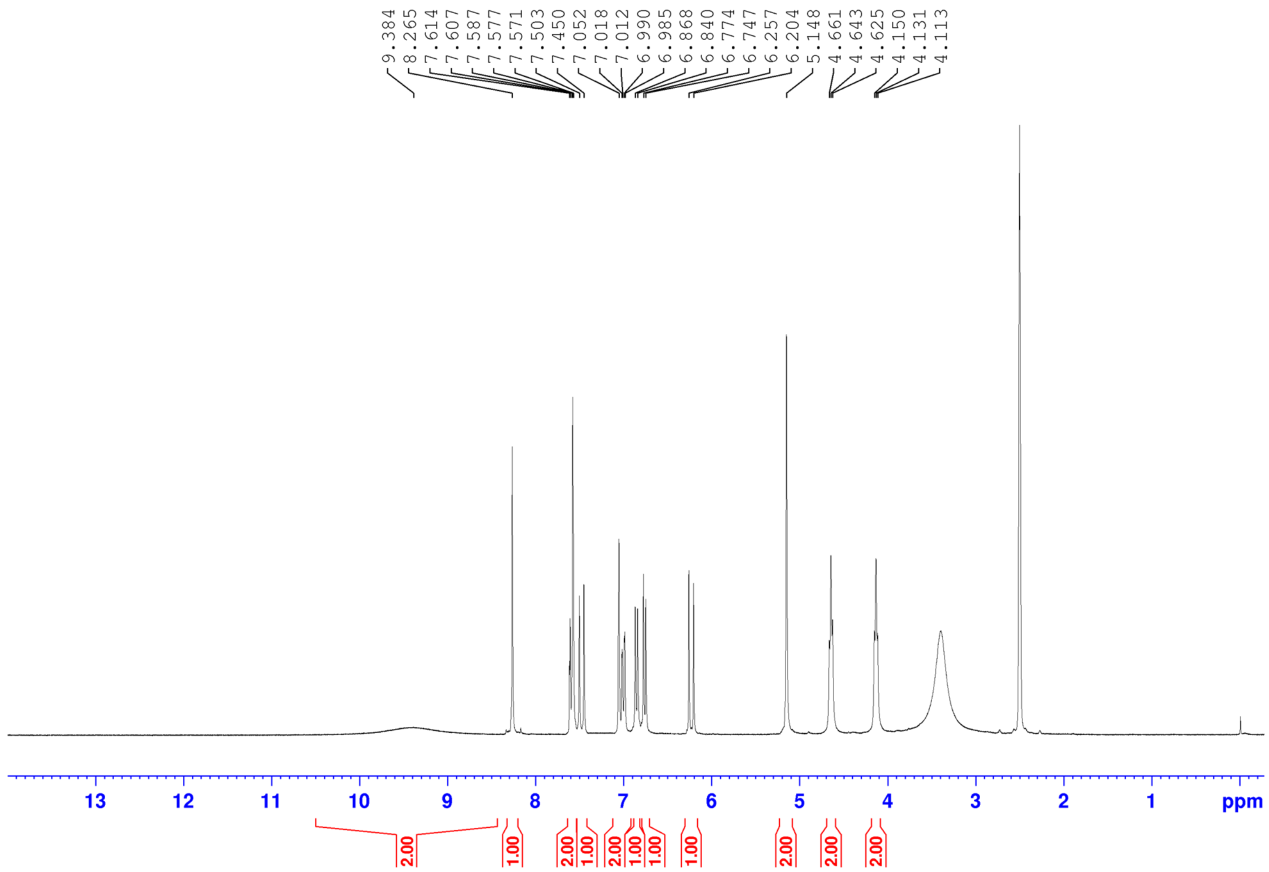

^{13}C NMR (125 MHz)

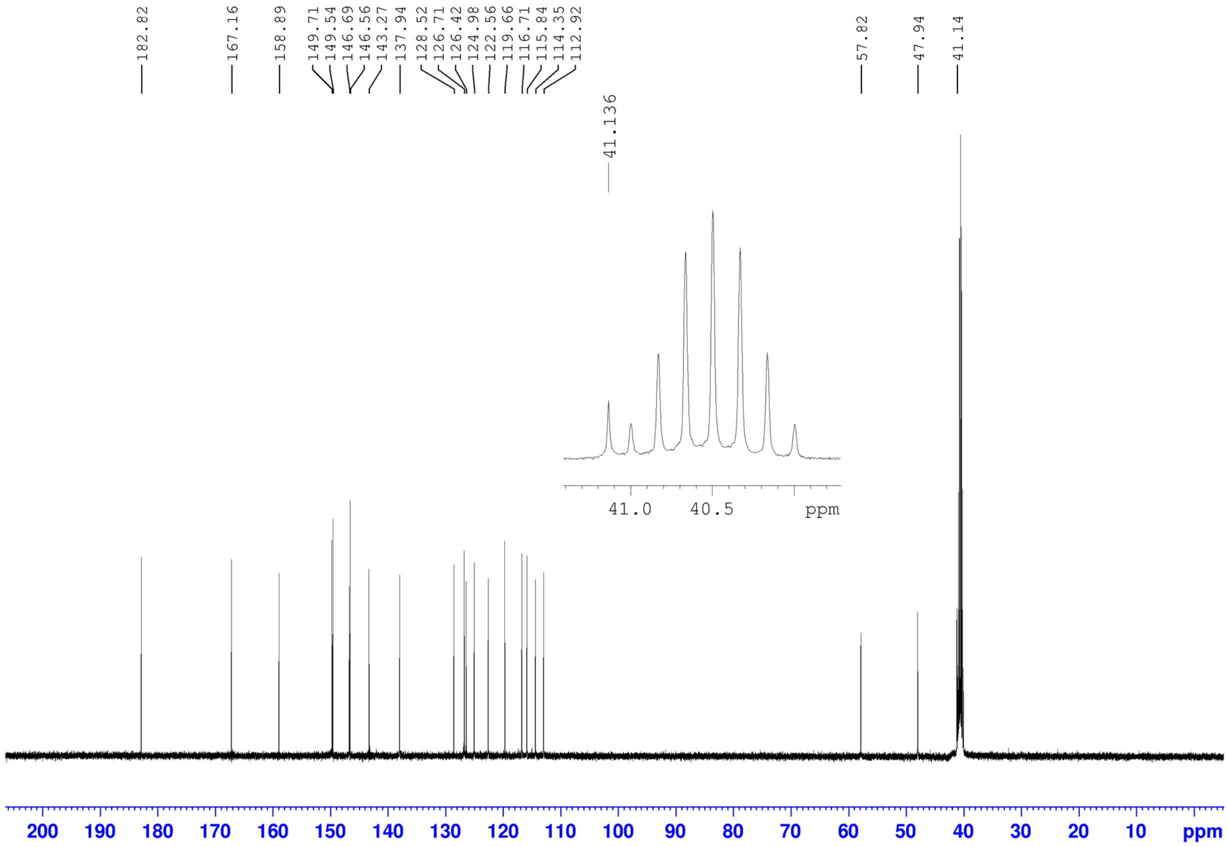

COSY NMR (300 MHz)

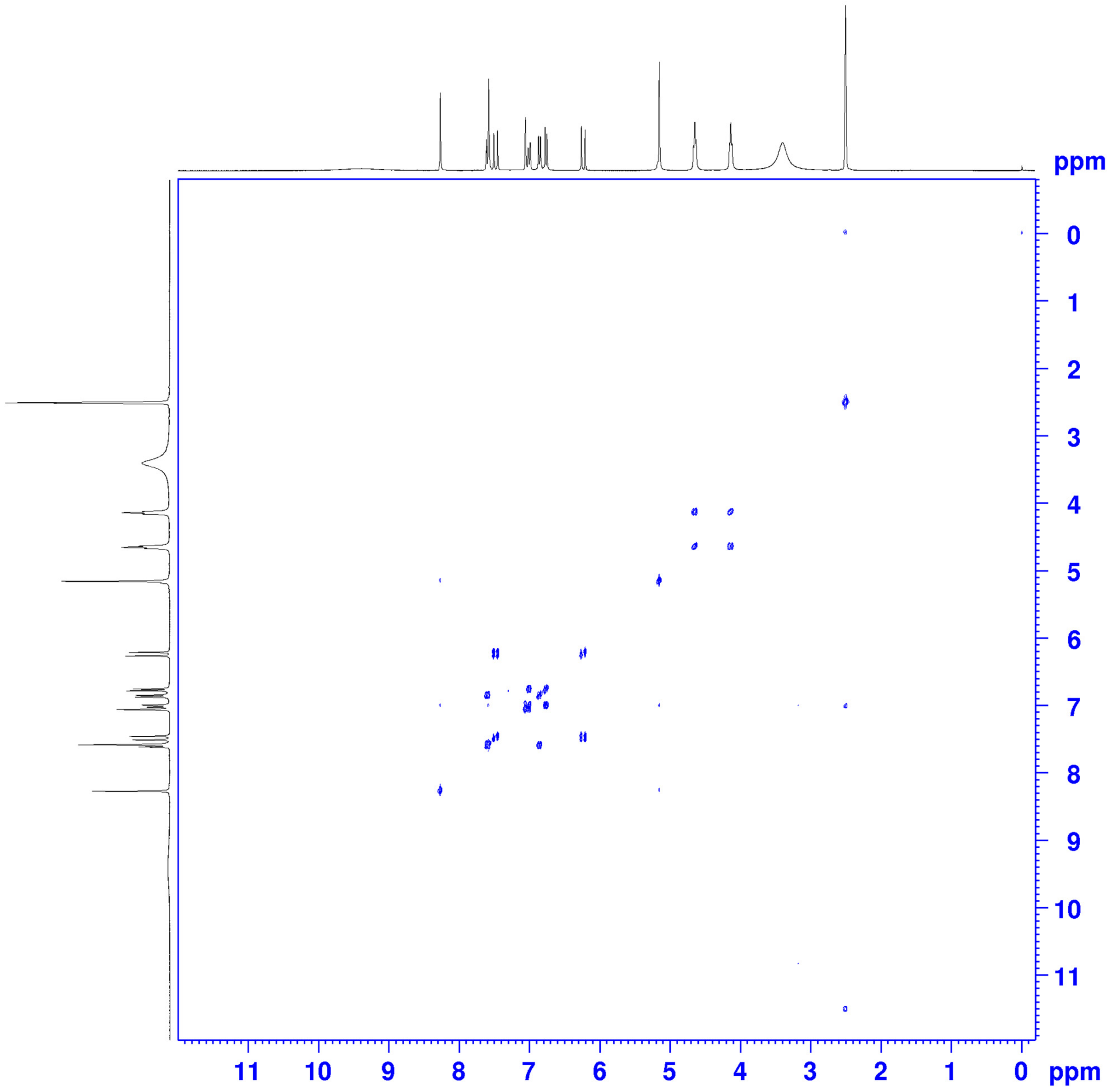

HRMS

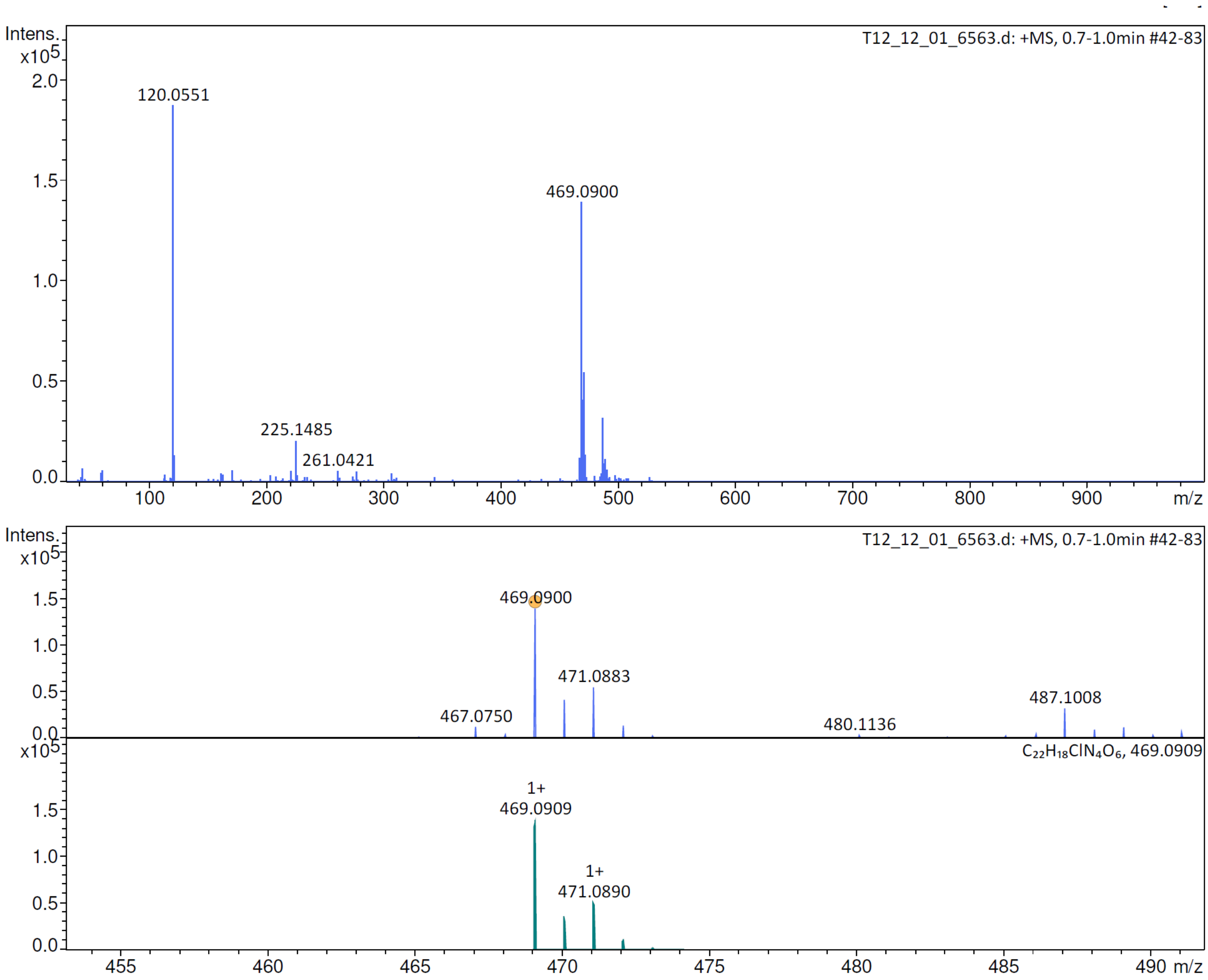
Intens.
x10^5
T12_12_01_6563.d: +MS, 0.7-1.0min #42-83
120.0551
469.0900
225.1485
261.0421
m/z
C22H18ClN4O6, 469.0909
467.0750
471.0883
480.1136
487.1008
1+
469.0909
471.0890
